# Implementing integrated services for people with epilepsy in primary care in Ethiopia: a qualitative study

**DOI:** 10.1186/s12913-018-3190-y

**Published:** 2018-05-21

**Authors:** Raquel Catalao, Tigist Eshetu, Ruth Tsigebrhan, Girmay Medhin, Abebaw Fekadu, Charlotte Hanlon

**Affiliations:** 1 Camden and Islington NHS Foundation Trust, London, UK; 2 King’s College London, Institute of Psychiatry, Psychology and Neuroscience, Health Service and Population Research Department, Centre for Global Mental Health, London, UK; 3 Addis Ababa University, College of Health Sciences, School of Medicine, Department of Psychiatry, Addis Ababa, Ethiopia; 4 Addis Ababa University, Aklilu-Lemma Institute of Pathobiology, Addis Ababa, Ethiopia; 5 Centre for Innovative Drug Development and Therapeutic Trials for Africa (CDT-Africa), College of Health Sciences, Addis Ababa University, Addis Ababa, Ethiopia; 6 Global Health & Infection Department, Brighton and Sussex Medical School, Brighton, UK

**Keywords:** Epilepsy, Implementation, Task-sharing, Primary health services, Community health care, mhGAP, Sub-Saharan Africa

## Abstract

**Background:**

In order to tackle the considerable treatment gap for epilepsy in many low- and middle-income countries (LMICs), a task sharing model is recommended whereby care is integrated into primary health services. However, there are limited data on implementation and impact of such services in LMICs. Our study aimed to explore the perspectives of service users and caregivers on the accessibility, experience and perceived impact of epilepsy treatment received in a task-shared model in a rural district of Ethiopia.

**Methods:**

A qualitative study was carried out using interviews with purposively sampled service users (*n* = 13) and caregivers (*n* = 3) from a community-ascertained cohort of people with epilepsy receiving integrated services in primary care in rural Ethiopia. Interviews followed a topic guide with questions regarding acceptability, satisfaction, barriers to access care, pathways through care and impact of services. Framework analysis was employed to analyse the data.

**Results:**

Proximity of the new service in local primary health centers decreased the cost of transportation for the majority of service users thus improving access to services. First-hand experience of services was in some cases associated with a willingness to promote the services and inform others of the existence of effective biomedical treatment for epilepsy. However, most service users and their caregivers continued to seek help from traditional healers alongside biomedical care. Most of the care received was focused on medication provision with limited information provided on how to manage their illness and its effects. Caregivers and service users spoke about the high emotional and financial burden of the disease and lack of ongoing practical and emotional support. The majority of participants reported clinical improvement on medication, which in over half of the participants was associated with ability to return to money generating activities.

**Conclusions:**

Task-sharing improved the accessibility of epilepsy care for services users and caregivers and was perceived as having a positive impact on symptoms and productivity. Nonetheless, promotion of self-management, holistic care and family engagement were highlighted as areas requiring further improvement. Future work on implementing chronic care models in LMIC contexts is warranted.

**Electronic supplementary material:**

The online version of this article (10.1186/s12913-018-3190-y) contains supplementary material, which is available to authorized users.

## Background

Epilepsy is a chronic disease of the brain characterized by recurrent unprovoked seizures which can have an extensive and multifaceted impact on a person’s life [1]. The incidence and prevalence of epilepsy has been reported to be considerably higher in Low and Middle Income Countries (LMICs) compared to High Income Countries (HIC) [2, 3]. There are no recent prevalence studies of epilepsy in Ethiopia: the best estimate from a large scale community-based survey in a rural setting is 5.2 per 1000 people [4].

The treatment gap for epilepsy in LMICs is considerable. Overall, the median epilepsy treatment gap for active epilepsy ranges from 25 to 100%, compared to less than 10% in HICs. There is substantial heterogeneity across and within countries, with treatment gaps higher in rural than urban areas [5–7]. In a recent community study, the majority of people with epilepsy (PWE) in a rural district in Ethiopia were found to seek help from both religious and biomedical healing centres, with a lifetime treatment gap for biomedical care of 26.9%, and a 12 month treatment gap of 56.7% [8].

The World Health Organization launched the mental health Gap Action Programme (mhGAP) to address the high treatment gap for priority mental, neurological and substance use (MNS) disorders in LMICs [9]. Epilepsy is grouped with mental health in the priority disorders for several reasons.: there is substantial stigma associated with epilepsy and in some communities the aetiology is attributed to supernatural causes [10]; epilepsy tends to be treated by mental health specialists in LMICs [5] and PWE are also at increased risk of psychiatric comorbidity compared to the general population [8, 11]. The focus of WHO’s mhGAP is the integration of care for people with MNS disorders into primary care using a task-sharing model [9]. Primary care workers are given brief training in evidence-based packages of care for epilepsy and other priority disorders which have been contextualised to LMIC settings [12]. The Ethiopian Federal Ministry of Health has endorsed this approach to epilepsy care within the National Mental Health Strategy [13].

Despite the existence of evidence-based interventions for epilepsy in primary care there is limited data on how to successfully translate this knowledge into sustainable practice [14]. In Ethiopia, previous studies have shown that it is possible to establish primary care clinics for treatment of epilepsy using the existing healthcare infrastructure and staff with a few additional resources including ongoing health staff training [15]. However high staff turnover in both the health centers and hospital and unreliable medication supplies have hindered the sustainability of this approach [15]. Furthermore, there has been limited evaluation of the implementation, particularly from the perspective of service users.

The aim of our study was therefore to explore the views of service users and their caregivers in a rural Ethiopian district on the accessibility, experience and perceived impact of epilepsy treatment received in a task-shared model.

## Methods

This study was conducted as part of the PRogramme for Improving Mental health carE (PRIME), an implementation research project to investigate the best approaches for integrating care for MNS disorders into primary care at the district level in five LMICs: Ethiopia, India, Nepal, South Africa and Uganda [16]. In Ethiopia, an integrated district level plan was developed to provide primary care-based care for people with psychosis, bipolar disorder, depressive disorders, alcohol use disorder and epilepsy [17]. The district MNS care plan was based on a task-sharing model.

For epilepsy, the PRIME interventions were as follows: community implementation packages included community case detection, community reintegration and inclusion. Facility level packages involved capacity building and decision support using the mhGAP intervention guide (mhGAP-IG). Clinical staff were given theoretical training for one week using the WHO mhGAP-IG [12] as the core training tool to guide diagnosis and provide treatment for MNS disorders in their local primary care clinics. Training included participatory methods such as case scenarios, video clips and an additional five days of clinical exposure in a psychiatric nurse led outpatient clinic in the adjoining district (where people with epilepsy are also treated) where trainees saw over 200 patients. Clinical staff was also provided with decisions aid such as mhGAP-IG pocket guides, posters and ongoing supervision. Supportive supervision was provided both by staff designated to be supervisors within the health system (who were trained in supervision of MNS disorders) as well as by specialist staff (a psychiatric nurse). Organisational packages included programme management to support health system change to accommodate the new programme [17].

### Study setting

The setting for the implementation of the PRIME MNS care plan in Ethiopia was the Sodo district, Gurage Zone, Southern Nation, Nationalities and Peoples’ Region (SNNPR) of Ethiopia, located 100 km south of the capital city Addis Ababa. There are eight health centers in the district, each serving around 20,000 to 25,000 people in rural areas and 40,000 people in urban areas. The health centers are staffed by nurses and a few midwives, health officers and pharmacy technicians. Most of the nursing staff are trained to a diploma level with only a few having degree level training. There were no hospitals or mental health services in the district at the beginning of the study [17]. The nearest services for people with epilepsy were the nurse-led psychiatric unit in Butajira hospital and a neurological clinic (*“Grarbet”*) run by a non-governmental organization (NGO), both 30 km away from the centre of Sodo district in Butajira town [18, 19]. However, during implementation of the PRIME intervention, a primary hospital opened in the district and a psychiatric nurse was employed by the district and started to provide out-patient services, including for people with epilepsy. A large number of informal providers, mainly faith and traditional healers also provide care in this area [20].

### Study design

Qualitative study nested within the PRIME study treatment cohort of people with epilepsy. The qualitative study also incorporated research questions for a sister project, Emerald (Emerging mental health systems in low- and middle-income countries), which focused on exploration of the health system requirements for integrating MNS care into primary care [21]. Data from both PRIME and Emerald projects are integrated within this paper.

### Sampling

At the time of introduction of the new primary care-based service through PRIME, adults with possible epilepsy who lived in Sodo district were identified by trained community key informants and referred to their local health centre. When the person attended the health centre, they were then assessed by an mhGAP-trained health worker who made the diagnosis of convulsive epilepsy [8]. For 10% of attendees, the diagnosis was verified by a neurologist and all diagnoses were found to be correct. All adults with convulsive epilepsy (*n* = 300) were recruited into a treatment cohort for the PRIME study. This cohort was followed for 12 months and data collection occurred at three time points: at base line, at 6 months and at 12 months. For the qualitative study, people with epilepsy and caregivers participating in the PRIME epilepsy cohort study were purposively sampled. All participants had spent a minimum of six months in the cohort to ensure that they would have had sufficient opportunity to experience treatment integrated into primary care, and spoke Amharic, the official language of the country. Purposive sampling of service users and caregivers considered the differing facilities providing care, the level of engagement of individuals with care and their place of residence (urban vs rural). Both those who engaged with care as part of the PRIME cohort and those who dropped out of care (i.e. were not able to access continuous care, especially those who dropped out after just one visit) were invited to attend by data collectors who visited participants in their home. Sampling continued until theoretical saturation was achieved for service user perspectives on care.

### Data collection

In-depth face-to-face interviews were conducted by TE, a Masters level research assistant with experience of qualitative data collection, in the premises of the health facilities. CH led the development of a topic guide for the PRIME cross-country study. This was then adapted to the Ethiopian context by the Ethiopia PRIME and Emerald teams (co-authors CH, MS, AF and TE). The interview guide explored service user and caregiver perspectives in relation to i) acceptability of treatment received in the task-shared model, including satisfaction with services: ii) barriers to accessing care and how they might be/were overcome; iii) the perceived impact of the new model of care on clinical, functional and economic outcomes of people with epilepsy; v), the process of the new service implementation from the perspective of people with epilepsy and their caregivers; and vi) the pathways through care of people receiving treatment for epilepsy. The interview topic questions can be found in Table 1.

**Table 1:** Interview topic questions

1	I would like to start by asking you a few questions about your recent health problems. In your view, what is this problem?
2	How did you find out that you might have epilepsy?
3	When you first got diagnosed with epilepsy and offered treatment, how was your experience in the health centre?
4	What has been your overall experience with the care you have received for this health problem?
5	What treatments were you offered at the health centre for the epilepsy?
6	For people who have the kind of problem that you describe, health workers might ask whether they had been feeling like giving up on life or even whether they had thought about ending their life.
7	How much did the health worker spend time talking with you about the difficulties you have been facing with epilepsy?
8	How did you find the health worker’s attitude towards you and your condition?
9	How did you feel about the way that your personal information was handled by health staff?
10	After your first appointment, were you given another appointment? Did you attend? [
11	When coming for follow-up appointments, did you see the same health worker each time?
12	How has the care you’ve received in the health centre fitted in with treatment you are receiving from other sources?
13	How confident were you in the ability of the health care workers to help you with your condition?
14	Tell me about your ability to do the things that you need to do in the last few months. How has it changed?
15	Tell me about your economic status over the last few months. How has it changed?
16	Overall, what has been your experience of being diagnosed and treated for epilepsy in the health centre?
17	Is there anything else you would like to tell me about your experience being diagnosed and treated for epilepsy?

The interviews were conducted in Amharic, the official language of the district and audio-taped with permission of the participants. The interviews were transcribed verbatim into Amharic by experienced transcribers and then translated into English by co-author TE.

### Data analysis

Text data was managed using qualitative data analysis software (Open Code 4.02 and TAMSAnalyzer 4.48). A framework analysis approach was employed [22]. Framework analysis takes a relatively more deductive approach to qualitative data analysis than approaches such as grounded theory [23], although does allow for inductive development of the analytic framework and in-depth and systematic analysis of patterns of responses within codes. This approach is particularly suited to studies with narrowly focused objectives and structured topic guides, such as in our study, and has been increasingly applied in the area of health and services research [24]. Initially, co-authors RC and TE familiarized themselves with data by reading through the transcripts. Using a preliminary coding framework derived from the topic guide (informed by consensus amongst co-authors), TE and RC coded two transcripts independently and the coding schemes were compared and a draft coding framework developed. A further two transcripts were coded by TE and RC and consensus regarding the analytic framework was reached with supervision of senior co-author CH. RC coded the remaining transcripts using the coding framework. Once all the data had been coded using the analytical framework, the data were summarized in a matrix output using Microsoft Excel with rows as particpants, columns as codes and ‘cells’ of summarised data. Themes were generated from the data set by reviewing the matrix and making connections within and between participant and codes. This process was influenced both by the topic guide objectives and by new concepts generated inductively from the data. Team meetings facilitated critical exploration of participant responses, discussion of deviant cases and agreement on recurring themes. Illustrative accounts were identified. As a means to increase validity, single counting of the number of participants endorsing particular perspectives was used [25].

### Ethical considerations

Ethical approval for the study was granted by the Institutional Review Board of the College of Health Sciences, Addis Ababa University (Ref. No.084/11/Psy). All service users were clinically stable at the time of interview and written informed consent was obtained prior to participation. Participants were not identified by name in any transcript, report or publication in order to maintain anonymity. Service users and care-givers were given remuneration for transportation costs and time (approximately $2.5).

## Results

A total of 13 service users with epilepsy and 3 caregivers were interviewed. Three of the thirteen service users interviewed had discontinued their care. The three caregivers were not related to the service users who participated in the study and the people they cared for were still engaged with services. The socio-demographic characteristics of these participants and location of the interviews are presented in Table 2. Four out of the thirteen service users were newly diagnosed with epilepsy in the local health facilities after implementation of the new task-shared service; the remaining service users had been diagnosed previously at the general hospital in the neighbouring district.

**Table 2:** Socio-demographic characteristics of service users and caregivers

Characteristics	Service users	Caregivers
Number of participants	13	3
Gender		
Male	11	3
Female	2	0
Age		
< 25 years	2	0
25–34	2	0
35–44	5	0
> 45	4	3
Marital status		
Single	3	0
Married	10	3
Level of Education		
No formal education	7	1
Formal	6	2
Religion		
Orthodox	12	3
Protestant	1	0
Ethnicity		
Gurage	12	3
Oromo	1	0
Area of residence		
Rural	5	2
Urban	8	1
Health care facility attended		
Urban	10	1
Rural	3	2

The analytical framework (Table 3) included three main themes related to experience of integration of epilepsy services in primary health care in rural Ethiopia: i) factors influencing accessibility of services; ii) experience of services and iii) perceived impact of the services. Additional quotations coded under these themes are included in Additional file 1.

**Table 3:** Analytic framework

Accessibility	Experience of services	Impact
Transport Waiting times Medication cost Requiring caregiver assistance Awareness of services	Explanation and advice about epilepsy Medication provision Screening for suicidality and psychosocial support Confidentiality Health workers’ attitude and ability Follow up and Continuity of Care	Clinical impact and Functioning Family Burden

### Accessibility and awareness of the services

#### Accessibility

Over half of the participants (9/16) reported that the proximity of the primary health facilities improved their access to services, with several describing that they were now able to walk to the facility. For a few (3/13) service users the cost of transportation still prevented them from accessing services as often as prescribed.

Several participants (7/16) reported that they had not had to wait long to get the service and compared the health centers favorably to the district hospital and the NGO clinic from the neighboring district which they had previously attended.

*Participant (P). For example, whenever I come here I will be happy if I can get the education and the medication and go back to my house immediately but there can be other patients, so I need to wait with patience.*

*Interviewer (I) . How is it here? For example, did you wait long? Did you go to different health workers?*

*P. It is better here.*

*ID5: person with epilepsy engaged with services*

A few service users (3/13) spoke about the cost of medication as a barrier to care and described needing to borrow money to cover the costs. Others explained (2/13) they have been able to get the medication for free which had improved access.

For some service users (2/13), reliance on caregivers to be brought to appointments was considered an obstacle to accessing care as the caregiver may not be able to always accompany them. Several (5/13) reported that as a consequence of the improvement they experienced with treatment they started being able to come to the health facilities by themselves which eased the burden on their families. Others (3/16) mentioned that services being locally available also made it easier for caregivers to accompany them to the health facility.

*P. There is no one who can possibly take me there. As I told you before my caregiver, the one who come with me today is a daily labourer. Hence he can’t take me there...*

*Everyone will go to work hard, do you understand? He was not willing to come here today at first. But I begged him and he came. This is because he has work and he has children…’*

*ID6: person with epilepsy engaged with services*

#### Awareness of services

Half of the participants (8/16) reported that they came to the health facilities to get the service for the first time after recommendation from neighbors or other members of the community. Some (3/16) explained that they were encouraged to attend the health facility after being told of people with a similar condition which had improved after taking medication.

However, many participants (7/16) reported visiting traditional healers before coming to health facilities, some trying costly treatments out of desperation and several (6/16) interrupted medical treatment to seek these alternative methods at some point after their diagnosis in the health facilities. Several reasons were divulged for temporarily ceasing visiting health services, including advice from others in the community, non-medical conceptualization of the disease, fear the medication may be harmful and the idea that traditional medication and Western medicine should not be mixed. One participant reported using both methods concurrently, visiting holy water sites in the morning and taking medication in the evening.

*P. Many people have finished what they had, their assets, because of me*

*I. What do you mean?*

*P. They have taken me to several places hoping that I will be cured… So then they brought me here last time to try the medication. I had spent a lot of time before I had the illness and before I came to the health facility… ]Traditional medicine. I was taking the traditional medicine for some time and finally I hated it. When I took it, I fell down at the market during the day time. We came here after that.*

*ID 6: person with epilepsy engaged with services*

Some participants (3/16) also described that experiencing improvement on medication received in the health center made them turn their back on alternative medicine and also led them to spread the word about services to their local community thus improving acceptability of services.

*P. Two young people have started the treatment after I explained for them [about the service].*

*I. After you told them?*

*P. Yes...uhh... people use to say she has likift [possessed by evil spirit] because he [her husband] is taking her to let her change her religion [to protestant] but I used to tell them it is not like that….ehh… I told them she will be fine if she takes the medication; she came and she got better. She is on follow up now.*

*ID9: person with epilepsy engaged with services*

### Experience of services

#### Explanation and advice about epilepsy

Although participants named the disease as “azurit” (dizziness) or “yemitl beshita” (falling down unconscious), both terms used to describe epilepsy, there was limited knowledge about the cause, with the majority (10/16) attributing the disease to supernatural causes. Most service users and caregivers (9/16) reported they were given no information by health professionals about the cause of the illness, with a few being told is due to stress (3/16).

*I. What did the health worker tell you your illness is?*

*P. They told me that it is stress. I think it is stress too. Because when I get stressed I will be…he [health worker] said it is stress.*

*ID8: person with epilepsy disengaged from services*

Several service users and caregivers (5/16) reported they were advised by health workers to avoid potential hazards such as staying away from water or fire in case a seizure occurs and to try to minimize stress.

Advice on how to take the medication and the harmful effects of alcohol (10/16) were the most cited instructions participants reported hearing from health professionals. A few (2/13) participants also reported been told to stay abstinent from khat and caffeine. Most service users and care-givers (10/16) reported that health professionals emphasized the need to be adherent to the medication, with a few saying they were told that would lead to cure (3/16).

*They will give me the drug and as I told you they told me as the disease will vanish one day in the future.*

*ID 12: person with epilepsy engaged with services*

Most participants (10/16) reported they were not asked about the use of traditional medicines, with only one saying he was advised not to visit such places and continue his follow up at the clinic. Similarly, very few (2/16) participants reported being informed about potential side effects of medication or were told there would be no side effects if medication was taken according to instructions. However, several service users and care-givers (9/16) reported the occurrence of side effects which were not acted upon by health professionals. In two participants this led to discontinuation of the medication.

*P. Yes. When she takes the tablet with empty stomach, she complains that it has burning feeling and she will stop taking the tablet.*

*I. The tablet?*

*P. Yes, she will interrupt taking the table for someday, for 3 or 5 days*

*I. Why?*

*P. She didn’t say anything. But we ask them if the medication could cause problem, but they explained to us, well that it doesn’t cause any problem.*

*ID 17: caregiver of person with epilepsy engaged with services*

Most participants (9/16) valued being given advice, with several reporting they found it helpful and two suggesting that receiving more information from health professionals would improve their satisfaction with services.

*P….They told me that I have to be free from stress, I should take care when I am around fire…*

*I. So do you think that you are getting advantage when you are following the precautions?*

*P. I took care of everything after that… Instead of worrying……, I was thinking that this makes me better, now I am very fine.*

*I. Can you suggest something that needs improvement for the next time?*

*P. For the future, their advice is nice.*

*ID4: person with epilepsy engaged with services*.

Several participants (4/16) also reported being given psycho-education in their first contact with the health facility but not during subsequent visits.

* … it is good if they would give us some advice and review our health status in addition to giving the medication. They haven’t told us anything. They haven’t asked us how we are doing and about our health status. They just gave us the medication and we went to our home. There may be a problem on this.… It would have been good if they had given us [advice]. …We can protect ourselves. We can tell other patients who haven’t accessed the service here about the illness and other things*

*ID 15: person with epilepsy engaged with services*

#### Medication provision

All participants reported being supplied with antiepileptic medication for treatment of epilepsy (16/16). Often medication was the only intervention that service users reported receiving, a viewpoint reiterated by all caregivers (3/3).

All participants except one reported that medication was always available in the health center, however some participants (2/16) had to collect it every two weeks and were told the medication may expire if given for longer periods due to lack of appropriate storage. The participant that reported a provision problem in the health center was referred to a higher level health facility to obtain the required medication.

#### Health worker attitudes and ability

The vast majority of participants (11/16) described health workers as sympathetic and respectful and none reported feeling discriminated against because of their condition in the health facilities. In contrast, a few participants (2/16) reported feeling rushed when treated in the neighboring district hospital or in the specialist hospital in the capital.

*P. When I came to this health facility, they are treating me properly and when I need tablet, they will give me without any problem.*

*ID 14: person with epilepsy engaged with services*

Several participants (6/16) reported confidence in the health worker’s competence.

However, some participants (4/16) cast their doubts about health workers’ ability to provide the service due to the lack of advice offered.

*I:- In your opinion how is the capacity of the health center staff in treating your illness?*

*P:- They haven’t examined me here. I just took their medication.*

*I:- Eh, So*

*P:- They haven’t asked us other questions*

*ID15: person with epilepsy engaged with services*

#### Confidentiality

All service users interviewed denied having worries about the ability of health professionals to keep their personal information confidential.

#### Screening for suicidality and psychosocial support

The majority of PWE and caregivers (12/16) spoke about the negative impact the illness has had on their mental health, a few (3/13) describing it as tension or sadness and three reporting suicidal thoughts. Some (3/13) of these patients’ conditions improved after starting treatment in the health facilities.

*P…and I was thinking like, why don’t I strangle myself rather than falling down into an abyss and being eaten by a hyena...ehh…or rather than drowned in water…ehh…I said it’s better to strangle myself …and my family was very terrified and they were looking after me…ehh… then, they took me to “Grarbet” hospitall and from there they referred me here.*

*I: but what did you feel when you found out that you are having this illness?*

*P: I felt so bad, I was very dreadful because I felt like the illness might push me into a fire and it might kill me and I still feel dreadful…ehh… even when I am drinking coffee at home I sit far away from the fire…ehh… and I am so happy now that I am getting better and I am currently working as much as I can*

*ID11: person with epilepsy engaged with services*

*… sometimes she hates her life and says she doesn’t want to take the medication and she wants to die…ehh…I always tell her that this is the treatment so she has to take the medication.*

*ID 16: caregiver of person with epilepsy engaged with services*

Most (9/13) service users reported they had been asked by health professionals if they had experienced suicidal ideation. Of these, the vast majority (*n* = 8) reported that they found the question acceptable and helpful, mostly because they valued advice from health professionals as a way to protect themselves from such thinking or to be offered a different medication. The participant that was asked but did not think it was an appropriate question reported fear that asking that question may plant the thought of suicide in a person’s mind. One other service user who was not asked by health professionals said he would not like to be asked as he felt those questions relate to one’s relationship with God.

A few participants (5/16) reported being asked about the difficulties related to their illness and the impact on their lives, and all of them described the experience as beneficial which contributed to their satisfaction with services.

*P. In my opinion or in our tradition it is good if someone asks you how you are doing.*

*… I will be happy if the health professionals ask me.*

*I. Is it only for your happiness?*

*P. It is like that for me.*

*I. Do you like to be asked?*

*P. Mentally … it is a relief.*

*ID9: person with epilepsy engaged with services*

However, the majority (9/16) described not being asked about their wellbeing and perceived this as lacking in service provision. Furthermore, when asked about suggestions for improvement of the services, two participants spoke about wanting further support besides treatment and one asked for help dealing with stress.

*P. If we discussed like this [sitting at] a table, we may tell them what problems we have, if we have some problems, and if we don’t have any problems we can tell them our improvements. So I am saying that I am not happy since they didn’t ask me anything.*

*… it would have been good if they had asked. Sometimes we may have some problems. Every day is not the same.*

*ID 15: person with epilepsy engaged with services*

#### Follow up and continuity of care

All participants except one reported being told and encouraged to return to the health facility for follow up, in most cases on a monthly basis. The one service user not informed about follow up reported this was the sole reason behind discontinuation of treatment.

The majority of service users described seeing different health professionals in their visits to the health centers (10/13). However, around half would have preferred to have seen the same health professional as they reported this would lead to more consistent care and an opportunity for the health professional to take ownership of the care of that patient and know him in detail. However, other participants (3/13) reported they did not mind being treated by different health professionals as the treatment received, medication, was the same. Two participants even expressed preference for different health professionals as this may lead to different kind of advice being provided.

### Perceived impact of the services

#### Clinical impact and functioning

The majority of patients and caregivers (12/16) report that the medication had helped them, either by stopping or reducing the frequency of seizures. Perceived clinical improvement was linked to satisfaction with services in accounts from several participants.

Even among patients who had discontinued treatment, most (2/3) reported the benefits of medication and expressed an intention to return to treatment. However, one participant who had discontinued treatment did so after years of experiencing no improvement; as a result, she turned to religious healing. A few (3/16) participants also reported expectations that epilepsy is a curable disease which acted as an impediment to their satisfaction with services.

*P. Yes I don’t know, but first I went to the health facility thinking that it will help me heal my sickness and start the medicine*

*I. Then when it was not effective what did you suspect*

*P. I just say let me try the Holy Water, I have already taken the medicine for about twenty years, then let me try the Holy Water and I enter in to the Holy Water and now I am healed*

*ID10: person with epilepsy not engaged with services*

Half of the participants (8/16) reported an improvement in their functioning or functioning of the PWE they cared for since they started to attend the health service. This was attributed to both perceived physical as well as psychological improvement, as fear of suffering a seizure was reported as a common impediment to work.

*P. The reason for my life improvement is my health condition improvement.*

*I. Are you working now?*

*P. Yes, I am working.*

*I. Did you work in the previous times?*

*P. I didn’t.…. I feared to work because I thought that I would fall down on wood or other things.*

*I. What about now?*

*P. I am good now.*

*ID14: person with epilepsy engaged with services*

In contrast, some service users (6/13) did not perceive an improvement in their working status either due to poorly controlled disease, the side effects of the medication or other perceived health problems.

*I. How do you see your working ability? Is it improved or not?*

*P. I became tired… I become exhausted. That is it. …Previously, I was working even though I was sick. …I was working at that time even though I was taking the drug. I worked at those times but now I am tired.… I am unable to work. I am tired.*

*ID 12: person with epilepsy engaged with services*

#### Impact on family

Most participants (7/13), and all caregivers (3/3), spoke about the burden the illness had on their families. Several (7/16) described the psychological impact as family members continuously worry for the patients. They also described the economic impact of the disease as some caregivers had to stop working to monitor the service users or to accompany them to health services (7/16). Some service users and caregivers (5/16) reported that services eased family burden as clinical improvement enabled them to return to income generating activities, therefore improving the economic situation of the whole family.

*P. I am always thinking about her while I am working….ehh…I am worried something might happen to her while I am at work and away from her…ehh…I always think about her.*

*I. So, do you think this could create a problem on your job, your farm work?*

*P. Yes, her sickness affects me.*

*P. I can do nothing even though I am worried sick…ehh…I can do nothing…ehh…so, I am not focused on my job.*

*I. Don’t you?*

*P. My attention is with her…eh…she might feel sick or fall down…ehh… we use to eat kocho so I usually stay with her until she finishes baking.*

*ID16: caregiver of person with epilepsy engaged with services.*

However, only one caregiver reported receiving ongoing support and advice from the health professionals, with the other two reporting they are not even told about the appointment date, they are only provided with medication.

## Discussion

This qualitative study is one component of a multi-method evaluation of the impact of primary care-based services for people with MNS disorders in a rural Ethiopian district [26]. In this paper we presented an exploration of the accessibility, experience and perceived impact of the new service model from the perspectives of people with epilepsy and their caregivers.

### Accessibility

During development of the district level MNS care plan in Ethiopia, care was taken to provide services that would be accessible both geographically and culturally [17]. The findings from our study suggest that proximity of the new service decreased the cost of transportation for the majority of service users thus improving access to services. This was despite the existence of a hospital out-patient clinic and a specialist NGO (“Grarbet”) providing epilepsy care in the neighbouring district [19]. These results are in line with stakeholders perceptions pre-implementation, that saving time and money and were benefits of task-sharing MNS services [27].

Medication being provided free of charge to service users in possession of a fee waiver card (provided to the ‘poorest of the poor’) was considered a facilitator for accessing care for some service users. The anti-epileptic medication available is phenobarbital, which is very cheap (up to $1 per month for a standard dosage) [28]. However, even small costs can be prohibitive in this subsistence farming community, as was found for people with severe mental disorders accessing care in the same setting [29]. Community-based health insurance is currently being implemented across Ethiopia which will cover epilepsy care, potentially alleviating this barrier to ongoing access to health services [30].

Our results suggest that several study respondents were made aware of the availability of services by being informed by other members of the community. Firsthand experience of services was in some cases associated with a willingness to promote the services and inform others of the existence of effective biomedical treatment for epilepsy. Studies in the country have found awareness of services to be a strong predictor of health care utilization [31]. Health extension workers have a role in increasing awareness and utilization of health services [32] as well as case finding [33]. Our findings additionally indicate the potential role that peers (people with epilepsy) can have in promoting awareness and acceptability of services. Furthermore, service users and caregivers may also have an important role in countering the high level of stigma against PWE in this setting [34, 35], for example, through social contact interventions which have been shown to reduce stigma in other settings [36].

Despite evidence that community perception of epilepsy as a form of brain-related illness is increasing [10], our results, in keeping with a recent quantitative study [8], show that the majority of PWE and their caregivers sought help from traditional healers at some point after the start of symptoms, often to the detriment of timely access to medical treatment. Faith and traditional healers are a strong community resource in the study area [20] and were identified as a key community group to train in order to enhance collaborative care with services and promote non stigmatizing attitudes in development of the MNS district plan [17]. Our findings suggest a need to improve collaborative care, acknowledging the role traditional healers play in the social and emotional recovery of service users and caregivers, an aspect of care which seemed to be neglected by PHC workers, but also exploring their ability to promote engagement with facility-care and adherence to medication.

### Experience of services

Service users and caregivers described how health worker emphasis was on the provision of medication. This met the expectations of many service users, who considered medication alone to be adequate care. In contrast with previous studies from Ethiopia, there was also no reports of disrespectful or discriminatory practices [37]. However, the finding of high levels of satisfaction with even minimal levels of care must be taken with caution in a setting where no services existed before [38]. Some participants did voice criticism regarding the limited information provided on how to manage their illness and its effects. Participants reported that side effects of medication were rarely acknowledged or dealt with, despite mhGAP training materials enforcing the need to monitor symptoms and side effects and adjust treatment accordingly [12]. Consultation styles were often reported as paternalistic and psycho-education appeared to be given as a one-off exercise at the beginning of treatment with not much reinforcement afterwards. Several service users reported preferring to see multiple clinicians as opposed to always the same one, in hope of receiving varied advice. These unmet needs accord with the need for a change in the orientation of health workers and the health system to people with chronic disorders. As part of the Innovative Care for Chronic Conditions (ICCC) framework, the development of informed activated patients and prepared proactive healthcare team is recommended to improve outcomes for patients with chronic illnesses such as epilepsy [39]. The ICCCF approach requires health workers to work with patients as equal partners in their care, providing them with relevant and accessible information and promoting self-management of their condition. The need for the Ethiopian health system to adapt to the growing burden of people with chronic disorders has been highlighted previously [40, 41].

The vast majority of participants reported being offered ongoing follow up which promoted the acceptability of services. However, continuity of care was limited, with most participants always seeing a different health professional at the PHCs, a possible reflection of high turnover of staff in health centers. One of the core components for providing good chronic illness care is clinical information systems which provide timely useful information about individual patients and populations of patients [39]. Therefore, even if health workers change, there is a need to have a database containing patient specific information not only to promote ongoing good quality individualized care but also to monitor the adequacy of services as a whole in achieving key treatment targets for the population.

A recurrent theme was the heavy emotional burden of epilepsy and its impact on patient’s functioning. A previous study reported that nearly one third of PWE in the same study setting disclosed suicidality in the previous one year [8]. In our study, the majority of service users who were asked about suicidal ideation found the question acceptable and helpful. The requests for further support in dealing with psychological impact from participants highlight the need for development of evidence-based psychosocial interventions acceptable to the target population and to strive towards an integrated chronic care model approach where patients are treated more holistically and all their physical, psychological and social needs are taken into account.

### Impact

The majority of participants reported clinical improvement on medication, which in over half of the participants was associated with ability to return to money generating activities. This was of great importance for most participants, consistent with previous research reporting the impact of treatment on service-users’ lives being described as the most important component of satisfaction with the service [38, 42]. The link between poverty and mental health disorders is well established and our results are in keeping with evidence that interventions for MNS disorders may improve economic status [43]. However a proportion of service users experienced debilitating side effects from the use of phenobarbital, an old anticonvulsant, which has fallen out of favor in clinical practice in many developed nations due to its cognitive and behavioural side effects but continues to be first line in many developing countries due to its low cost [28]. Our study highlights the need for studies to evaluate long-term patient outcomes and the influence of the tolerability of available medications.

Our findings are in keeping with previous studies describing the high level of emotional and financial burden experienced by family caregivers of people with MNS disorders in a rural Ethiopian setting [44] and the potential for locally accessible health care services to diminish the burden considerably [45]. However, most caregivers reported a lack of ongoing practical and emotional support, linking again with the ICCC framework approach that recognises the interconnectedness of patient and family health in this setting [40] . Interventions aimed at engaging families in care and providing ongoing support are needed.

### Strengths and limitations

Our results have to be taken in light of the specific social, economical and cultural characteristics of the study area and may not be generalizable to other settings. Women and caregivers were under-represented in our sample. Nonetheless, our study is one of the few to evaluate impact of task-sharing services for epilepsy care in a LMIC setting from the perspective of service users and caregivers.

## Conclusions

Task-sharing improves the accessibility of services users and care givers to epilepsy care with no concerns expressed about quality of care. Satisfaction with services was closely related to perceived clinical and functional improvement in the majority service users. Service provision focused on medication supply whereas several components of the chronic care model such as promotion of self-management, holistic care and family engagement were highlighted as areas requiring further improvement.

## Additional file

Additional file 1: code queries. (DOC 632 kb)

## Abbreviations

AF: Abebaw Fekadu
CH: Charlotte Hanlon
GM: Girmay Medhin
HIC: High-income country
I: Interviewer
ID: Identifier
LMIC: Low- and middle-income country
mhGAP: Mental health Gap Action Programme
mhGAP-IG: Mental health Gap Action Programme Intervention Guide
MNS disorder: Mental, neurological and substance use disorder
P: Participant
PRIME: Programme for Improving Mental health carE
PWE: Person with epilepsy
RC: Raquel Catalao
RT: Ruth Tsigebrhan
TE: Tigist Eshetu
WHO: World Health Organization
