# Supplementary material for: Implementing integrated services for people with epilepsy in primary care in Ethiopia: a qualitative study
Source: BMC Health Serv Res. 2018 May 21;18:372. doi: 10.1186/s12913-018-3190-y (PMC5963158; doi:10.1186/s12913-018-3190-y)
Supplement: Supplementary file 1 — code queries. (DOC 632 kb) [file 12913_2018_3190_MOESM1_ESM.doc]

**Additional file 1: code queries**

Service users in care

Service users out of care

Care givers

Waiting times

| 1 | 0 |
| --- | --- |
| 2 | I. Are you waiting long? P. No I. Isn’t there? P. No, it is not this much. |
| 3 | 0 |
| 4 | I. Did you wait for a long time to get service?  P. Yes  I. Were you waiting for your turn?  P. Uh, ….after we got the card  I. Uh  P. When we are waiting for our turn…  I. Uh  P. there was long line between…  I. Uh  P. It was like that  I. Was it like that?  P. Yes  I. Uhh  P. But now it is better, here there is no problem  I. There is no problem here  P. Yes  I. Are they not let you wait for a long time?  P. Yes  I. Do you think this one is better?  P. Yes  I. for example how?  P. here the medicine is given ………  I. Uhh  P. here there is no crowed so this one is better. |
| 5 | P. For example, whenever I come here I will be happy if I can get the education and the medication and go back to my house immediately but there can be other service users, so I need to wait with patience.  I. How was it? For example, did you wait long? Did you go to different health workers?  P. It is better here?  I. Uhh  P. It is better here  I. Really?  P. But, the situation in dukuman is grumbling.  I. Why?  P. Waiting our turn  I. Are there many service users?  P. Yes, there are. We had been to waiting long to get card and that irritate us, but things are better here…uhh… I get examined here…eee… they asked me what they had to ask… and they use to tell me the appropriate education…eee… that is good, anyways it is better than the first |
| 6 | I. Eh, then how was the service delivery at that time?  P. The service was like this. We came and then took the medication.  I. Eh, was there a line? Did they ask you to go from one office to the other?  P. Yeah it is common. They would say get a card from there. Is’nt this common?  I. Ehh is it common? |
| 8 | I. Do you have some thing to add?  P. When I came here and took the tablet  I. Ah  P. I didn’t wait here an Hour or half hour or one or two minutes  I. Ah  P. I showed them my card and the recipt, then according to the recipt  I. Ah  P. I told you that they treated me by giving the tablet  I. Ah Ah  P. They are very corporative. Tell them to keep it up  I. Do you have some thing to add?  P. When I came here and took the tablet  I. Ah  P. I didn’t wait here an Hour or half hour or one or two minutes  I. Ah  P. I showed them my card and the recipt, then according to the recipt  I. Ah  P. I told you that they treated me by giving the tablet  I. Ah Ah  P. They are very corporative. Tell them to keep it up |
| 9 | 0 |
| 10 | 0 |
| 11 | I: is that good?  P: yes, but there is a long line starting from there  I: ok  P: there is aline  I: you mean the there are many peoples line up here?  P: yes, bus still I can get the service  I: ok |
| 12 | I. How do you see the health care service delivery when you came to the health facility in the first time?  P. Elaborate it  I. For example, do you wait for long period of time to get the service?  P. Yes,  I. Did you wait for long period of time?  P. Previously, I was waiting many hours but now after I took this ID card, I am getting it with in short period of time. As you know, there is one person called Dawit in that room. I am coming to him in monthly basis and taking the drug sooner after showing this ID card.  I. Therefore, according to what you said now you are not waiting for long period of time to get the drug.  P. Yes, I am not waiting now. I will take as soon as I came.  I. Previously as you told me, you were waiting for long period of time.  P. After I get this ID card, I didn’t wait for long time. I will show them my card and they will give me the drug quickly. |
| 13 | I: How is the health care service here?  P: It is very good. They helped us a lot. They talk to us as soon as we arrived and will prescribe us the medication immediately  I: eh so do you get the medication immediately whenever you want it?  P: Yes we get it immediately.  I: Do you get it immediately?  P: yes as soon as we come, they give us. |
| 14 | 0 |
| 15 | Eh eh, as you have told me before, you have got a treatmet at Butajira “Dikuman’ before you came here  P፡- Yeah  I፡- How was the service?  P፡- It was good  I፡- When you compare it with the service here, which is better?  P፡- I think it is better here. Since there is a lot of patient at “Dikuman”, there is a lot of line there.  I፡- Eh  P፡- You may have also to spend the night there. Such thing is not a problem here.You will get the service immediately.  I፡- So you are saying that it is good because of that?  P፡- Yeah  I. How is their service delivery? I mean their treatment?  P. It is good. We got the service as we come. There is no line. |
| 16 | I. Did you wait long? Or not  P. No, we didn’t wait long. We registered in the morning and they gave us in the afternoon.  I. Did they give you?  P. Yes, we told them we were from rural areas and didn’t have a place to stay and they gave us by considering our situation  ------------------  I.What are the other problems?...waiting long..  P No  I. No |
| 17 | 0 |

Proximity/ transport

| 1 | 0 |
| --- | --- |
| 2 | 0 |
| 3 | I. If a patient has an appointment that person should come based on the given appointment.  P. That person might have a problem for example transport problem…eee… everything might not be perfect every time… yes sometimes…  I. So if there is a problem related to the distance delivering the service in the health centers  P. It is helpful..ehh … it is good and helpful…ehh…it is like that.  I. You have switched the health center due to your own reason however, this health center was closer to your place.  P. Yes  --  I . As you told me you had been receiving the service in Amanuel, Butajira hospitaland health center, here Beke Bishan and also in Kela health center, how do you compare the service provision you are receiving now in kela relative to the other?  P. It is good  I. Really  P. Really  I. Even comparing to Amanuel?  P. Yes of course. Now I am able to get treatment and get back home easily or by paying three birr for transportation. It is very good...ehh... it would be much less if I come here |
| 4 | .I Uhh, As you told me you were having service in “Girar bet” now you are getting here.  P. Uh  I. How do you compare them?  P. This one is near I can come on foot.  I. Uh  P. It minimizes cost.  I. Uh  P. Since it is near………  I. Our origin is country side and this Epilepsy it can be treated like other sickness or do they think that it is another thing?  P. … family this place is remote area  I. Yes  P. There is no infrastructure like road…Uhh… So they do not think that it is curable.  I. how is the diagnosis in the health station/center?  P. Uh  I. how did you get/see the ”kella”?  P. it is…………  I. Is it better?  P. It is better  I. Is it better?  P. Yes  I. Uh, how?  P. now the place is near  I. It is the same ,how do you compare the access with grar ber?  P. this one is good. |
| 5 | P. I paid transportation fee for trips for my son and me and paid today to…but it is okay …you have to go through a lot to get your health  I. It is true  P. You have to go through a lot  ---------------------------  P. Yes, it does. I have been taking the medication for the last one year and six months, even though there were some interruptions.  I. How did it help you?  P. rarely due to different reasons such as transportation problem, card related….eee…now I have transportation access, there is bajaji in the nearby  I. …eee…  P. I can go to butajira if the road is not muddy  P. I sometimes lose my medication with other things  I. …ee…  P. Wherever I go I should take my medication with me  I. …eee...  P. If I drop my medication there is no problem now because there is transportation.  I. How about transportation or time or money?  P. We have to overcome that problem since it is for my health whether we browed by giving our cloth as collateral but there should be someone with me ..  I. to come here  P. Yes |
| 6 | P. It is a problem of money. I don’t need money for transportation.  I. You don’t have this problem because you come by foot?  P. Yeah, thanks to God I don’t have such problem. I will come and go. This is just for me  I. Do you think that this might be a problem for others?  P. I just feel sorry for them.  I. But do you think that this might be a reason for others? Do you think that patients might not come here because of problem of transportation?  P. Yeah, how a person could come if he didn’t have money for transportation  I. Eh  P. How could they come from those places for example some patients come from far places.  I. Eh  P. Those who come from far places, will they get a transportatiom from here?  I. Eh  P. They might be ill while they are coming. |
| 8 | It is because I had a work to do. I don’t have other reason.  I. Really  P. Really  I. How about money  P. Money….  I. I mean money for transportation  P. Yeah, I have this problem |
| 9 | I. So were you unable to come because your work place was far?  P. Yes, it was  I. Due to your work?  P. Yes |
| 10 | 0 |
| 11 | I: what about other things like… financial problem, stigma, lack of time …do you think these things might inhibit peoples from coming on their appointment date?  P: concerning money?  I: for example, lack of money for transportation?  P: I didn’t experience anything  --------  I: so, how do you assess the treatment here as compared to the “Dikuman”?  P: ok  I: how do you see it?  P: this is very nice, its very near to me and I can come anytime I want |
| 12 | I. But now you are coming to this facility to take the drug by your self. What is the reason for this? Is it due to its nearness or are you getting better?  P.It is near and also I am getting better thanks to God.  ------  I. Did you unable to go to Butajira due to financial problem?  P. No, I didn’t. If he has no money in his hand, he will borrow from somebody else and bring the drug. He was always responsible to bring the drug in monthly basis. He took the card and brings the drug for me.  I. Is he always brings the drug?  P. Yes, he always brings the drug from Butajira.  I. Why he brings the drug for you? Why not you yourself?  P. Both of us went to the health facility to take the drug at one time but the health workers know our financial problem and told my husband as he is my care giver and able to take the drug in monthly basis without taking me to the health facility.  I. Was there problem in getting money even for transportation?  P.Yes, there was |
| 13 | I: Eh so it is because you had that experience  P: Yes, when I took her, they confirmed it is for sure that illness. Then they wrote me a letter and transferred her to here. They wrote me a referral to here since I told them that I can’t afford the transportation and other costs.  -----------  I: As you told me before you encountered a problem in dikuman due to transportation.  P: Yes  I: Do you think that the burden is reduced for you since you transferred to here?  P: Yes, I think [it reduces it] very much.  I: eh so it is easy for you to bring her here?  P: Yes, it is very much easier. |
| 14 | P. If we have challenge, we will pass that challenge.  I. How?  P. If I have no money…..  I. Don’t you come?  P. I will not come if I have no money but I will come when I have money. The health professionals will ask me if I didn’t come.  I. If a person has no money, he may not come to the health facility to take his drug and as a result he will become sick. How do you see this?  P. I see it as my fault.  I. How?  P. Because I am unable to come  I. It is because you don’t have money  P. Yes, it is due to financial problem but it shouldn’t be a reason. |
| 15 | 0 |
| 16 | I. Do you have to pay for transportation when you come here?  P. No, we don’t.  I. So, there is no problem  P. It takes one hour…ehh…we live up there, there is no problem.  I. So, you don’t have any problem to come here  P. No, we don’t; there is a problem if we go Addis Ababa.  I. There is a problem if it is Addis Ababa..  P. Yes  I. How do you compare the service in Amanuel hospital and the service here?  P. It is difficult to go to Addis Ababa: transportation…okay…when we go there the transportation may not be available on the appointment day…ehh… at that time there was only one car and that car might not have enough seat…ehh…because of this we could miss the appointment day…okay…we might arrive in the evening…okay…Addis Ababa… uhh…. at that time a place to stay was also a problem…ehh…. it was difficult because of those things … ehh…but when we come here it will take only 2hours …ehh…this is more accessible. |
| 17 | P. Let God bless them, I am so happy, so happy. The major reason I am happy in one hand is, going to Addis Ababa from sodo was becoming difficult for me.  I.Ehh…. How about now?  P. The distance, plus there was no transport service at that time  I.Ehhh  P. After we start attending here, this year…  I.Ehhh  P. We come here every month and collect the tablets. We even walk to reach here. I am so happy  P. Let God bless them, I am so happy, so happy. The major reason I am happy in one hand is, going to Addis Ababa from sodo was becoming difficult for me.  I.Ehh…. How about now?  P. The distance, plus there was no transport service at that time  I.Ehhh  P. After we start attending here, this year…  I.Ehhh  P. We come here every month and collect the tablets. We even walk to reach here. I am so happy  P. Now we are transfer to here. It is near, it only take us 1:30 to reach here. We have transport access.  I. What problem did you face to come here?  P. Here?  I. Ehhh  P. Here gereno silassie?  I. Ehh  P. She feels some tiresome, since she has to walk for 1 and half hour to reach here. But, I am feeling good. Actually, I had surgical operation last time due to renal failure which hindered me from urinating. |

Cost of Medication

| 1 | 0 |
| --- | --- |
| 2 | I. Are you buying the medication?  P. Yes, I am buying here.  I. Previously, your parents were buying medication for you; now you told me that you are selling plants to earn money…okay… do you think that you can buy your medication you’re your earnings?  P. I don’t have a sustainable income… |
| 3 | P. No, we are not...errr... no we are buying  I. You are buying, right?  P. Yes  I. Are you buying all these by yourself?  P. Yes, I am  I. but in previous times someone was buying for you?  P. My father was but now he passed away  I. but now you are buying it by yourself because you are able to do it.  P. Yes...ehh... there is nothing that can be done  I. Ehhh ..ehhh...so...as you have told me that there is medication expense.... so do you think you are moving independently because of the the treatment you hare recieving?  P. I am enjoying it. |
| 4 | I. Do you think this one is better?  P. Yes  I. for example how?  P. here the medicine is given ……… |
| 5 | P. What about the medication?  I. For example, how is the provision? Are you receiving the medication on time?  P. Yes, since I came here I never wait to get the medication.  I. isn’t there…  P. There is no… when I come here …eee…I will pay here and go there and take my medication and leave |
| 6 | P.I don’t have a work here. I don’t manage my life by myself. }Instead the people in my neighbourhood gave me what they have. They are good, may god bless them.  Some invite me lunch and some cover other things. The medication needs food alot. Do you understand? You have to eat a food inorder to take the medication. If you have a shortage of food, you don’t say it is a cliff, it is fire, a car is coming or other thing. Think about it. These things are the one that touches me. That’s it. |
| 8 | 0 |
| 9 | P. For example there is one girl ..ehh… when the price of the medication increases  I. Price  P. When the price increases from 5 birr to 33 birr/three months, it became difficult for her family and they want to stop her. but I told them how worsen it could be if they do that and we brought her together. She is living in Timuga and she is mute …ehh … she is unable to speak … ehh .. money was a problem for her family to come here per month.  I. Was it difficult for them?  P. Yes, it was a sudden change for them … ehh … they are near to the place I am living in . They say they don’t have a problem to buy the medication however in my understanding they had the difficulty.  I. So do you think money can be one of the problems?  P. Yes…ehh…ehh… it was like edir monthly fee for them.  I. Was it difficult for them?  P. Yes, I do observe such things. |
| 10 | I. If it was good… yes…. Did you get the medicine as you wish … yes … every time….. yes .. what are the other problems?  P. There was no other problem  I. Was there any financial problem?  P. finance, .. uhh.. Whenever I have no money  I. What do you mean whenever I have no money?  P. Yes whenever I have no money, I have borrow from other people to go to the health facility |
| 11 | 0 |
| 12 | I. What problems do you have to take the drug every month? For example, time, money and other challenges that you faced.  P. Currently, I am not paying money but previously I took the drug by paying money. Somebody told my husband as it is possible to take the drug freely and I got an ID card for that service and currently I am taking the drug freely.  I. Is that ID used to take the drug freely?  P. Yes, it is used to take the drug freely. Somebody told my husband about this because this person knows as I have no work and sometime I may not have money and my husband may not always be with me. That person told him to get this card and to use the service freely using this card.  I. Before you get this free service paper, were there such kinds of problems to buy the drug?  P. Yes, there were. But the problem was I didn’t remember the amount of money that I paid. I forgot it. I have recall problem. I saw you today but I may forget you tomorrow. I have such kind of problem which is mainly related with the tension that I have in my mind.  I. Ok  P. It was not free before not only here but also in Butajira. I bought the drug in Butajira |
| 13 | We don’t pay for the medication as well. It is free.  I: Really? P: Yes  I: How can they gave you for free?  P: They just gave us. We don’t have anything. I finished all my previous properties for treatment and other things.  I: Due to her treatment? P: Yes  I: Did you expend a lot?  P: Yes, I spent a lot. I lost my car and my other properties. I had cattle.  I: Did you have a car?  P: Yes, I had a car. I sold all my cars.  I: I mean what problems did you encountered to bring her here from your side or from your house; not related to the service? For example it could be financial problem or not having a time or other things  P: Currently I don’t have any problem. Thanks to God!..eh.. In the past I suffered a lot since I didn’t have a money to pay for the medication and other things.  I: Since you used to pay for those things, right?  P: Yes…..I: Okay |
| 14 | I. Did doctors consult you when you come for treatment?  P. When I came they will tell me to go to the casher to pay money |
| 15 | P፡- Time is yours. The time is limited up to Sunday. He will not asked to pay 1000. It is some money. If he works one day and pay it, it is good. Thanks to God.  I፡- Eh, so you are saying that this couldn’t be a reason?  P፡- Yeah  I፡- Never |
| 16 | 0 |
| 17 | 0 |

Requiring carer to attend

| 1 | I. for example, the expense of the medication or ….it because you don’t have anyone who can help you to come here…?  P. No, it is not like that.  I. Or it is because the service delivery was not good when you came here last time?  P. The service delivery was good, and also it is not because I didn’t have anyone who can help me to come here. The reason is I didn’t have information that you were here. |
| --- | --- |
| 2 | 0 |
| 3 | I. How are you taking the medication, are you taking it yourself or someone will help you reminder?  P. Now I came with my child,  I. What about in other times, is there any time you come alone?  P. Yes No one will come, I came alone  I. So you came alone without the support of anyone?  P. Yes  I. Do you need someone to remind you about your appointment?  P. No I have a recording card |
| 4 | I. Uhh,uhh,uhh are you coming alone to take medicine?  P. Yes  I. Are you taking by yourself?  P. Yes |
| 5 | I. How about transportation or time or money?  P. We have to overcome that problem since it is for my health whether we browed by giving our cloth as collateral but there should be someone with me ..  I. to come here  P. Yes  I. So, if that someone can’t make it  P. If she can’t make it, with whom can I come? Last time I came with my son and go back with nothing. that is one problem.  I. So, if she doesn’t have time  P. Yes, it is one problem  I. there is a problem  P. I came here to tell them last time…eee… I came here on my appointment day with my son but they told me to come with my wife and send me back.  I. Did they say you couldn’t get the service unless you come with your wife?  P. Yes, they asked me how old my son is, and I told them he is fourteen, then they told me it is impossible and to come with her. I asked him that I lost my card and how I can come on my appointment day, he told me to bring her because she is the one who knows about my sickness. At that time my head...  I. Did you feel sick?  P. I said okay, because if there is stress…eee… that is something….  I. Then, did you bring her?  P. Yes, I did today  I. Today  ee… I almost decided to quit because of last times circumstance …eee…I had a thought to accept any consequence …uhh…I bring my 14 years old son…uhh…my wife was sick…uhh…because of that I bring my son instead of her but the health workers didn’t tell me the way how I could get my medication, my son has a better understanding. I felt bad about it….eee… I was not in a good condition after I return home.  I. What obstacles are there which can hinder a person to attend his/her follow-up?  P. For example, only my wife is here for me but if she feels sick…eee..or if some other social problems occurred…eee…. To come with me…eee…she is a mother…eee… if there are those things we may not able to come on my appointment day |
| 6 | P. There is no one who can possibly take me there. As I told you before my caregiver, the one who come with me today is a daily labour. Hence he can’t take me there.  I. Eh  P. Everyone will go to work hard, understood? He was not willing to come here today at first. But I begged him and he come. This is because he has a work and he has childrens, understood?  I. Eh. Yeah  P. So that is why  I. Then how is he taking care of you?  P. He will come to my home and will do everything I need.  I. Eh eh, when you come to the health center to get the medication, did you come alone or with other person?  P. It is with other person  I. Always  P. With my caregiver  I. Always  P. Yeah |
| 8 | 0 |
| 9 | I. Did you come here alone?  P. Yes, I came alone  I. Nobody was with you?  P. Yes  I. So are you coming for your follow up alone?  P. Yes; I used to go with someone in sidamo  I. Did you?  P. Yes  I. Is it improving now?  P. Yes |
| 10 | I. Really, but your father was accompanying you to the health center, wasn’t it?  P. Yes he was going with me  I. Then what did your father say when they reduce the amount  P. Nothing, he just say it is reduced take it .. , yes uhh… he did not tell about it  I. he said it is reduced and take it, which was the only thing you were informed?  P. They did not tell me the result, and also about my status, the status of your sickness? |
| 11 | 0 |
| 12 | 0 |
| 13 | P: Yes, she came here independently the day before yesterday and took the medication. In the past she used to come with other individual. But now she can come and take the medication by herself. |
| 14 | P. At the beginning, I came with my caregiver.  I. Are you coming with your care giver?  P. Yes  I. What about now? Are you able to come alone?  P. Yes, I am coming alone to take the drug  I. How do you see yourself? Are you able to come alone?  P. Yes  I. So, you don’t need care giver |
| 15 | I፡- When you came to the health center, you used to come with other person right?  P፡- In the past  I፡- Yeah in the past  P፡- Yeah I couldn’t trust myself to go alone.  I፡- Do you think that this could bring a change to your life or to your relatives’ life? Since they have stopped accompanying you to the health center, do you think that they can do their work and this will make change to their life?  P፡- To whom, to me or to them  I፡- What difference could it make if they accompany you or not?  P፡- They can do some works and other many things until I comeback from the health center.  I፡- Yeah!  P፡- They will wait me doing their works.  I፡- So are you saying that it is good since they have stopped accompanying you to the health center?  P፡- Yeah What Can I do for my lord |
| 16 | I. Did you face any difficulties bringing your wife to the health facility?  P. To bring her to the health facility, we didn’t have other people to help, our children weren’t grown up  I. At that time  P. Yes…ehh..There was no one who can take care of the cattle’s, that was the problem…  I. Was it difficult?  P. Yes  I. So, what did you do?  P. So, I took her by letting my neighbors to take care of the cattle, her life was more important. I. What about on the appointment day to take the medication?...ehh… what are the reasons which can hinder the service users to come here on their appointment day?  P. Difficulties, if she gives birth…ehhh…she might not able to come or I might not able to come if I am sick. It is only if we face such problems…keeping the appointment date is compulsory.  P. So, we will come by keeping our appointment date.  I. Every two weeks…yes….have you been coming here with your wife or alone?  P. Alone  I. Why are you coming here alone?...ehh…why are you coming?  P. By thinking that she might get tired or she might get sick on her way?  I. Do you think like that?  P. Yes  I. Did you ever try to send her to this health facility and did she ever experience failing down on her way?  P. I don’t trust..  I. Is it a fear?  P. Yes..ehh…the road has ups and downs…ehh…yes  I. What about on the appointment day to take the medication?...ehh… what are the reasons which can hinder the service users to come here on their appointment day?  P. Difficulties, if she gives birth…ehhh…she might not able to come or I might not able to come if I am sick. It is only if we face such problems…keeping the appointment date is compulsory. |
| 17 | 0 |

Community awareness of services

| 1 | I got sick again another day…at that time they (some people) told me to go to Dikuman (Girare bet Hospital) [specialist facility for people with neurological difficulties in Butajira town]….you came here while I was thinking to go there, and people advised me to come to Kela health centre instead of going to Dikuman, and they brought me here and let God help them they gave me medication and I took that medication…  P. The service delivery was good, and also it is not because I didn’t have anyone who can help me to come here. The reason is I didn’t have information that you were here.  I. Don’t you know about the availability of mental health service delivery here?  P. People told me that it is better to go to the health centre when the foreigners come and it will be announced when they come. I said okay and I have been waiting.  I. Don’t you know the availability of the service here, they have been delivering the service?  P. All the time?  I. Yes  P. I don’t know |
| --- | --- |
| 2 | 0 |
| 3 | 0 |
| 4 | I. In your surrounding what did they call it? Or what is the cause of the sickness?  P….It is known that it is Epilepsy  I. What is the cause of Epilepsy?  P. I do not see or do not know that this sickness get enough attention. |
| 5 | P. I failed down two times consecutively, and I asked why it happened? And people said to me it was anemia (‘Deme-manes’). But, since the time I start losing my conscious completely they told me it is epilepsy. They also told me the presence of others with similar problem and their experience of taking medication, the possibility to get better by taking medication, and due to their advice I went to Dukuman.  I. Did you find out it is epilepsy before/ after you came to the health facility?  P. Previously, I thought it was anemia …eee…. but then when I fall down again and again they told me that it is epilepsy. They also told me ’’this this people have the same problem and they are using medication and you need to use too the medication’’. I have a sister…eee… I do not have anyone else, she lives in butajira she helped me to take the medication from ’’ Dukuman’’  I. Did she tell you?  P. Yes, she told me to keep my follow up since she is urban resident (she knows better about treatment because she is an urban resident)…uhh…and I have been doing it ….uhh… both health workers here and there told me how alcohols oppose with the medication. |
| 6 | P. It was found out that it is epilepsy there.  I. Did you found out there?  P. Yeah, I had a seizure again after I came here.  I. Eh  P. Then my friend’s caregiver, the one who brought me today, brought me here  I. He brought you here?  P. Yeah  I. Eh eh  P. After that I am taking the medication properly.  i. The people who used to give you the traditional medicine, did they told you not to go to a health facility?  P. Eh  I. Did they tell you not to go to the health facility?  P. No, they don’t told me  I. Didn’t they tell you?  P. They dont have a knowledge about the treatment at health facilities |
| 8 | 0 |
| 9 | I. Okay, since the service starts here. P. The health professionals told me it has a medication...ee....even there was sick young lady in our community...eee... two young people have started the treatment after I explained for them.  I. After you told them?  P. Yes...uhh... people use to say she has likift he (me) is taking her to let her change her religion (to protestant) but I used to tell them it is not like that….ehh… I told them she will be fine if she takes the medication; she came and she got better. She is on follow up now.  P. I came here when people tells me the availability of the service |
| 10 | 0 |
| 11 | 0 |
| 12 | I.Who told you as the service is available in Grarbet Bet Le Dikuman?  P.Every body went to that area for Epilepcy disease. It is in Butajira.It is called Butajira Dikuman? I think it is a place for treating epilepcy |
| 13 | I: What do you suspect it is when you took her there?  P: When she started to scream and bite her tongue, I suspect and took her.  I: Have you heard about such kind of illness before?  P: Yes I knew about the illness before. When I was at Sidama, I used to live in southern region, we saw together in ziway when a woman had a seizure….eh…Then when she had a seizure in our house, I thought the illness could be like that and took her immediately. |
| 14 | I. When did you know it as an epileptic disease? Is it before you came to health facility or after you came to this health facility?  P. It was known as an epileptic disease when I first fell down and people told me to go to health facility.  I. Who told you to go to health facility?  P. They are our neighbours  I. Are they your neighbours?  P. Yes  I. What did they said the name of the disease?  P. There is similar patient in our neighbor  I. Is there similar patient in your neighbour?  P. Yes, there is a patient who became healthy from this disease.  I. Is there patient who recovered from the disease?  P. Yes  I. By what way did the patient recovered from the disease?  P. Using tablet  I. Do you thinking as this disease can be cured by medical treatment?  P. Yes, I am well now. |
| 15 | I. But how did you know that you have epilepsy? How did you know?  P. I was unconscious when I had a seizure and fell down. After that I went to Butajira hospital and got assessed there and have started medication. Occasionally  I. Eh  P. When I had a seizure and fell down, my neighbors and families are the one who knew it.  I. What did they say it is?  P. They said it is Epilepsy  I. Did they say this immediately?  P. Yeah  I. Before you went to health facility?  P. Yeah, I don’t give it attention when I had the first seizure. But it happened again.  I. Eh  P. Then they told me what this illness is. They told me to go to the health center |
| 16 | There were other people who had similar sickness…ehh…she lose her conscious and has saliva drop; everyone who observe her condition says it is epilepsy.  I. Did someone tell you?  P. Yes…uhh… we did not know about the treatment and we just said it was epilepsy after we observe how she was sick…ehh…I went to different places but she couldn’t get better … ehh … so I went to Addis Ababa based on other people’s recommendation.  I. Did they tell you the availability of the service in Addis Ababa?  P. Yes..ehh..They told me to go to the health facilities rather than spending my money by going other places like kalcha…. |
| 17 | P. After I recognize her problem, she has younger sister who lives in Addis Ababa around the village called GORE…  I.Eh  P. Around Gore, there is a place called “SIBO”. Her youngest sister lives there. Thanks to her, she went to Amanuel hospital and coordinates with the doctors and she made her to start taking the medication. |

Competition with traditional healers

| 1 | I. What other services did you receive (other than modern treatment)?  P. Nothing  I. Didn’t you go to the holy water?  P. No, I didn’t…uhh…I didn’t go anywhere else.  I. Did you come here directly?  P. Yes, I did. |
| --- | --- |
| 2 | I. What did you decide to do after you find out it is epilepsy?...eee….what did you decide to do?  P. To get health professionals advice/consultation.  I. Did you decide to receive health professionals’ advice?  P. Yes  I. Didn’t you go to the holy water before you came to the health facility?  P. I went to the holy water after  I. So, you went to the health facility first?  P. Yes  I used to have a follow up in Butajira Grarbet Hospital  I. What did they say?  P. They didn’t say anything to me.  I. What did they say that your sickness is?  P. They told me it is epilepsy and advised me to take the medication without interruptionbut I interrupted it once…uhh…and went to the holy water.  I. What do you or people who had been there when your sickness starts think is the cause of the problem?  P. They are thinking nothing….just it is epilepsy and they want me to go to the holy water…  ----  Did the health professionals explain for your parents about epilepsy and its treatment, as you told us your sickness starts when you were grade one?  P. Yes  I. Did you start the treatment since that time?  P. Yes  I. Okay  P. but I dropped out at grade six.  I. Why?  P. It didn’t bring any change, then the holy water …eee…they told me to try the holy water and they took me there.  I. Did you go to the holy water?  P. Yes  I. Then how was the holy water?  P. the holy water, I went to St. Marry church in Butajira and to Mojo too [town, about **km away from Butajira]…okay….I stayed there for two weeks.  I. At Mojo?  P. Yes  I. Didn’t you see any change?  P. No, I didn’t see any change and I quitted and came home.  I. As you told me, you went to the holy water by quitting the medication…okay… did the people in the holy water tell you to quit the medication?  P. They simply baptize people  I. What did they say about the medication?  P. They didn’t say anything.  I. Did they tell you to stop coming to the health facility?  P. No, they didn’t.  I. What did the health professionals say about the holy water….eee…how the health professionals perceive your visit to the holy water?  P. Nothing; I didn’t even discuss with them.  I. How these different treatments (the medication and the holy water) complement one another?  P. I didn’t see any change, it is up to God. |
| 3 | P. when the severe headache was healed again I went to the traditional medicine, there was many other things done, but the traditional medicine, but finally you may wonder after everything was done in the traditional medicine …uhhh… with many tiresome efforts even I was taken to Entot in Addis Ababa to someone who was practicing a traditional medicine, the person gave me something through my nose (nostrils) …uhhh… that was very hurtful who aggravated everything  I. Was it the Holy Water at Entot?  P. No it was not; that was some kind of medicine which was hurting, that was scorching even my head …uhhh… finally it exposed me even to lung disease  P. I went to the holy water, in addition I took this burning...  I. Other traditional medicine  P. I took it through my nose unknowingly  I. Other traditional medicine  P. Yes, I have felt dizziness immediately when I took it.  I. Was it the holy water place?  P. It was in the holy water place, he was a monk ... he had two big people and he ordered those people and then picked me up and put me in the holy water. It had happen long time ago in 1986 E.C (Ethiopian calendar).  I. Do you think this traditional medicine can be a treatment for this sickness (epilepsy)?  P. I don't think so?  I. Really?  P. I didn't try it till now and also I don't think it is a treatment ...ehh... don't tell me to think about it unless you want me to die. I am not going to think about traditional medicine while this service is available here.  I. Did the health professionals advice you not to take traditional medicine?  P. No, I never hear them saying that  I. Didn't they say that?  P. I never hear that  I. Did people in the holy water tell you not to go to the health facility?  P. No, they didn't |
| 4 | P. This sickness … or they think that it is hereditary…uhh… Uhh  P. But I caught this sickness accidentally… Uhh… It is like this…. I do not have anything to say  I .What did they do when you are sick?  P. Nothing but they gave me traditional medicine.  I. Traditional medicine  P. it is drinkable traditional medicine  I. Plant, something else  P. Yes  I. Ok  P. But it could not cure me  I. Ok  P. After that I went to hospital  I. But did you use traditional medicine or holy water?  P. Urr, they told me to start but I said I won’t start.  I. Is it only traditional medicine?  P. I started traditional medicine for one or two days and I stopped it.  I. Uhh, Are you thinking that these traditional medicine and holy water are not medicine?  P. It is not medicine.  I. Are you thinking like that?  P. Yes |
| 5 | P. I got sick while I was receiving the service from Butajira (Dukuman), at that time I went to Addis Ababa and I tried the holy water but I couldn’t get better; I had been receiving the service here in Butajira after I came back but I lost my card I didn’t know where I lose it while I was asking the solution they told me the availability of the service in Kela.  --  I. What do you think/believe?  P. Nothing, I simply ask God how it happens to me…ee… when I ask nobody in my family had such type of problem …then why… just it happens…. When I discuss with my families they told to go to the holy water and medical follow up.  --  I. Did you ever practice traditional medicine or holy water?  P. Previously  I. Was it before you start the treatment?  P. Yes, it was at the beginning…ee.. Yes, some people told me to try the holy water because we thought it was anemia.  I. How was it?  P. I tried but there was no any change.  I. Have you ever go after you started the treatment?  P. This is better even though we can’t be sure.  I. So, Haven’t you be there after you started the medication?  P. No, I haven’t, because my families use to tell me how the holy water and the medication opposes. |
| 6 | P. Many people have finished what they had, their assets, because of me  I. What do you mean?  P. They have taken me to several places hoping that I will be cured.  I. Eh  P. So then they brought me here last time to try the medication. P. I had spent a lot of time before I had the illness and before I go to the health facility  I. Eh  P. By using medication, I mean other kind of medication  P. Traditional medication (ye Habesha)  P. Now for example I was taking the medicine for some time and finally I hated it. When I took it, I fall down at the market on a day time. We come here after that  P. It is just epilepsy.  I. Ya epilepsy is epilepsy. What I mean is did they told you that it is not going to be cured and other things?  P. Some people said it will be cured others said it wont be cured.  I. Eh  P. Let me tell you. For example now while I am taking this medication, some people recommended me to go to a holy water, others suggested other many things  I. Okay, when you have an appointment here, when you or other person have an appointment here, what are the factors that will made you/him to cancel the appointement? What are the reasons that will make people not to come on their appointment day?  P. Yeah  I. What are the reasons?  P. Some people say go others say don’t go  I. What do you mean?  P. Some people………..  I. Eh, do you mean other people?  P. Yeah  I. Eh  P. For example it has been a month since I took my medication from here. Some people may say don’t go, what did the medication did for you?  I. Did they say what did it help you?  P. Some people say that what the medication will help you. |
| 8 | I. Okay, Did you find out that your illness is epilepsy when you came here or you first knew it at your home?  P. When they said like that, I requested them that I don’t want to go any where. It is God’s gift and he will take it away from me. I didn’t buy it by money or no one bought it to me  I. Ah  P. An enemy can’t give this. It is a gift of God. I won’t go to a wizard. I told them that if it is Gods gift, I may use medication depending on the situation.  I. Okay  P. I told them that I won’t go to other places. When we asked what the illness is, they said it is epilepsy. I came here and they confirmed it and I started the medication.  ---  P. They knew it at home too.  I. Have they known it in the past?  P. Ah  I. Who knew it? How could you know it?  P. Other person, when other person gots ill  I. Ah  P. When my families told them that I had a seizure and other things, they told my families to take me to traditional medicine, to a wizard  --  I. What about private…traditional medicine (herbs).  P. Nothing  I. What about holy water  P. I didn’t drink holy water. They told me that this is the only treatment  I. Ah  P. I had started taking the tablets here |
| 9 | I. Okay, when you first got diagnosed with epilepsy and offered treatment, how was the service delivery?  P. At the beginning it was in Yirgalem Hospital.  I. Did you the treatment in Yirgalem?  P. Yes; and then I discontinued ...ee... I felt that the medication might be bad if it is adapted, this was also the comments from others....ee...I went to the holy water but it couldn't made me better...ee... I will be get better and start working and then...ehhh... I will be get sick .... they advised me that I will not be able to continue my education if I discontinue the medication.  I. This sickness called Azurit or in English it is called Epilepsy...ehh.. so how you felt when you find out you have this sickness?  P. At that time...ehh...I thought it was likift ....ehh... I believed in that and I went to the holy water and to other different places.  I. Did you go to the holy water?  P. Yes...uhh... there was no change but to rest...  I. How has the care you are receiving in the health center fitted in with treatment you have received from other sources (holy water, traditional treatment..)?  P. The traditional is not…  I. Have you ever go to the traditional healer?  P. Yes  I. How about the holy water?  P. Yes, I went to the traditional healer and to the holy water at the beginning. I believed I could be cured. But I experienced something which makes me feel inferior and ashamed still now.  I. Traditionally  P. Traditionally, my family did it…ehh…I believed in it at that time but when the time passes it was not ….  I. There was no change?  P. No, it didn’t have any relation and I recognized that since I received an advice from the nurse who were doing dressing in Yirgalem Hospital.  I. Did you think like that?  P. Yes  I. Did the traditional healers tell you not to go to the health facility?  P. Yes  I. Did they tell you not to go?  P. Yes  I. What did they say?  P. The sickness will not be cured if I start the medication  I. Did they say like that?  P. Yes, using the praying and the holy water but not taking the medication.  I. Is the holy water and the traditional treatment the same?  P. It is not the same.  I. Is the holy water different?  P. Yes  I. Did both of them tell you not to go?  P. Yes, if I took it repeatedly…ehh… they told me that it will not be cured because the medication is not good …ehh… on the other hand there were family members who were telling me about a person who was cured after his visit the health facility …ehh… then after it was mu decision to choose. |
| 10 | P. Yes I don’t know, but first I went to the health facility thinking that it will help me heal my sickness and start the medicine  I. Then when it was not effective what did you suspect  P. I just say let me try the Holy Water, I have already took the medicine for about twenty years, then let me try the Holy Water and I enter in to the Holy Water and now I am healed  I. So do you think the Holy Water is better?  P. Yes I think the Holy Water is better, that is what I think  I. So you think the Holy Water is enough and do you have the plan to return into the health facility and start all over  P. I don’t know that but I don’t think I will start that again  P. I was taking the medicine throughout the month and I finished it, .. uhhh….. but I was felling at the same time, while the medicine was finished I went to the Holy water, I was better to a little extent, now I am better  I. So it is the holy water, uhh yes, when was the last time you fell  P. I am okay until know  I. Are you okay for the last one month after you stop the medicine and start the holy water?  P. Yes, it is now one month, since I start the holy water and I am healed from the epilepsy, now I am not felling  I. do you mean this happened after you start the holy water, How long is that  P. It is now about seven days  I. That is the duration you stayed at the Holy water place, but when was that happened  P. That was before two months  I. but according to you, do you think it is conflicting to use both the howl water and the Medication simultaneously  P. both of them  I. Yes is it not possible to use both at the same time?  P. Yes! It is impossible  I. Why?  P. Do you mean to use them together?  I. Yes, is not possible  P. Yes, it is not possible to use them together!  I. Why?  P. I don’t know  I. Who told you?  P. I was told when I was using the holy water |
| 11 | I: have you ever went to holly water and the like?  P: no, I never went there  I: what about herbal treatment?  P: nothing at all  I: ok, ok |
| 12 | I. Whay did they called your disease?  P. They didn’t say anything but I told them as I have Epilepcy. I have this disease since fourteen years ago.  I. Is that since fourteen years?  P. Yes, it was before I married and when I was with my parents.I became tensioned in this community  I. Did you fall at that time?  P. I didn’t fall at that time. I was worried about this thing starting from my childhood times. I became worried when I entered into my father’s house and I became better when I was out of that house.  I. What do you mean when you enter to your father’s house?  P.When I entered to that community. I became tensioned. They took me to the holly water areas but there was no change.  I. Do you mean in the holly water area?  P. Yes, I went to the holly water area starting from my childhood time.There was no change when I entered to the holly water areas but when others entered to that area the spirit spoke as I am I am…, they spoke but mine didn’t speak. After that I feel tension. Something made me to be tensioned inside, in my mind and heart. The disease was seen even after I married. The first time that I saw this disease after I married was just after I gave my first birth. The disease was seen just after one month from the date of my baby’s birth. I was in my family at that time.  -----  I. As you told me earlier, you also went to the holly water areas.  P. Yes,  I. Your parents went to other areas to ask about you. As you told me, the female in the holly water area told the priest what your disease is.  P. it was said like this in the holly water area.  I. Was it before you start the drug?  P. It was before I start the drug. It was when I was fourteen years old.  I. Is it at the time when you fall?  P. Yes  I. Ok, did you go to the holly water area after that?  P. Yes, I went during my childhood time.  I. Are you currently going to the holly water areas?  P. Yes, I went to the holly water area. I went to Shinkuru last month which is found out of Addis Ababa. But the spirit on me didn’t speak any thing.  I. Do you mean Shinkuru Micheal?  P. Yes, I went there.Most of the people in that area will get better from their disease within three days. But I slept the whole night with tension and entered in to the holly water early in the morning but the spirit didn’t speak any thing in my case.  I. So, how can you manage the drug and the holly water together?  P. I didn’t take the drug at that time  I. Due you stop taking the drug when you go to the holly water?  P. I didn’t stop. I didn’t start the drug before.  I. Yes, you are right at the beginning but what about after you started the drug? What do you do when you went to the holly water area?  P. I took it.  I. Are you taking the drug? Didn’t you stop?  P. I didn’t stop taking the drug.  I. How can you manage it? Is it manageable to take the drug and go to the holly water area? What do you think?  P. When I went to the holly water area, the drug was with me but I didn’t take.  I. You mean when you go to the holly water area, the drug was with you but you didn’t take it. Am I right?  P.I put the drug and entered into the holly water but I didn’t show any change.  I. So, you started taking the drug. |
| 13 | As you told me earlier she also went to a holy water. Do you think that the holy water and the medication are compatible?  P: They are not compatible. ..eh.. She was able to bring an improvement by the medication not by the holy water.  I: eh are you using both treatments at the same time? …e’…were you using both treatments concurrently?  P: After we got married, I went with her once. They suggested to take her to Goro holy water. Thus we went there together. Even though she went there, there was nothing. She didn’t scream or displayed nothing. ….Okay….then we returned. She also went to hawassa. She has a brother who lives at Hawassa. He said took her to Hawassa. Although she went there she haven’t brought any change. Then I told them to bring her back to me. I went there and bring her back to here. …eh… We went to dikuman when we came back. …okay… She was examined there and they told us what her illness is and prescribed her a medication. They gave me a tablet and told me that she should take it one in the morning and one in the afternoon.  I: I mean has she ever went to a holy water after you took her to dikuman?  P: She never went there after that. |
| 14 | I. Did you drink before?  P. Yes, I drank before  I. Did you recover from the disease?  P. Yes but after some time I became ill  I. Did you become ill for the second time?  P. Yes  I. But as you said you recovered from the disease due to the traditional drug that you drank.  P. Yes, I recovered from the disease due to the traditional drug  I. How did they give you the traditional drug?  P. There is a traditional drug which is prepared from leaf  I. Who prepared that for you?  P. There are individuals who know about the drug preparation  I. Are there?  P. Yes,  I. Ok  P. When I drink that drug, some thing which seems like bile will come from my stomach and I vomited it.  I. Ok  P. It is also seen with urine  I. Ok  P. After that I became healthy but after some time the disease relapsed  I. For how much time was that drug effective for you?  P. For two years.  I. Are you going to other areas in addition to health facilities?  P. I didn’t went to other places  I. Did you go to holly water areas?  P. No, I didn’t.  I. Didn’t you?  P. I didn’t.  I. Do you know other persons who went to holly water areas?  P. Who?  I. Other person who have similar disease with you and who went to holly water areas  P. I don’t know  I. Don’t you know?  P. I don’t know |
| 15 | I፡- Did you get other treatments apart from medical treatment?  P፡- What do you mean?  I፡- For example, has you gone to a holy water?  P፡- I have been going to a holy water till now.  I፡- Are you still attending a holy water?  P፡- Yeah, I am Orthodox Christian. So I have the obligation to be baptized by a holy water.  I፡- Eh, you have gone to a holy water. What about other traditional treatments, have you used traditional medicine ‘Ye habesha Medihanit’?  P፡- No I haven’t taken traditional medicine.  I፡- Havent you?  P፡- Yeah  I፡- How is it for instant, medication and holy water are different things, right?  P፡- Yeah  I፡- Do you think that taking those two things at the same time is allright or do you thing they are against each other?  }P፡- We are taking the holy water in the morning and then after it works, we will take the medication in the evening. I don’t think that all of them have a harmful effect.  I፡- Don’t you think that they are against each other?  P፡- Yeah, I dont  I፡- Did other people told you that they are against each other?  P፡- They haven’t asked us like that. They just asked us about our problems. They haven’t asked us whether we have gone to a holy water or not |
| 16 | P. She had no such type of sickness until our 13th years of marriage anniversary…ehh…when I asked people they told me it is yeayer ganiel, and also they told me to take to metsehaf gelach and kalcha bet; and those people asked me money..ehh…and they also a rope which was used for hanging (for sucide)…uhh…they told me to provide it…ehh…and something called fuga found on gorge.  I. What?  P. It is called Fuga, we usually use it for oxen when they get sick their back.  I. Is it soil?  P.No..ehh…it is something  I. What?  P. It is a plant.  I. Ehh…okay  P. They demanded many things…a monkey leather…there is nothing they didn’t ask…uhh…I spent around 6000 birr.  I. To do these?  P. Yes…uhh…but she didn’t get cured, I last I took her to Addis Ababa…ehh…then we went to Amanuel and they gave her this since then and I used to go there every four months  I. Did you think those people (metsehaf gelach and tenkuway) could help?Who told you to go there?  P. I was worried and I didn’t know where I could find the treatment…ehh…I didn’t think it could be treated…uhh…and people told me about this kalcha and others…I didn’t know about other places…uhh…it was because of this. |
| 17 | P. I used to live in JIMA  I. Eh  P. I thought it was an emergency disease and I was trying to treat her by “dingetegna” which is so wrong. Then after since we couldn’t get tablet at that time, she starts the medication after 3 or 6 years .  I. Did she stay 6 years with the disease?  P. Yes. It relapses every 15 days. Fortunately it doesn’t let her to fall down out of our house or at market place. She is merchant.  I. So far, you get services from different places. Have your spouse ever went to holy water services?  P. Holy water  I.Ehhh  P. She doesn’t go to holy water that much.  I. Didn’t she ever go to holy water?  P. She never went to holy water.  I. What about to traditional medicine services?  P. No, she never tried it  I. Really?  P. She never went to traditional medicine services |

Conceptualization of the illness

| 1 | I. What do you think is the cause of the problem?  P. I don’t know; it is from God.  I. What do the people say?  P. As I told you before, something came from the sky when I was sitting…  I. Did something which dropped from the sky hit you?  P. No, it didn’t  I. Did that thing shock you?  P. I got scared and laid down. |
| --- | --- |
| 2 | Do you consider it to be a problem or Do you think you are sick?  P. Yes, I am a sick person.  I. Do you think you are a sick person?  P. Yes  I. What do you think is your problem?  P. I think my sickness is ‘yedem gechit’  I. What does yedem gechit mean?  P. Blood circulation  I. Blood circulation problem?  P. Yes, I can feel it when it moves around my body, I can feel it on my back and then on my neck …. (He was demonstrating by pointing to all his body parts by his hand)…uhh… |
| 3 | I. Okay then How do you understand this sickness you are suffering from, how do you explain it form your own perspective, how is the sickness affect you  P. In my thinking, the first reason is that it is from depression and extreme anxiety, that is what I think if someone is stressed heavily by him self  I. Uhhh.. |
| 4 | I. What do you think your problem is?  P. ….I know that this is the sickness, therefore, but alcoholic beverages makes me tired I feel it in my mouse so it seems stress but no other problem |
| 5 | I. In your society, what do you think is the cause of the problem?  P. I do remember how my sickness started, I had an argument with some people because of a property (land) ..ee…I get sued and I had been imprisoned here in Sodo; I failed down (’’azuro talegne’’) when I get released from the jail, it was because of the stress  I. …eee… |
| 6 | P. I got ill suddenly. When I got back home from work, I fall down while I was asleep. At out side  I. Did I heard when said before you fall down at the farming equipment  P. The farming equipment, that is at other place. My families were said that it is just anemia. I Health professionals So what did they said the illness is? Did they say it is epilepsy?  P. They said it is epilepsy  I. Eh, eh what did they said it is caused by? What is the cause?  P. The cause, it came to me. It is God’s work.  I. Is it God’s work?  P. Ya, It is God’s work. How could humans make this? |
| 8 | What is the cause? What did they tell you? What about you, what do you believe it is caused by?  P. In my opinion I think it is something related to weather change. When the weather is changed, I feel something. I feel when something happens to my body.  I. What do you mean by weather change?  P. There is Air now, right? Todays and tomorrow weather might not be right.  I. Ah  P. During that time, I don’t feel comfortable especially when I go out at dusk. It makes me something.  I. So what is the cause?  P. The cause, how could I know? It is what god brought to us. Its cause is unknown.  I. It is unknown P. Yeah, God gave me because he has a reason. I don’t know the exact causes |
| 9 | I. What do people think the cause of the sickness is?  P. In my opinion the cause of the illness is anger. At that time by chance...ehh... it was in the evening...ehh... I came back from work at the night and went to sleep ...ehh... I got sick at my sleep. I did shout but I didn't know what I was doing ...they said it is a symptom... in the next day I get better and continued my normal life. When I came back from work they told me what happened in the other night. And they said it was likift... it has been a long time....ehh... after that I continued the medication and I became stable.  I. What do you think the cause is?  P. I don't understand it yet  I. You don't understand it?  P. Yes, I think it is a collision in our head.  I. What does collision mean?  P. What I mean is you may think about something...eee...I had something in my mind ...eee... however I couldn't achieve... I wasn't grown up with my parents ... uhh...I had been raised by my aunt; my parents was separated and my mother was with another person...uhh... and I was emotional because of this so I think my sickness could relate with it. |
| 10 | I. Okay, You’re taking the medication and you are still suffering the problem, However do you think, do you believe that you have any sickness?  P. I don’t think  I. Don’t you think the mental illness you are suffering from is not an illness? Don’t you think it is not a problem for you?  P. I don’t know  I. How/ why?  P. I don’t know any thing  I. Do you think you are a healthy person?  P. Yes! As I see it I think I am healthy person  I. But you said the illness is suffering you, you are felling frequently?  P. But, I am okay by the healing the Holy water, then I stop using the medicine  I. Did you stop taking the medicine?  P. Yes I stop taking it!  I. Are you healed by the holy water? You are not felling anymore?  P. I was taking the medicine throughout the month and I finished it, .. uhhh….. but I was felling at the same time, while the medicine was finished I went to the Holy water, I was better to a little extent, now I am better |
| 11 | I: yes…ehh…no, am asking about the persons at your home  P: they said its “Azurit”. I was at the war zone previousely…ehh…something bad happened to me and the disease starts there, …ehh… after it started there, the episode commences everytime…it started every two or three months but after I started taking the medication, I get better  I: what do you mean by “something bad happened to me” ?  P: when I was at the war zone…do you speak Oromipha?  I: no I don’t  P: I did go to the war zone…ehh… and I was combating…ehh…during that time when they shoot me…ehh…  I: did you fall?  P: yes  I: then?  P: after that, I think the devil has got in to me that’s why the disease starts every month |
| 12 | I. I am asking you about your health condition. Do you consider your self as you have health problem or as you are patient?  P. Yes, I am looking myself and saying as I have health problem and I am worried about the future.  I. Do you think about your disease and what it is?  P. I don’t know what it is but I am much tensioned and my problem is tension.  I. Do you tell me how you became tensioned?  P. The problem that I am feeling rises from my heart and brain. I became tensioned. When I speak with other person, I will see monsters.  I. What kind of things are they?  P. the so called monsters  I. Monsters?  P. Yes, I see something in my heart |
| 13 | I: What is her illness?  P: Epilepsy  I: epilepsy? P: Yes I: Eh  P: She had a mental stress. Since she is taking the medication, she is very well now… Since the medication they are giving her is helping her a lot, she is very well |
| 14 | P. I don’t know but some said as it is bird.  I. Bird?  P. Yes, there is a type of bird which is fly at night and they called it bat.  I. Ok  P. If that bird did some thing…….  I. What did the bird do?  P. If that bird rotates on you, you will become ill.If the bird rotates on me, I will become ill but I will drink traditional drugs and become healthyI. What do you think about this disease? Do you think as it is disease like any other disease? Or are you still thinking as it is a disease which is caused by bat?  P. My disease is only epilepsy.  I. I am asking you about the epilepsy. What do you think the cause for the epilepsy?  P. It is bat.  I. Is that bat?  P. Yes |
| 15 | P. This means, there is something that will make you fell down or fit. Like ‘mich’. It has something that will lead to seizure.  I. Is it ‘mich’  P. Yeah, it is a coincidence.  I. I am asking what could be the coincidence. I am asking because I do not know what could be the coincidence.  P. Coincidental means for example if I started from myself  I. Yeah tell me about your self  P. Once I had a fight with my brother and I got upset  I. Okay  P. When I got upset at that time, I had a seizure and fell down in that place. It was in Idir. Starting from that they have assessed me and have been taking a medication  I. Eh  P. And then occasionally in two or three or four months, by coincidence I got a seizure. It is upset.  I. So you think that your illness was caused by upset  P. Yeah, to me in my side |
| 16 | I. What is it? What is this problem?  P. Her sickness started 13 years after we get married  I. Okay  P. I don’t know how it started her and she doesn’t know too.  I. What type of sickness is it?  P. It is “yeayer Ganiel” evil sprite  I. Ganiel  P. Yes, people used to say it is an evil act. |
| 17 | I. What do you think the cause for this disease?  P. We call it ‘AZURIT’. It will make her fall down.  I. What is the origin or the reason for it?  P. The cause, from the beginning, on 1988 I was getting ready for my daughter’s wedding ceremony. When the ceremony left with 6 month, the thieve brock in to my house and  I. Which house?  P. There was a heavy rain. They dig my house.  I. Eh  P. Since I am a man, I panicked just for the time being. However, they hurt me and stayed for about one hour to take my assets away.  I. Eh  P. Aafter a weak, early in the morning, she falls down. I thought it was “dingetegna(emergency)” , so I gave her “dingetegna”. But she couldn’t chew it and it even makes her to form foam in her mouth. I was out to ride my horse. My daughter run to me and told me that something was happened to my wife. |

Stigma

| 1 | 0 |
| --- | --- |
| 2 | 0 |
| 3 | I. okay what was the perception of yourself and the perception of the others concerning this sickness before you went to a clinical treatment  P. They think it is a devil /Satan  I. What do you mean by Devil/Satan  P. you may wonder about it they think it is a devil … Uhh..  I. What is it about Satan/ it is a sickness caused by Satan  P. Yes they think it a sickness because of Satan .. . uhhh…  P. They think it is a Satan speaking in the sick person and they talk to it in Guragegna  --  I. What do people suggest about your sickness; what is their reaction?  P. they feel sorry for me  I. Do they?  P. Yes they feel very sorry |
| 4 | I. Uhh. Is there any stigma?  P. Stigma if it around the area …..The people who are living here feel sorry because this sickness is difficult ……  I. Uh  P. Such kind of things…for them and for the family…….advice ,first advice  I. What are you saying?  P. Why? On those who were ill before, you should not laugh. You should not show them stigma.. Most of the time I was advising them like this. |
| 5 | I. You do have worries?  P. Yes, if I fall there…ee… people might say, he is sick or if something happens to him and being with him…. By thinking those sayings might make me angry….  I. Do you think they see you down?  P. Yes, those things..  I. So, aren’t you going because those things?  P. I usually don’t go, I don’t want them to see me like this.  I. You are not going because you don’t want to or?  P. Yes  I. Is it because you are not able to go?  P. If they see when it happens it is difficult with the society, it might be difficult to live in peace.  I. What is going to be difficult?  P. He is sick  I. Okay  P. We are doing a labor work, it is planting (digging) enset …eee….they might think I might hurt my leg.  I. To protect such thinking..  P. Not to let them to think like that or not to stop me working; if a person couldn’t be called by someone to work….ee… it will isolate that person, so I don’t want them to see I am sick. |
| 6 | 0 |
| 8 | P. I heard when people say do not go near them. When I went to some place, mourning, a man convulsed like this. They said don’t come closer to him and other things  P. The people  P. When they said this, I feel bad since I have that problem too  I. Ah  P. Since I feel bad, I take out my coat and  I. Ah  P. I put my jacket on him. And when he gave me back, what can happen. It is just a problem of weather not God’s something or other thing  P. I will be conscious and will stand up and I will go. But I will request God please when I got ill now,  I. What do you request?  P. Don’t make me ill infront of other people. Make it when there is no one  I. You will say that make me ill in an empty place  P. Yeah, I will tell him to protect me and it is good if I got ill alone.  I. Why did you say like this?  P. Because they had a memory of the other individuals. |
| 9 | I. Do the others think your illness as an illness?  P. Yes, I experienced something a year ago while I was sick...eee... there a traditional belief that it is contagious. But thanks to God I am fine.  I. Contagious?  P. Yes, people used to say don't hold or touch the sick person when that person has a seizure and release his/her saliva because it is contagious... I used to feel bad ...eee... through time I am getting better and I have explained for my family members who had this type of perception about the sickness. I have received the treatment and it is good. P.When I came here many people had fear because they didn't want to be they are on medication.  I. Didn’t they want to?  P. Yes, there were teachers I know  I. With epilepsy?  P. Yes; but they don’t want be they have epilepsy.  I. are they taking medication?  P. Yes…eee… I can understand that situation however I was like that at that time. I understood through time that it is curable if I take the medication properly. |
| 10 | 0 |
| 11 | P: no, because presviousely I was afraid the illness might occur there  I: ok  P: I was even afraid of peoples while talking because I feel like they make fun of me…ehh…I was very terrified because I felt like I might fall over there |
| 12 | 0 |
| 13 | I: No I don’t mean that. What I want you to tell me is the burdens you encountered because of her  P: yes the difficulties I faced because of her illness are, when she was ill and troubled us and got lost, people suggested to me to divorce her. I refused to divorce her since it is not her fault. I am struggling that since it made happen on her by God, God will cure her. While I was continuing to struggle God brought this medication and cured her. She is recovering |
| 14 | I. What did they said about the causes of your disease?  P. I know nothing.  I. Are they saying as it has no cause?  P. People say as epilepsy is a bad disease |
| 15 | 0 |
| 16 | I. Do the others think it is a problem?  P. People say it is communicable.  I. Did they say it is communicable?  P. Yes, people use to say not to help her when she fall sown, because they believe her sickness will transmit to the person who help her.... Ehh... we use to go to attained mourning if a relative dies ...Uhh... At a funeral the noises use to cause her stress and let her fall down....Ehh...The people gave her permission not to attend.  I. Not to go?  P. Yes, in peoples’ gathering...Ehh... Yes, they gave her permission by saying “let God protect our family from this type of sickness and let God her ”... Ehh... It is bad sickness, it doesn’t give a time. |
| 17 | P. There was this place called “limugenet”…  I. Eh  P. There was this boy called Birhanu. His parents were older. He used to fall down. But we all were scared of him. We have never tried to help him.  I. Why?  P. We thought, if we touch him, he might move around  I. Okay  P. We will let him do whatever he wants to do. Some people even run away from him |

Psychological Impact

| 1 | 0 |
| --- | --- |
| 2 | P. At that time…eee…I had intention to disappear from home but not to end my life  I. Why?  P. There was a conflict between my mother and father.  I. Were they fighting because of you?  P. No, it was not because of me, but I always felt angry and questioned why I couldn’t reach where I have to be.  I. Is it a kind of hopelessness?  P. Yes, it is like that. |
| 3 | P. I don’t know what to say, I feel stress when I am thinking about it, I always make my children worried my father, my husband and my children because of this sickness, I cannot explain it …. Uhh. ….  This is how I fell  I. Okay  P. some times when you are with some people, and when I collapse from the sickness, I may feel bad feelings and as I were with many people, then I fell so much despair, as a result I always prefer to be alone for some time,  P. Yes! I want that isolation, I don’t what people see me like that, I don’t what to expose my disability and,  and my sickness, so I what that loneliness  I. Can you explain it more?  P. I mean when I feel, that happened during those times I don’t want people notice that.  I. Okay  P. You know what hurt me the most is, I am victim of this sickness as well as the other one, so it is very  painful to think about two sicknesses  I. okay  P. You can imagine the feeling; even when I feel inside my house I don’t feel comfortable with my families especially of my husband’s sensitivity, I worried a lot with these things |
| 4 | I. So, are you thinking that you have problem or are you thinking that you are a patient?  P…Sometimes I feel bad when I see myself lagging behind on my work.  I. So, What did you think your problem is?  P. …Even if I able to do, I am afraid of this sickness and I am lagging behind.  I. Why do you afraid of the sickness?  P. As I said I am working on the farm.  I. Ok (Uhh)  P. That is why  I. Uh  P. I am living in the forest.  I. Ok (Uhh)  P. When my work opposes with it I usually I feel stress |
| 5 | I. What I mean is, do you think it is a sickness?  P. I understood it is a sickness, and this makes me more worried about my children, ….not about myself…uhh…I am afraid to die, leaving my children behind. however, I have been drinking “tela ” ; I am a head of a family and I am managing my family by doing daily labor work, and tela ( traditional drink) use to give me strength to perform my work and gives me happiness for a short period..  --  I. What I mean is, do you think it is a sickness?  P. I understood it is a sickness, and this makes me more worried about my children, ….not about myself…uhh…I am afraid to die, leaving my children behind. |
| 6 | P. Now for example I was taking the medicine for some time and finally I hated it. When I took it, I fall down at the market on a day time. We come here after that.  I. You come here after that?  P. After that, I hated my own life  I. Why?  P. Because I suffer a lot in my young age, understood?  I. Eh  P. Today, a man like me  I. Eh  P. Today I won’t expect help from others. Do you understand? I don’t live my life by my self now. I have to get help from others. I didn’t work and manage my self. However today a man in my age will work and will achieve his goals and grew up. I feel sorry by my luck. But it doesn’t mean anything. It is better to die than suffer all this. Do you understand?  Today I won’t expect help from others. Do you understand? I don’t live my life by my self now. I have to get help from others. I didn’t work and manage my self. However today a man in my age will work and will achieve his goals and grew up. I feel sorry by my luck. But it doesn’t mean anything. It is better to die than suffer all this. Do you understand?  I. Why?  P. This is not life. This kind of life! Why did I become like this in this time?  I. Eh when people told you that you have epilepsy, as you told me before you knew before you came here, what did you feel? What did you feel when the people told you that your symptoms are signs of epilepsy?  P. I felt very bad.  I. Eh  P. I felt very bad, how couldn’t I?  I. What do you mean?  P. When they told me that it is epilepsy  I. Eh  P. I hated my self a lot.  I. Eh, have you heard about epilepsy before that?  P. No, I don’t know its name or what it is.  I. Eh  P. I just hated life.  I. If you don’t used to work small things in the past, are you working now?  P. For instant, there are some days where I sleep the wholeday.  I. Till now?  P. Yeah, sometimes I feel that if I go outside I will fell down on a stone so I ill slept the wholeday. When I feel relaxed, I will go out and work some small things |
| 8 | P. Yeah, Sometimes I think that from me some of my friends  I. Ah  P. Sometimes I criticized God that why didn’t you made me like them, what sin did I commite that God give me this  I. Ah  P. Then I will come back to my senses and I will say okay aren’t you the one who created it |
| 9 | because it is a problem which makes you to lose your mind …ehh…so controlling the condition by taking the medication and the advice will be helpful. For example me, I use to feel ashamed when I wake up because I don’t know what I have done when I was sick. |
| 10 | 0 |
| 11 | and I was thinking like, why don’t I throttle my self rather than falling down in to an abyss and eaten by a hyena..ehh…or rather than drowned in water…ehh…I said its better to throttle myself …and my families were very terrified and they were looking after me…ehh… then, they took me to “Dikuman” and from “Dikuman” they referred me here.I: but what did you feel when you found out that you are having this illness?  P: I feel so bad, I was very dreadful because I felt like the illness might push me in to a fire and it might kill me and I still feel dreadful…ehh… even when I am drinking coffee at home I sit far away from the fire…ehh… and am so happy now that I am getting better and I am currently working as much as I can.  I: but what did you feel when you found out that you are having this illness?  P: I feel so bad, I was very dreadful because I felt like the illness might push me in to a fire and it might kill me and I still feel dreadful…ehh… even when I am drinking coffee at home I sit far away from the fire…ehh |
| 12 | I didn’t fall since I begin this drug. Everybody knows about this. My current problem is tension. I will be tension in my mind and heart. This is my problem and it will be solved God willing. After I started this drug, I saw many changes and the drug is very effective. I will go to them as soon as I finish my drug. As I told you earlier, I will take one tablet in the morning and four tables at night. As they told me, the drugs are very important also for depression and hallucination. This drug is very effective. I saw the change. Thanks to God, I am getting better and better.  I. Did you go to health facility with your husband?  P. Yes  I. Is that after two months of your marriage?  P. Yes, but the health workers in the health facility said as I am healthy after diagnosis. My problem is only tension. I feel it in my heart and on my mind. I stayed in this way and after one year of marriage, I gave birth. The problem was inside me and when my baby was one month old, I saw the sign of this epilepsy disease……. |
| 13 | I: So you are saying you underwent though lots of sufferings?  P: Yes, very much. How much is suffering all this years? |
| 14 | 0 |
| 15 | P. I felt very sad. I felt sad since I become like this in my young age.  I. Eh  P. This is because I will go neither to a weeding nor to mourning  I. Eh  P. When I heard noise, I feel very ….  I. Does it disturb you?  P. Yeah, so don’t even go to animal’s butchery, I mean to a place where animals got butchery  I. Why?  P. It is just when I saw it, I feel ill. |
| 16 | I. Does she refuse to take sometimes?  P. Yes, sometimes she hates her life and says she doesn’t want to take the mediation and she want to die…ehh…I always tell her that this is the treatment so she has to take the medication. |
| 17 | 0 |

Family Burden

| 1 | 0 |
| --- | --- |
| 2 | 0 |
| 3 | I. Does the medication; bring some impact on your income  P. Yes very very much, look what happened …uhhh…. I will take the medication……uhh… and then I take care of my children …uhhh…there are also things to take care of the fertilizer, I have to pay my debt, I have the skill of painting walls, I work that , Thank God I do my job while I am taking the medication, if not …  I. If not you will not go to work?  P. Everything is dark  I. You told me, your father was taking care of you before?  P. Yes  I. And then your brother, and now you are doing it on your own, or is there someone else  P. No I am doing by myself, my support is God, I am doing by self  -------  I. How are you taking the medication, are you taking it yourself or someone will help you reminder?  P. Now I came with my child,  I. What about in other times, is there any time you come alone?  P. Yes No one will come, I came alone  I. So you came alone without the support of anyone?  P. Yes  I. Do you need someone to remind you about your appointment?  P. No I have a recording card  I. Is it enough?  P. Yes  I. So you do everything on your own?  P. Yes  I. You don’t need the support of anyone?  P. Yes I don’t need |
| 4 | 0 |
| 5 | P. My family members?...when I get sick I use to lose conscious and that always make them concerned and they think I might die  I.…uhh…  P. I use tell them not to worry….  I. What did they say is the problem?  P. Even though I have a concern that I might get hurt when I lose conscious, I don’t want them to be worried; but they couldn’t avoid it because they are my family. |
| 6 | P. People will hate you even your family. They will help you to get treatment and did many things to you. After that what can they do they will get tired and will hate you. Your family will not like you, if you don’t go out and work and if you don’t have money and if you can not able to try your chance by your self. They won’t like you if you just sit at home. Now I left my family and started to live by my self for their sake. I didn’t lose anything while I was there. But I didn’t want them to be worried about me and I didn’t want to be a burden on my families. So I left and preferred to stay away. I thought that if I die it is better to die here  I. What about other people, did they think it as a problem?  P. Many people have finished what they had, their assets, because of me  I. What do you mean?  P. They have taken me to several places hoping that I will be cured.  P. Everyone will go to work hard, understood? He was not willing to come here today at first. But I begged him and he come. This is because he has a work and he has childrens, understood?  I. Eh. Yeah  P. So that is why  I. Then how is he taking care of you?  P. He will come to my home and will do everything I need.  --  I. Did you start living by your self, after you started taking your medication?  P. No  I. Eh  P. I used to …. and pay rent before too.  I. By begging from people?  P. No not by begging. My relative used to pay to me.  I. Because you told them to support you right?  P. Yeah  I. I don’t mean you beg or other thing. Ehhh but now you are started to work little by little and pay yourself right?  P. Yeah  I. Isn’t it?  P. They will pay for me.  I. They will pay but you were also adding some right?  P. Yeah  I. So it means you have some improvement |
| 8 | I. Your families as well as the community  P. Yeah, they worried a lot about me. If I go outside, they don’t think that I will comeback all right. When I went to a farm work, my sisters from where they are as well as my wife don’t trust that I will come back home allright or they worried that I will fell or hit by yoke (farming equipment) and an ox will stab me P. Yeah, a lot. After now thanks to God I have children. I do only some works.  I. Ah, are your children grown up?  P. Yeah. They support me in some works.  I. Okay |
| 9 | 0 |
| 10 | 0 |
| 11 | 0 |
| 12 | I. Your health condition is getting better. Do you think as it is useful for your husband to work his work properly?  P. Yes  I. How was it in previous times?  P. my tension in previous time was for him because I was very sick and he was unable to do his work because he cares for me. If I am healthy, he can do his work with out problems and I am wishing one day I will be healthy and will do work as everybody and in that times our living condition will be improved.  I. So, you are saying as your treatment and being well will have contribution to improve your life. Am I right? This is mainly because he can do his work properly.  P. As you know, currently I am taking this drug and getting better as a result he is doing his work properly. |
| 13 | I: Although she is getting better now, she was ill. Thus, how much effect or burden did her illness had on you?  P: In the past I …e’…I had passed through several things.  I: For example?  P: Like when she was ill, I had to stop my work and sit in the house. If a man sit in the house, what can he do…..okay….What can he work if he sits in the house?  I: Did you quit your work and sit in the house? P: Yes  I: Why?  P: In the past since she was ill and didn’t know anything, I worried that she may bite up children or someone or she may encountered a problem  P: I able to carry out my work daily now since she is fine. In the past I couldn’t work daily  I: Why?  P: Because I had to look for her. She used to get lost in the night  I: Did she used to go away?  P: before yes. If I didn’t enter to the house at 7:00pm, she would leave the children and got lost. She used to run….eh…because of that I have to go to the house early without doing any work….eh… There was a time where I spent the whole day in the house.  I: You have said earlier that your life has showed some improvement since you started working and since she got better by taking the medication. Apart from that do you think that dispending the medication and the transportation fee has a benefit regarding reducing your expenses?  P: Yes, regard to reducing cost, the one that is supposed to be spent for medication and transportation will be used to cover other house hold chores. It is useful if it is spent to buy something for the house hold.  I: eh so are you saying that for the improvement of your life, her recovery has a contribution?  P: yes, a lot  I: For your work as well? P: Yes  P: yes the difficulties I faced because of her illness are, when she was ill and troubled us and got lost, people suggested to me to divorce her. I refused to divorce her since it is not her fault. I am struggling that since it made happen on her by God, God will cure her. While I was continuing to struggle God brought this medication and cured her. She is recovering.  I: So you are saying you underwent though lots of sufferings?  P: Yes, very much. How much is suffering all this years?  I: Do you have children? P: Yes  I: Are you the one who raise the children? P: Yes  I: Do you have other families who are supported by you? P: No  I: Is that just your children? P: Yes  I: How do you raise your children?  P: I raise my children as my capacity permits. I can’t steal what I don’t have.  I: What do you do for living?  P: Tailor  I: Do you make fitted cloths?  P: Yes, I work whatever I get and take the money home. |
| 14 | I. But you told me as you have changes after the treatment  P. Yes  I. How do you describe the change? For example, the change in communicating with other persons, participating in social activities and the change that you observed in your working ability.  P. The change as I told you before is I am leading my house properly.  I. How and what is the different thing that you are doing?  P. The different thing is now I am living with my wife peacefully and I am teaching my sons and protecting them |
| 15 | I፡- Don’t you used to work that in the past?  P፡- Yeah, I only used to work sometimes. My families were the one who used to do that.  I፡- Yeah in the past  P፡- Yeah I couldn’t trust myself to go alone.  I፡- Do you think that this could bring a change to your life or to your relatives’ life? Since they have stopped accompanying you to the health center, do you think that they can do their work and this will make change to their life?  P፡- To whom, to me or to them  I፡- What difference could it make if they accompany you or not?  P፡- They can do some works and other many things until I comeback from the health center.  I፡- Yeah!  P፡- They will wait me doing their works. |
| 16 | I. How does her sickness affect you?  P. I always think what it is?  I. What?  P. I am worried about her  I. How about on your job?  P. I am always think about her while I am working….ehh…I am worried something might happen to her while I am on work and away from her…ehh…I always think about her.  I. So, do you think this could create a problem on your job, your farm work?  P. Yes, her sickness affects me.  I. What?  P. I can do nothing even though I am worried sick…ehh…I can do nothing…ehh…so, I am not focus on my job.  I. Don’t you?  P. My attention is with her…eh…she might feel sick or fall down…ehh… we use to eat kocho so I am usually stay with her until she finish baking. P. Yes, whenever I went to Addis Ababa I used to ask my neighbors to look after her…ehh…yes…ehh… ask children to stay with her in the evening…ehh…I used to go after I settle these things otherwise it was difficult to leave her alone.  I. Do you think her sickness affects your social activities like wedding, mourning, edir…and the like?  P. Yes, wherever we go whether we go to a wedding or a mourning ...uhh... I have to help her to relax and not worry even though it is in the neighborhood  I. Do you think bringing your relative back for regular appointments affects you?  P. Yes, whenever I went to Addis Ababa I used to ask my neighbors to look after her…ehh…yes…ehh… ask children to stay with her in the evening…ehh…I used to go after I settle these things otherwise it was difficult to leave her alone.  I. Do you think her sickness affects your social activities like wedding, mourning, edir…and the like?  P. Yes, wherever we go whether we go to a wedding or a mourning ...uhh... I have to help her to relax and not worry even though it is in the neighborhood.  I. Have you ever been in able to attained because of her?  P. Me...ehh.. No, I will take her....ehh... My attention will be with her  I. How is your living status/standard?  P. My living status is medium  I. uhh...uhh...how is the change?  P. It has been decreasing since she got sick.  I. Has it been decreasing?  P. Yes...ehh... she used to work ... ehh... she used to work at home and also went to the spring... but now she is unable because of her sickness.  I. How is your work?  P. I am working as much as I can even though my attention is with her.  I. Do you think that her sickness affects your work?  P. Sometimes if she is sick I might not able to go. I stayed there the whole day fearing she may get sick.  I. Do you think the health center fee and transportation fee affects your life?  P. Yes, I am going there because her, I don't have any other reason ...uhh...so then if I don't have I have to sell what I have and go... uhh... if she wasn't sick we were not supposed to spent this much...uhh..I think like that. and I feel  I. So are you saying her sickness has an effect on your work?  P. Yes, I put my work away when I go but the cost is still on me so this means her sickness is affecting me... uhh...uhh.. I is affecting me.  I. Regarding the follow up ...uhh... do you think it is a burden to you?  P. Even though it is a burden there is no other option ...uhh... there is no any alternative place to stop taking the tablet and go ... tablet...so taking the tablet properly is the only option whether it is appropriate or not ....uhh... in any case it depends on the physician order.  I. How are you handling her sickness and the family administration?  P. Everyone is worried.  I. Okay  P. So  I. How is your responsibility?  P. My responsibility...there is no one who can share since our children are not grown up enough...uhh... I can't say they can share the burden and watch her.  I. The children who are in the house, are they small children?  P. Yes  I. So, are you the one who is covering everything?  P. Yes |
| 17 | P. previously, because she used to get sick while we felt asleep, i always feel like as if she is sick. Even now, I always panic and get awake in the midnight to check on her.  I. Eh  P. She used to get sick every 15 days. During that time I used to sense that she will get sick by my self  I. You?  P. Ya I used to sense it like a dream or nightmare.  I. Eh  P. During that time, while we asleept, she will got sick…  I. Eh  P. I had this sense telling me that she got sick. I was afraid that I might get sick too. But thanks to God I am healthy.  -----  P. Yes, I worry because the disease might shorten her life span. Unless the disease is totally cured with the drug; the drugs are actually just preventive. Therefore, as far as the disease is not cured, I always get worry by assuming she might live shorter.  I.Ehhh  P. I sometimes feel sad  I. Eh  P. I mean I feel sorry for my situation  I. Do you feel sad?  P. Yes  I.Eh  P. I sometimes feel sad |

Psychosocial support

| 1 | 0 |
| --- | --- |
| 2 | I. How much did the health professionals spend time talking with you about your difficulties?  P. They don’t discuss … okay…they usually give me advice about how to manage my sickness.  I. Don’t they ask you about difficulties you have been facing as a result of your problem?  P. About problems I faced?  P. No, they usually don’t…uhh…you are the one who is having such discussions with us.  -------  I. So, How did you feel about not talking to the health worker about your problems?  P. I don’t feel good, but they are busy to do this, so I simply came here and take my medication.  I. Do you mean that you are not happy for not discussing about your difficulties with the health professionals?  P. No, I am not |
| 3 | I. Okay.. Okay.. when you come to the health facility, do the health professionals discuss with you about difficulties you faced due to your sickness (Epilepsy) or for example you have epilepsy and you have been failing down many times?  P. Yes  I. You have been feeling ashamed in the society  P. Yes Yes  I. So did they discuss with you about this?  P. Yes, it is right, they told me …ee… what do you feel when you run…eee… what do you feel?...ehh… I am feeling like this…ehh…they use to ask that do you think you need help from the society? And I said yes when I fail down.  I. Did you say to them that?  P. Yes  I. What do you think is the benefit for you sitting and having discussion with them?  P. Look, for example I fail down here now…ee… you gave me advice now…ee…you have asked me… ee… so I am not going to feel ashamed why I feel I am a normal person…ehh… they are good. For example if I fail down when they send me here …ehh… if he ask me I am not going to feel anything because he can understand my situation.  I. What?  P. But if they are not educated, when I get hurt I was worried about that people will question why I am like this? I used to feel ashamed that people may assume I was failing down because of different reasons.  I. Do you think their advice help you to reduce your feeling of embarrassment?  P. Yes Yes, definitely |
| 4 | I. Do they discuss with you on the problem that you are facing because of the sickness?  P. ...Only one day that I was asked.  I. Uhh  P. They were seating here like you are now  I. Uh  P. He asked me difficulties which I faced because of my sickness  I. Uhh  P. In your opinion, what are the problems you would expect in the future?  I. Uhh,uhh, Do you think asking your difficulties will help? And To what extent it was helpful?  P. To strengthen ourselves and it will help us not think about such things.  . …….At the beginning I was feeling that…  I. Uhh  P. But such kind of things… after the physician asked me he told me that I should not think about this….  I.Uhh  P. … He advised me like this ,if you survive .you will do all, If you are cured ,you will have what you want, If you think about the sickness ,you will be confused, you may do unnecessary things he advised me by saying all this things. I have taken all the advice.  I. Did it help you?  P. Yes |
| 5 | I. About the service delivery what things should be improved and what things should be continued?  P. If I can get an advice which can help me to minimize my stress…eee.... it is nice…eee…there is bolting or getting angry if there is noise  I…eee…  P. So, if there is a way which can improve |
| 6 | I. Tell me if there is something you want to tell me  P. To you  I. Yeah, not exactly for me, in relation to this  P. I want you to help me. I want support. That’s it.  I. Is it in terms of treatment?  P. Help me in terms of the treatment as well as in other things. I want your support.  I. Is that what you want to say?  P. Yeah |
| 8 | I. What are you going to say If someone come to your home and if you discuss about the illness, If he told you it is good if you do this or if you say this or if they support you in your work  P. I will be happy how I could say no when I got someone to support me.  I. You will be happy  P. Yeah  I. Ahhhhh  P. It means sharing ideas, I will accept his idea and he will mine  I. Ah  P. I will be happy. I will learn from their experience and they will from mine. |
| 9 | I. Do you think it has an effect if the health professional asks you? Or did the health professionals ask you how you are or how your status is?  P. Yes  I. If they did, how did it help you?  P. In my opinion or in our tradition it is good if someone asks you how you are doing.  I. So  P. Concerning the professionals, I will be happy if the health professionals ask me.  I. Is it only for your happiness?  P. It is like me like that.  I. Do you like to be asked?  P. Mentally  I. Does it have satisfaction?  P. it is a relief. |
| 10 | I. so nobody ask you about your health?  P. Yes nobody ask  I. but will you be happy if they ask you or you will be sad?  P. I would be happy if they ask, but nobody ask  I. Nobody ask what if they sit with you and discuss?  P. I would be happy, my self |
| 11 | I: do the doctors here, do they ask you about your illness and the problems caused by the illness? do they discuss this with you?  P: yes, they discussed with us. They tell us to do what the doctor told us to do…ehh…they are even come to our home to consel us  I: I am asking you about the health workers here  P: its all the same here  I: do you think that is important?  P: yes, that is very important… I: what is the importance?  P: the importance is…ehh…when ever I come here, they ask me about the progress I have…they ask me whether I have improvement or not… they don’t just provide the tablets |
| 12 | I. Do you discuss about your problems with them?  P. Yes, they will ask us about our health conditions, the disease and about the drug that we are taking.  I. Did they ask you?  P. Yes, they did. I am also telling them as eventhough I am taking the drug, the tension is available in my mind and heart and sometimes I will also tell them the progress that I saw and the beneftis that I got from taking this drug.  I. Are you telling them?  P. Yes, I am telling them. |
| 13 | I: So, they will sit down and talk to you  P: Yes and they also told me and advise me that there is no problem and to let her enjoy. They gave me so much advice.  I: eh what did they advise you to do?  P: They told me to relax indulgence her since the illness don’t like stress.  I: They told you that P: yes  I: But do you think that is useful?  P: Yes, I think it is useful. ……eh…If you do something different from the usual, in addition to giving your love, there will be surely a difference.  I: Is that how you think?  P: Yes, I also think like that. |
| 14 | P. They advised me not to harm my self.  I. Was it advice?  P. Yes  I. What did they adviced you? Please tell me examples  P. They told me not to harm my self and I said them as I am protecting my self.  I. Ok, so I am asking you whether it has benefit for the patient or not.  P. Yes it has benefit  I. Ok  P. They told me this by wishing well and thinking for me.  P. It is useful for me.  I. Tell me how?  P. They told me not to be angry. If I am angry, I will harm my self but they told me not to be like this. This is an advantage for me. I am happy for the advice that they gave me.  I. Is it good?  P. Yes  I. Do you think as it has effect on the patient?  P. Yes, definitely. |
| 15 | I. Did they speak with you while you are sitting in a chair?  P. No, we don’t sit together.  I. What do you feel about that? What do you feel since they didn’t ask you?  P. I think it is not a good thing.  I. I want you to tell me why you think it is not good?  P. If we discussed like this in a table, we may tell them what problems we have if we have some problems and if we don’t have any problem, we can tell them our improvements. So I am saying that am not happy since they didn’t asked me anything.  I. Do you think that it is good if they have asked?  P. Yeah it is good if they have asked. Sometimes we may have some problems. Every day is not the same.  I. You are right  P. It is good if they told us what is good and what is not good so that we can avoid mistakes. But they haven’t asked us anything  I. Haven’t they?  P. They haven’t  I. Eh eh, you haven’t had such thoughts. But do you think it is good that the doctor ask the patient such question?  P. Yeah  I. Why?  P. I think it is good to get a solution for our problems and to get advice if we have some problem. |
| 16 | P. They use to give us advice at a time like this…ehh…they don’t tell us about anything when we come to take medication…ehh…we usually only medication on the appointment day.  I. Is that it?  P. Yes  I. So, there is no discussion or advice?  P. No, for example today, we came here because of you…ehh…you are giving us this advice; we will leave when we finish. |
| 17 | I. Isn’t there counseling?  P. The only advice that they give us is not to stop taking the tablets  I. who used to say that to her?  P. The physician himself  P. It is on appointed date they that they told us to come  I. I mean , do you come on the appointment date?  P. Yes  I. Do they sit with you and give you advice?  P. No  I.Ehh |

Screening for Suicidal Ideation

| 1 | 0 |
| --- | --- |
| 2 | I. Look, some people with this problem might lose hope and think like it is the end of their life…okay…. For people with this problem, health professionals might ask whether they had been feeling like giving up on life or even whether they had thought about ending their life….okay….Did you experience the health professionals asking you these questions?  P. Yes, they did…uhh… and I answered their question. There might be a problem as a result of conflict between family members…uhh…. This may result stress and in my opinion, stress is the cause of this sickness.  Did you experience the health professionals asking you these questions?  P. Yes, they did…uhh… and I answered their question. There might be a problem as a result of conflict between family members…uhh…. This may result stress and in my opinion, stress is the cause of this sickness.  I. Did you ever think such things (giving up/ending life)?  P. No, I didn’t.  I. You didn’t think?  P. No, not until now  I. Do you think that it is a problematic to ask someone if he/she had been thought of dying? ..eee… or is it helpful the patient or not?  P. It is useful  I. How?  P. What I mean is, it will help him/her to manage his/her sickness…uhh…to control him/herself…uhh…it will help that person to think broadly, to calm him/herself. For example I am working on growing plants, I usually do my job by being careful; when I go to fetch water I always ask someone help to put the water in the jar for me. |
| 3 | P. then when my wife after I return home looked at my hands, I feel very depressed and I thought of killing my self ….uhhh… I said to myself it was better to die  I. Why, did you feel some embarrassment or something else  P. Then, let me tell you, I wrote a note then  I. Okay  P. And I prepared that there was also “Kocho” very high, equal to my height, it was a little high  I. Then what  P. There was also a grinder, of equal height… I was a little angry; there was my child who completed grade twelve, I feel that I am not sufficient to my children …uhhhh… I tried all they are all of the same height of me; but it was not successful I cut the rope off me with knife  I. Do the doctors asked you such questions, and did you explain this to them very well like you told me know  P. Yes  I. Do they ask you?  P. Yes they ask me if I create some problems like this in my house, or trouble my family with some irrelevant things; …uhhh… yes but if I am asked I will tell them like some head of the household who did the same like this.  P. I might seem polite for you here however I have different language/behavior at home…eee…. My sickness is not this much thanks to God.  I. Okay.. But did the health professionals ask you whether you tried to end your life or not?  P. Yes  I. As you told me they did ask you and you also have tried?  P. Yes  I. What do you think about health workers asking these types of questions? Does it have advantage or disadvantage?  P. I have prepared a letter on that day even though it was harmful; their question is helpful  I. What did they say, for example?  P. Did you think like this or don’t imagine such thing in your thinking…eee… things like don’t kill yourself … eee …. I had been thinking after I went home, if they didn’t ask me…eee… it was the result of my lack of knowledge and ignorance.  I. Was it due to lack of knowledge? So do you think it will improve knowledge?  P. Yes, I was very happy, why as I mentioned it earlier…eee… I refused to step down after I climb on the roof…ee… for example I am painting wall and roof when people ask me to do …ee.. have you ever tried to kill yourself…ee… have you ever tried to stigmatize yourself…ee… and I said yes.  I. So do you think being asked helped you to improve your life or to control /manage your sickness?  P. Yes  I. For example, how?  P. I am very happy! I am happy! for example, previously …ee… it helps me to give advice for others let alone for myself …..ee… there advice was correct..ehh… killing yourself is not easy…eee… this is the first thing and the second is, because of my sickness I had been refusing and disturbing others …ee… I am happy that they are giving advice about this. |
| 4 | P. They did ask me whether I had been thinking to end my life instead of being stressed.  I. Uhh  P. I do not think like that.  I. Did you not think?  P. Yes  I. Do you think this question is appropriate?  P. It is correct  I. It is good.  P. Yes  I. Ok, what is the use of asking such questions?  P. They use to give us an advice not to think such like this in the future and Inever think like this.  I. ……Do you think it is appropriate to be asked by the physician that you have tried to commit suicide?  P. it is very good.  I. What is the use?  P. Uhh,….they were advising me. I am not thinking such kind of things. I will not think for the future. I never thought before.  I. Uh  P. Why? …. I did not think to commit suicide but I will try to find a place to go.  I. You did not think  P. Yes ,them too.  I. Ok, you said that it is appropriate to be asked by the physicians.  P. Yes, it is good  I. But what is the use? My question is in your opinion what will be the use for the patients?  P. ….. not to think  I. It is useful  P. Uh  I. Uhh  P. It was………not to think like that |
| 5 | I. For people who have the kind of problem that you describe, health workers might ask whether they had been feeling like giving up on life or even whether they had thought about ending their life. Do you think about health workers asking this type of questions is good?  P. Yes, because I am sick  I. Due to this sickness?...eee… the health workers might ask whether you had thought about ending your life because your sickness…  P. Yes  I. Do you think asking this type of question is appropriate?  P. I don’t think I will think to end my life…I may say why I have this type of sickness …eee…. If I can go far not to hurt my family and my children when I get sick…  I. Do you think asking this type of question is appropriate?  P. I don’t think I will think to end my life…I may say why I have this type of sickness …eee…. If I can go far not to hurt my family and my children when I get sick…  P. Yes, it is good.  I. Why?  P. I have to tell them frankly if they ask.  I. How it is helpful for you?  P. For the health worker… it is good if I can answer what they ask by saying why should I hurt myself.  I. So, are you saying that being asked such type of question is helpful for the patient?  P. Why not?  I. How can it help the patient?  P. That person might learn…okay….might receive knowledge…okay… I guess so.  I. Do you think like that?  P. I have to learn to protect myself if in case I develop such thinking. |
| 6 | Do you think that it is good if the doctors ask about suicidial ideation?  P. Doctors don’t say such kind of things  I. Why?  P. If they said like that why we come here?  I. No, they are not going to ask you if you are going to commite sucide. They will ask you if you have attempted to commite sucide.  P. No they haven’t asked.  I. Haven’t they asked you?  P. No they haven’t asked.  I. If they asked, is it good or not good?  P. I don’t know, I don’t think so  I. You think it is not good.  P. I won’t accept it  I. If they asked, won’t you be happy?  P. Yeah  I. Why?  P. I think i prefer to stay alive even if it is for short time even for one or two months. Do you understand why?  I. No, I don’t understand  P. Can someone give me one day life?  I. No, that is from God  P. Yeah  I. So you think that they shouldn’t ask about suicide attempt?  P. Eh, I don’t know  I. Are you saying that one patient shouldn’t be asked like that?  P. Yeah I don’t heard anything |
| 8 | 0 |
| 9 | P. It might be difficult for some people to be asked directly. I told you before not for me  I. How about you?  P. Me?  I. For example, if a health professionals asks you whether have committed suicide or not?  P. I am not going to do such thing because of this sickness.  I. Have you ever been asked?  P. If they tell me it is for research work.  I. Have you ever been asked?  P. Yes, recently... ehh... their question wasn't direct...ehh... she asked me that ""do you think that this sickness may lead to commit suicide?  I. So, being asked such question  P. Yes  I. If you have been asked?  P. I never think like this. In previous times I had been feeling inferior, however, I don't have such feeling since I start the medication.  So I told them that such thinking never cross my mind and that is why asking such question is not good. " |
| 10 | 0 |
| 11 | I: well, for a person with Epilesy…ehh…have you ever wanted to commit suicide?  P: Ehh?  I: they might ask you or other persons like this…do you think its appropriate that they are asking like this?  P: to tell you the truth, rather than getting down in to an abyss or drowned in to the water…ehh…when I was thinking of dying …ehh…I was thiking to throttle myself…ehh… and I was thinking like, why don’t I throttle my self rather than falling down in to an abyss and eaten by a hyena..ehh…or rather than drowned in water…ehh…I said its better to throttle myself …and my families were very terrified and they were looking after me…ehh… then, they took me to “Dikuman” and from “Dikuman” they referred me here. Now, I am normal, I am doing good and I am working.  I: but, did the Doctors ever asked you like that?  P: they did ask me  I: so, is that good to ask about it?  P: that is nice  I: for what?  P: …ehh…they said, “Why don’t you take the medication… you would have been normal if you follow what we told you” ehhh… “why did you discontinue the tablet, you should take it today” … |
| 12 | When you discuss with the health professional, did they asked you about thinking and/or trying sucide?  P. Yes, they asked me.  I. Did they?  P. Yes, when they asked me this question, I told them as I didn’t think sucide eventough the tension disturbed my mind. I am praying to God. I am getting better after I started this drug. I told them in this way.  I. Did you?  P. Yes,  I. Is it good to ask such kind of question?  P. I told them as my case is tension. They are right because some people who faced such kinds of problem may think to try suicide.  I. What do you think to ask a patient this question? Is it good or bad?  P. Asking such kind of question is not bad for me because they are also telling me as I will get additional drugs in addition to this. So, they told me not to think other things and as I will receive additional drugs means a good thing in my view.  I. Do you think as it is a good thing? For example, one patient will come to the health facility and the health professional is going to ask him whether he tried suicide before or not. Is it good or bad asking such kind of question for the patient?  P. What is wrong with asking such kind of question? They will ask him whether he thinks such kinds of thing or not and then they will tell him to continue taking his drug and as another new drug will come in the future and if he takes his drug properly as he will recover from this disease. So, what is wrong with saying such kind of thing?  I. Is that good?  P. Yes, |
| 13 | 0 |
| 14 | I. Did they ask you about suicide attempt?  P. Yes, but I said as I didn’t.  I. Did they ask you?  P. Yes, they asked me  I. Did you say no at that time?  P. Yes, because I didn’t attempt it.  I. Ok, but what did you feel when they asked you this question?  P. I didn’t feel any thing. I didn’t respond irrelevant thing.  I. I am not saying like that but is it good asking such kinds of question? This is my question.  P. They asked me in good way  I. What do you think the benefit of asking such kinds of question for the patient?  P. They asked me whether I thought other thing or not.  I. Ok, but is it good asking a patient whether he tried sucide or not?  P. It is good.  I. What is the benefit?  P. They advised me not to harm my self.  I. Was it advice?  P. They told me not to harm my self and I said them as I am protecting my self.  I. Ok, so I am asking you whether it has benefit for the patient or not.  P. Yes it has benefit  I. Ok  P. They told me this by wishing well and thinking for me. |
| 15 | I. Then the doctors may ask one patient about suicide attempt. They may ask the patient that ‘have you ever attempted to commit suicide because of your illness?  P. Yeah, they have asked me how I feel about it. How do you react when you felt bad? I have responded that I am accepting it and have been living happy since it is God’s work. It is not humans work.  I. Eh, you had never had such thoughts?  P. Yeah  I. Never  P. Never  I. Eh eh, you haven’t had such thoughts. But do you think it is good that the doctor ask the patient such question?  P. Yeah  I. Why?  P. I think it is good to get a solution for our problems and to get advice if we have some problem. |
| 16 | 0 |
| 17 | 0 |

Emphasis on Medication

| 1 | 0 |
| --- | --- |
| 2 | I. What did the health professionals tell you about epilepsy?  P. The health professionals use to tell me to write all the changes I observe and bring for them, and that will help them to decide to increase or decrease the dosage.  I. That is after they gave you the medication  P. Yes |
| 3 | P. They are going to examine and ask me, then finally they will write me the medication  I. So are they discussing with you about the medication and whether you would like to take it?  P. Yes..ehh..they will tell me the time when I am going to take, whether it is in the day time or in the evening…ehh… I know this. |
| 4 | I. Uhh, Uhh…. How is the service which is given in this health station?  P. Where? , how?  I. How do they give the medical service?  P…. how to use tablet  I. Uh  P. Uh, to carefully follow the dosage  I .Uhh,  P. this is the advice that they gave us,there is no other things  I. Uhh,uh  P. …. There is nothing that they are doing. |
| 5 | I. How much did the health workers inform you about epilepsy?  P. for me  I. What did they advise you?  P. Just the existing things, like not use alcohols…eee… how it oppose with the medication  I. …uhh…  P. one the alcohol might hurt me directly and two the medication might not work properly  I. …uhh…  P. it is not good if I quit the medication…uhh…they told me all those things.  I What did they tell you about your sickness?  P. About my sickness, I don’t know how and when I am going to be sick.  I. When the health workers tell you, I think they know better than the others about the sickness?  P. Yes  I. So, what did they tell you?  P. They told me to take the medication properly |
| 6 | Don’t you know if it is the same doctor or not  P. The persons, me  I. Eh  P. I just show them my card and take my medication  I. Aha haven’t you ever meet the doctors  re the same or not.  P. Yeah, I will show my card and then they will give me the medication. That’s it. I. Okay, When they give you the health service, I mean when they gave you the medication, have they told you about your illness and have they asked you a comment or suggestions about what you need to do or shall they gave just the order?  P. No  I. Didn’t they?  P. No, they didn’t  I. Yeah  P. When I finished the drug exactly after a month, I will come and take the medication  I. Did they just give you the medication?  P. No, for someone who can understand some  I. But they don’t let you to participate.  P. Yeah |
| 8 | I. What did the doctors said about the cause?  P. The doctors told me to come and dispense a tablet every month. They said that you will be benefited from it and you will be cured. |
| 9 | I. People used think it is likift ...okay... what was the explanation of the health workers or what did the health workers think your sickness is?  P. The health professionals, they just gave me the medication.  I. what is the sickness?  P. Epilepsy  I. Epilepsy?  P. Yes, Epilepsy. If we can take the medication properly it is not harmful and also not affect our body.  I. Did they tell you that?  P. Yes |
| 10 | 0 |
| 11 | I: what did the doctors say about your disease? What do they say it is?  P: they told me to take the tablet and they they also told me that I will get better if I took the medication and I am fine now, I am normal |
| 12 | I. Why you didn’t tell them about the magic spell?  P. I didn’t tell them in the first time but they asked me repeatly and I told them. They told me as the drug that I am taking will remove it from me. God willing  I. Did they say the magic spell?  P. It has no capacity. They told me as the disease has no ability to stay and orderd me to take the drug according to the instruction and one day as I will recover from the disease God willing.  I. What did they tell you about this disease? What things did they tell you to do for the disease?  P. They gave me the drug and told me to take the drug according to the instruction. That is it.  I. Didn’t they describe about this disease for you?  P.Do you mean the health workers? The disease has no any diagnosis. I told them in the same way as am telling you. They will give me the drug and as I told you they told me as the disease will vanish one day in the future. |
| 13 | I: I am asking you about the service delivery. What should be improved?  P: Things to improve, what can be improved now?  I: You are the one who know that, not me  P: I only know about the medication. I don’t get other thing.  I: We are taking about the health care service  P: About the service, there is nothing |
| 14 | I. Ok, in what way the health professionals are telling you about this disease?  P. They know the disease  I. Yes, they know the disease but how did they tell you about this disease?  P. They told me as the disease has drug and to take the drug according to the instruction and in addition to this they also told me not to drink alcoholic drinks and local drinks such as ‘Areke and tella’. So, I am getting better thanks to God. |
| 15 | I. What did they told you about the illness?  P. They haven’t do any examination  I. Haven’t they told you anything about the illness?  P. Yeah  I. Don’t you get any advice from the doctors?  P. The advice is, don’t drink alcohol (Tela, Areque), and use the medication properly  I. Is that the advice?  P. YeahP. We just show them our card. Then they will give us the medication and we will go to our home that’s it.  I. Is he the one who asked you there?  P. Yeah, he asked me how I am doing and other things. I am telling you only about my self  I. But the doctor didn’t examine you?  P. They haven’t examined us  I. Don’t the doctors discuss things with you like this in a table?  P. No |
| 16 | P. They use to give us advice at a time like this…ehh…they don’t tell us about anything when we come to take medication…ehh…we usually only medication on the appointment day.  I. Is that it?  P. Yes  I. So, there is no discussion or advice?  P. No, for example today, we came here because of you…ehh…you are giving us this advice; we will leave when we finish.  I. What type of treatment is your wife receiving from the health center?  P. Now..ehh…from the health center, medication  I. Medication  P. Yes |
| 17 | I. What information did they gave you about her issue? How did they explain about her condition to you?  P. Is it this year?  I. Yes  P. They say that she will be cured with this tablet. They also tell us that she shouldn’t stop taking the drug and advise us to come and take the drug every month.I. From pharmacy?  P. Yes from the pharmacy  I. So, do the physicians talk to you by sitting next to you whenever you come here monthly?  P. Monthly?  I.Ehh  P. It is on appointed date they that they told us to come  I. Do they sit with you and give you advice?  P. No, it is just sometimes. For example they will call us to come on certain day. When we come at that day, they will tell us like that.  I. Otherwise, they don’t do that always?  P. No, it is not always. It is not feasible.  I. Is it not?  P. Yes  I. Ehh..Ehh  P. Therefore, it is not every time that they do that |

Emphasis on compliance

| 1 | 0 |
| --- | --- |
| 2 | I used to have a follow up in Butajira Grarbet Hospital  I. What did they say?  P. They didn’t say anything to me.  I. What did they say that your sickness is?  P. They told me it is epilepsy and advised me to take the medication without interruption |
| 3 | P. I was even told in Addis Ababa not to stop it … uhhh…. With the professionals, whenever I stopped the medicine, …uhhhh.. one day the doctor asked me for how long I took the medicine …uhh… then I told him I took it for nearly six months, then I stopped, then he told me how could I become a self-doctor; and he asked me to go out |
| 4 | I. How do they give the medical service?  P…. how to use tablet  I. Uh  P. Uh, to carefully follow the dosage  I .Uhh,  P. this is the advice that they gave us, there is no other things  I. Uh, on the usage of the medicine and did they tell you it’s side effect.  P. Yes ,it will hurt you , on the usage it needs carefulness  I. Uhh  P. You should take the medicine carefully |
| 5 | I. …uhh…  P. it is not good if I quit the medication…uhh…they told me all those things.  I. Did they involve you in the decisions about treatment?  P. Yes  I. For example, did they ever ask you questions like I am going to give you these medications and are you interested to take?  P. Yes  I. Did they usually ask?  P. Yes  I. Didn’t they just order you to take?  P. They usually tells me that are you taking your medication, you need to take this medication properly and if there is any problem related to the medication you need to come and have a discussion with us…uhh…they use tell me to come on time and discuss with them. |
| 6 | I. Did the health professionals advice you to come here for follow up and not to discontinue it?  P. Yeah, they did  I. Eh  P. They did tell me to come every month and take my medication  I. Eh  P. They told me not to discontinue the medication, not to drink alcohol, to stop using chatt and other drugs.  I. Did they tell you that?  P. Yeah they did tell me that.  I. They don’t tell you about the medication.  P. Yeah, they advised me to to take it properly.  I. Is that it?  P. Yeah |
| 8 | 0 |
| 9 | I. What did the health workers say to you? Did they tell you not to go to the holy water?  P. Not to stop taking the medication because of it  I. What else did they say?  P. If you go to there you will stop taking the medication and that will make your condition worsen.  P.will be get better and start working and then...ehhh... I will be get sick .... they advised me that I will not be able to continue my education if I discontinue the medication.  I. How was it in here?  P. I usually come here and take my medication. |
| 10 | P. I just told them, that I have stopped the medication and start the holy Water  I. Then what did they say when you told them that you stopped the medication  P. They don’t say anything the keep quite  I. Do they agree and say it has no problem?  P. No they don’t |
| 11 | I: for instance, you discontinued your medication before, right?  P: yes  I: why did you discontinue it?  P: because my mother-in-law passed away  I: ok  P: I mean, I was there at the mourning house …ehh… there was no one at home when my mother-in-law pass away… they only had females and I was crying and I was thinking to take the tablets…ehh… I discontinued for two weeks, that’s all  I: the tablet?  P: I was afraid that, they might force me to drink “Areke” and that’s why I discontinued the tablet. However, I came and tell the doctors …ehh…they told me not to discontinue never again…ehh… after that I continue my follow up  I: what did the Doctors counsel you to do?  P: the Doctots…ehh… they told me to take the medication properly…and…not to discontinue the medication…  I: so, is that good to ask about it?  P: that is nice  I: for what?  P: …ehh…they said, “Why don’t you take the medication… you would have been normal if you follow what we told you” …ehhh… “why did you discontinue the tablet, you should take it today” …ehh… “you may also go to “Areke” house …ehh…”and “if you drink “Areke” on top of the tablet we are not taking the risk and you have already signed here while you took the tablet” …ehh…  I: did they tell you that they are going to provide you the tablet and did they ask you whether you are able to take the tablet or not?  P: yes, they asked me about that and told me to take it properly…ehh… they said, “ take the medication properly as we counseled you”. |
| 12 | I. Are they asking you? Which one is better if they asked you or not?  P. It is better if they asked me. What is wrong with their question? It is good for me. They asked me whether I took the drug according to their instruction or not, whether I took the drug in the morning and at night, whether my health condition is getting better or not and they will also give me hope as the disease will disappear from me. |
| 13 | 0 |
| 14 | P. Yes, they told me and advised me.  I. How did they tell you? Please tell me examples  P. they told me to take one tablet at the bed time.  I. Ok  P. They told me to take the drug after dinner and adviced me to take rest.  I. What about other things?  P. That is it. |
| 15 | I፡- Are you taking the medication from the pharmacy with out the doctors prescription?  P፡- At present, the doctor will not examine us. We came to the health center and just show them our card and take the medication.  I፡- From the pharmacy  P፡- Yeah, we just pay and and took the medication. That’s it. There is no other thing.  I፡- There is no other thing. Eh but he have told you not to discontinue the medication?  P፡- Yeah, we will not discontinue the medication  I. What did the health professionals told you about the illness? How was it?  P. We have started treatment at butajira not here.  I. Yeah, it doesn’t matter if it is butajira  P. After that we came here and they interviewed as like this. They told us to take the medication from here. Then we went to our home. They gave us 30 30 tablets every month.  I. Eh  P. When we finish the medication, we will come and got refill.  I. Yeah, but there are doctors here who give you a service when you come every month, right?  P. Yeah, we took a medication when we came every month  I. What did they told you about the illness?  P. They haven’t do any examination  I. Haven’t they told you anything about the illness?  P. Yeah  I. Don’t you get any advice from the doctors?  P. The advice is, don’t drink alcohol (Tela, Areque), and use the medication properly  I. Is that the advice?  P. Yeah  I. Is that the advice?  P. Yeah  I. So do you think that this advice is beneficial?  P. Yeah, a lot  I. How  P. It is useful for my life.  I. What is its use for you? I mean how is it useful for you?  P. It is useful for me when I got ill since I want to be cured of my illness.  I. Let’s say for example one doctor told you not to drink alcohol, areke and also told you different things  P. Yeah I have been using that advice |
| 16 | I. how is the health workers attitude towards the patients?  P. They give advice…ehh…they use say take the medication properly; this is the physicians direction so, if she take the medication she will get better or get cured….ehh…they use to say like that. At the beginning she was taking 2 at a time…ehh…4 per day 2 in the morning and 2 in the evening but now they told her to take one at a time so she is taking one.  I. Sometimes the medication might have side effects.…yes….did the health workers inform you about the side effects?  P. The health workers?...ehh…the medication should be taken properly, one in the morning and one in the evening…ehh…it is not appropriate to increase and to decrease…ehh…they told us this when they gave us the medication. |
| 17 | I. What information did they gave you about her issue? How did they explain about her condition to you?  P. Is it this year?  I. Yes  P. They say that she will be cured with this tablet. They also tell us that she shouldn’t stop taking the drug and advise us to come and take the drug every month.  I. When You come here every month to collect the drugs, what will be done for her at that time, just as example?  P. For example, there is no injection and other thing  I. Isn’t there counseling?  P. The only advice that they give us is not to stop taking the tablets  I. who used to say that to her?  P. The physician himself  I. Is it the one who works in the pharmacy?  P. Both this one and the one who works in the pharmacy will say that |

Medication Provision

| 1 | 0 |
| --- | --- |
| 2 | I. Is there anything which should be improved?  P. I want if things can be improved here.  I. For example what things?..eee…please tell us things which you want to be improved?  P. It was health post and now it is a health center, so, if it can strengthen itself and become a hospital?  I. What different things will be here if it becomes a hospital?  P. The materials…  I. What makes you to think like this, is it related to the service?...eee…why? Is there a problem about the service delivery here?  P. There are some medications which are not available here…uhh… They used to tell me unavailability of those medications and write me referral.  I. Did they tell you could find in the hospital?  P. They use to refer me to Grarebet.  I. Are they sending you to get the medication only or is it for additional treatment?  P. Only for the medication, I didn’t get any additional treatment.  I. What treatment were you offered at the health center for the epilepsy? Was it medication? Or…  P. Okay  I. Did you get additional examination like blood test…or psycho- education?  P. No, there was no blood test.  I. Were there any other examinations?  P. No  I. How was medication provision?  P. It is difficult to get all the medications here.  I. Really?  P. Yes, even the medication I am taking now, they might prescribe for two months but the available medication can be for one month and that is for 15 days for me…eee…that is because I am taking two times per day.  I. What did the health professionals told you about the medication?  P. They use to say there is provision problem. |
| 3 | I. What about the provision of the tablet?  P. It is good  ---------  I. AS you told me there is no problem of provision with regard to the tablets, in your community in general  P. Yes there is no problem |
| 4 | I. How is it? How are you getting the medicine?  P. From health station  I. Do you purchase it?  P. Yes  I. Are you purchasing it?  P. Yes  I. Uh , is there any problem with it?  P. How?  I. ….There is no  P. No, they do not say  I. There is nothing  P. They give us every month, Every month we take the tablet. |
| 5 | P. What about the medication?  I. For example, how is the provision? Are you receiving the medication on time?  P. Yes, since I came here I never wait to get the medication.  I. isn’t there…  P. There is no… when I come here …eee…I will pay here and go there and take my medication and leave |
| 6 | I. Is there a problem in the supply of medication? Did they ever say there is no medication, wait till it is purchased and other things?  P. No, I didn’t hear that  I. No, did they give you when you came every time?  P. Yeah |
| 8 | 0 |
| 9 | I. Do you think it is good?  P. Yes, …ehh…ehh…as an opinion, they some of them give us the medication for three months once and the others may not allow by fearing there might be a shortage.  I. They are not giving once for three months  P. Yes, they are giving for two months. For example I am studing in Wolayita, and I will be there the whole summer but they gave me for two months.  I. For the summer  P. Yes, …eee… I have faced problems regarding this last year and this year, however, it is solved now and I received the medication for three months. This is happening due to the shortage of the provision.  I Are they saying that we are not giving for one person for two three months once because their might be a shortage to give for one month for all the users?  P. Yes  I. There might be a shortage?  P. Yes  I. Because there are other service users?  P. Yes…uhh…I understood it like that.... it was for one month otherwise the medication may have a tendency to expire…ehh… I will come and ask them because my place is near … ehh … ehh… the physician ordered them to give us medication for three months instead of asking us to come per month…ehh… at that time I had a discussion with them and I told them that even though it is okay in my case it could be a problem for others.  I. How to keep?  P. Yes, I wrote my comment...uhhh ... because the tablet has a biter test if it is kept for long.  I. uhhh...is it related to improper storage?  P. Yes, if it is not kept in a cold place it might has a problem. |
| 10 | 0 |
| 11 | I: so, what type of medication you received?  P: tablet  I: what is the type of medication they gave you  P: is very small tablet its like the size of lentil  I: you took only tablet? Didn’t you take an injection?  P: there is no injection, it’s the tablet  I: they gave you tablet?  P: yes, I have the tablet  I: ok, ok… is there any shortage of tablet as you are taking the tablets from here?  P: there is no shortage  I: have they ever told you that the medication is not available?  P: no, they told me to come and collect the tablets whenever I finish it. Thus, I just come and take the medication, that’s all |
| 12 | I. What did they told you about the drug? Are you getting enough drugs? How did you get the drug? Isn’t there drug shortage?  P. There is no drug shortage. For example, I finished the drug that I have on Wednsday morning and went to them in the afternoon and took the drug. |
| 13 | I: For example it could be about its supply or its side effect. E’ what did they tell you? Have they ever said they have a supply problem?  P: They never said it is unavailable.  I: Do they always have a supply?  P: They always have a supply…eh…Whenever we need the medication, when she finished what she have or when two or three left, we come and get it. |
| 14 | I. How is the drug supply?  P. The drug supply is good. They are giving me money in monthly and biannually.  I. Money?  P. Yes, they are giving me hundred birr.  I. I am not asking you about that. I am asking you about the drug supply.  P. They are giving me enough drugs.  I. Did they say as the drug is not available?  P. the drug is available all the time  I. Did they say as there is drug shortage?  P. They didn’t say like that |
| 15 | I. How is the medication supply?  P. It is good  I. Is it good?  P. We are taking when we come here.  I. Have they ever said it is not available or something?  P. Never! In my side I haven’t see that one day.  I. Yeah, it is on your side  P. In my side, it didn’t happen.  I. Didn’t it happen?  P. Yeah  I. You always get the medication  P. Yeah  I. Do you come here every month?  P. Yeah |
| 16 | I. How is medication provision?  P. Provision, they usually give us for two weeks  I. Are you coming here by two weeks interval?  P. Yes, they said it will be ruined if it staid long.  I. Okay |
| 17 | I. So, what do they told you about the drug?  P. About the drug?  I. For exmple how is its supply?  P. Supply means?  I. Do you get the drug every time you come here?  P. Yes  I. Don’t you ever lose the drug?  P. Every time we come here per month, they give us 30 tablets and warn us not to interrupt taking the drug.  I. Ehhhh  P. 30 tablet per month, it Is once daily.  I. Therefore, don’t you encounter any problem?  P. Yes  I. Regarding to getting the drug?  P. Never  I. Ehhh  P. We never miss the tablet. Every time we come here per month, we will get the drug  I.Ehh |

Side effects

| 1 | 0 |
| --- | --- |
| 2 | I. Not about that, about the side effects. The medication can bring some side effects and if you observe such effects please come talk to us….did they tell you like this?  P. Nothing like this  I. Didn’t they tell you?  P. Information about how to manage my sickness…  I. Is that all?  P. Yes, this is the only thing they usually explain…okay…I always come here with fifteen days interval to tell them what I observe and experience but they didn’t give me any additional thing other than advice.  P. My situation became worse, I only remember that I used to shout during the evening (sleeping hours)…uhh…then the red color medication…eee…I have it at home, but I am not taking it.  I. Why?  P. They told me not to take  I. Did they say?  P. Here |
| 3 | I. Why did you stop taking the medicine?  P. First and foremost, I become very dizzy, ..uhhhh….I feel lazy ..uhhh… then while I was taking I felt I am okay and my health improved, I had a lot of change for one month …uhhh… then I understand , then I become a doctor for myself and stopped it  --------  I. Apart from the tiredness, that come from the medicine you have noticed, do you know anything like the side effect of the medicine. can you give me an example?  P. I told you that before …uhhh.. When I took the medicine,…uhhhh… if I fell something in y mind , they told me to go there immediately; |
| 4 | I. Did they tell you about the side effect of the medicine?  P. Until now they do not tell me  I. Uh  P. They do not tell us like that.  I. Did they not tell you what the side effects are?  P. They told me there might be a problem if I am not going to keep the precaution.  I. Uhh did you get medicine in Butagira?  P. Is it the first time that I started?  I. Uh  P. Yes  I. How was it?  P. It was not that much…..  I. How?  P. When I take for the first time …I was feeling pain for a long time  I. Urr  P. Around six month like this but…  I. Uhh, uhh the tablet does not bring side effect on you?  P. Yes  I. Because of the medicine, there is nothing that you feel sick?  P. There is no?  I. There is no |
| 5 | I. side effect means for example when patient take a medication one person might have aching  P. Yes  I. The other patient may have other type of sickness due to that medication  P. Yes  I. For example, did you ever hear when somebody say that I get gastritis due to the medication I use?  P. Yes  I. Some people use to say that, so, did the health workers tell you about it?  P. No, they didn’t  I. Didn’t they tell you about problems which the medication might cause?  P. I did ask him about the sleeping problem…eee…I have sleeping problem  I. Didn’t they tell you to come whenever you observe such things?  P. They told me to come and discuss with them.  I. Did they tell you?  P. Yes  I. Did you ever face such type of problems due to the medication?  P. Previously, I used to felt sleepy when I took the medication but now I have sleeping problem, I don’t know whether it is related to the medication or not. |
| 6 | I. Haven’t they told you to come here if it caused you a side effect?  P. No, they haven’t |
| 8 | P. But with my work, farm work, as you saw it has tiredness  I. Ah  P. Then it made me tired and for this two month farm work and  I. Ah  P. Until it is finished, I discontinued it by my self. But the medicine is going to be very curative. That is what I realize |
| 9 | I. Did they tell you to come back if you observe side effects?  P. I didn't hear them saying that. However, what I am telling to the others is the medication will improve our condition if we take it properly; this is my perception.  I. The medication?  P. Yes, if it is kept.  I. Is there a problem?  P. Yes; okay...the other thing is if we can get support regarding this.  ---------  I. What do you say to the family members who are advising you to go to the holy water and tell you the badness of the medication?  P. I understand that the medication is not going to harm me even though ….I feel tiredness if I do much labor works and thinking. I have to get some rest if I feel that at that time they/family members might say like that  ----  P. Yes, I took 60 and then I start feeling something  I. What?  P. I start feeling something...ehh...previously it was without a symptom...ehh... but recently I am feeling dizziness and when I feel that I should sleep and take rest. I will be okay if I get rest. I consulted the health professionals when I observe that repeatedly ...ehh... I have consulted and asked them what to do after I took the medication for 15 days. And then they recommended me to take it just like previous times.  I. How about the health professionals?...eee... how informative the health professionals were about the sickness?  P. About the sickness, it is treatable if the medication is taken properly...ee...avoiding fire and gorge...ee... self-knowing (erasen mawok)... other than these the medication has no side effects ...ehh...the medication doesn't harm stomach (result gastritis) like other medications....eee...they explained and I did understand that. |
| 10 | 0 |
| 11 | I: while taking the tablets, some people might feel uncomfortable, right?  P: you are right  I: so, did they counsel you about the medication like, did they tell you that the tablet might cause you some problem and to come here if you feel like any discomfort related to the tablets? … did they tell you like this while the provide you the medication?  P: they told me to come every month and collect the tablet. They said, “you can come and collect the tablets when 2 or 5 tablets are left and don’t forget to bring your card whenever you come here”  I: didn’t they tell you about the side effects of the tablet and to come back if you experience any problem?  P: no they didn’t, I don’t lie  I: but have you ever experience any drug side effect?  P: no, I have taken it properly…ehh…I took it properly and it was nice |
| 12 | I. are you always taking the drug? Did they tell you about the side effect of the drug?  P. Yes, they told me about the side effects of the drug and what to do before I took the drug. They told me to drink milk and to eat good foods. If I take the drug in this way, the drug has no any side effect on me.  I. Did they tell you like this?  P. Yes  I. Ok, but if you faced any side effect, did they told you to return back to them? This may be to change the type of the drug that you are taking.  P. It has no any side effect.  I. What problems have you faced during taking your drug like itch and other related problems with the drug?  P. I have stomach problem and sometimes I feel irritation on my stomach. Even I feel the irritation, I will continue taking the drug because the drug is very effective and I am very eager to be free from this disease. I will treat the stomach by other things like milk.  --  I. So, how do you compare the previous treatment that you got and the current treatment?  P. Which one is the previous?  I. It may be the one that you got in Dikuman.  P. Don’t talk about the treatment that I got in the Dikuman. Leave it.  I. Didn’t you see any change in the treatment that you got in the Dikuman?  P. It has no change at all.They told me to take the drug in the morning, afternoon and at night. I haven’t seen any change for the tension rather it was the cause for my stomach problem. |
| 13 | I: Well a medication is a medication. You know that! You know about the type and that it could have a side effect. Did they give you advice to come back if it brings a side effect or did they just gave you the medication?  P: They gave us the medication and told us to take it at this time…eh…We will take the medication as they told us.  I: eh what do they told you about its side effect?  P: They didn’t say anything  I: eh has she encountered any side effect?  P: There is nothing.  I: Is it convenient for her?  P: Yes it is convenient for her. |
| 14 | I. Every drug may have its own side effect. Did they tell you about the side effects of drugs?  P. Yes, they told me and advised me.  I. How did they tell you? Please tell me examples  P. they told me to take one tablet at the bed time.  I. Ok  P. They told me to take the drug after dinner and adviced me to take rest.  I. What about other things?  P. That is it.  I. For example, some of the tablets may show irritation as a side effect and at these times you are expected to go to them to change this drug. Did they tell you about such kinds of things?  P.No |
| 15 | I. Have they told you that the medication might have a side effect and it may cause some harm?  P. Harm is from taking alcohol.  I. What do you mean?  P. They have told us that drinking ‘Areke’ or other alcohol drinks and taking the medication is harmful.  I. But they haven’t told you that the medication itself has its own side effect. They haven’t told you to come here immediately if it brought you something different?  P. Yeah they only told us to eat food before taking the medication. They haven’t told us other things.  I. Is that it?  P. Yeah  I. But, they didn’t tell you about the side effect?  P. They just told me not to drink alcohol |
| 16 | I. There can be such things. So, didn’t the health workers tell you to come here if you observe such things on your wife?  P. The health workers said that if she feels sick or show the side effects…ehh..they told us to come.  I. Did they say?  P. Yes  I. Did they tell you?  P. Yes  I. Have you ever observed such thing?  P. She has a headache …ehh…she has a bad headache.  I. Did you tell the health professionals about her headache?  P. Yes, they were give us pill for her headache in Addis Ababa.  I. Really  P. Yes  I. What about here?  P. We didn’t receive here and also we didn’t ask?  I. Didn’t you tell the health workers?  P. No  I. But they told you to tell them if there is any change?  P. Yes  I. So, why didn’t you tell them?  P. We didn’t tell them…ehh… but in Addis Ababa I used to tell them and also they used to give us  I. What difficulties are you facing due to the lengthiness of the duration?  P. I am worried it might hurt her; even though I am worried I use to take her to the health facility whenever she gets sick.  I. Has she ever been sick?...ehh…has she ever been sick?  P. Yes  I. Was is because of the medication?...ehh…Is it because of the medication?  P. Yes, sometimes she feels sick while she is on medication…ehh…so we can do nothing other than the medication.  I. It is not easy to take medication for this long, so  P. Yes  I. Have you ever think that the medication can cause a problem?  P. About the medication, it is because there is no other option…uhh… She says it is hurting her..uhh…Sometimes I use to say she can do whatever she pleased and sometimes this is the professionals direction so she needs to use the medication as she told. |
| 17 | P. Yes. When she takes the tablet with empty stomach, she complains that it has burns feeling and she will stop taking the tablet.  I. The tablet?  P. yes, she will interrupt taking the table for someday, for 3 or 5 days  I. Why?  P. She didn’t say anything. But we ask them if the medication could cause problem but they explain us well that it doesn’t cause any problem.  I. Okay  P. She always feels tired. If she eat something or drink water she will vomit.  I. Does she eat food?  P. She doesn’t eat anything  I. Eh  }P. Starting from this year, she doesn’t eat anything  I. Is it after she starts taking the medication from here?  P. Yes. She doesn’t eat anything and she says it will burn her like fire. That is why she always interrupts the medication for 5 days  I. Okay .So she will not going to feel the burning if she doesn’t take the tablet?  P. It is all the same. Weather she take the tablet or not, she doesn’t eat anything. If she takes just one bite, it will burn her stomach and she will shout all night long.  I. Have you ever told this to the doctors?  P. You mean, to these clinicians?  I. Eh  P. yes we have told them  I. Here?  P. yes we told to these clinicians  I. what did they say to you?  P. we told them and they say she will be fine  I. Okay  P. When I ask if I could take her to Butajira Hospital, they say okay  I. Eh  P. I asked them if we could take her there and do x-ray or something. And they say yes you can  I. So did you take her to Butajira hospital?  P. We didn’t take her yet. But we are going to take her.  I. Eh Eh Eh  P. This is the problem  I. If she has any additional disease they will diagnose her  P. yes. I am telling her that if we could go and they will do X-ray for her  I. Eh  P. May be  I. Even if they didn’t do the X-ray for her, another doctor will see her if she has another disease  P. They advise us to take the drug after meal, but not to take it with small food or empty stomach.  I. Ehh  P. They also advise us to take it after meal when we sleep; for the drug to work.  I. Ehh  P. They say that the patient should take the drug during bed time  I. Ehh  P. If that’s so, they told us it will not going to cause any problem.  I. Did they say it doesn’t cause any problem?  P. The tablet?  I. EHH  P. It will never  I. EHH  P. They say the tablet will never cause problem.  I. Why does she interrupt taking?  P. The reason why she interrupts is because she feels tired when she take the medicine. She spend much time at the market place, she run some small business.  I. Eh  P. She sometimes goes to addis ababa by car.  I. Ehh  P. When she feels tired, she has to eat food. But her appetite will be lost because of the tablet. She is afraid of this.  I. What does she say about the cause of the drug to her?  P. She says that the drug will hurt her  I. The medication?  P. yes  I. okay  P. She claims that the drug will hurt her. Since the medication will make her appetite to reduced, she will get tired. Because of that, she will interrupt at least for five days per month.  I. Ehh ehh ehh is it because she thought the medication will hurt her?  P. She said it makes her feel tiresome  I. Ehh  P. She said, if she doesn’t eat she will feel tiered highly.so… |

Psycho-education

| 1 | I: what did you feel when they told you that you have Epilepsy?  P: they told me that, “the illness might throw you in to an abyss, so don’t go to an area with a cliff, don’t drink “Areke”, don’t drink any alcoholic drink. Everyone will die but just take care of yourself, take care of your life and live longer”. |
| --- | --- |
| 2 | I. Tell about both health facilities.  P. Here, they use to give me a lot of advice  I. Do you mean the health professionals in this health center?  P. Yes…okay…the advice is similar  I. From both health professionals (from the health center and from Grarebet hospital)  P. The advice is similar, it has no difference I. They told you it is epilepsy, I think you knew it before you go there too…eee…so, didn’t they tell you about epilepsy and …..  P. They told me about the cautious I have to consider.  I. Did they tell you about the sickness too?  P. They told me about the cautious I have to consider.  I. What did they tell you to do?  P. No to go near to water, gorge..uhh…not to go to market and people gathering…not climb to the tree.  I. Do you think it was helpful?  P. It is helpful to be careful…uhh…but there is no change. |
| 3 | I. During the time they told about the sickness, what was the list advice or what type of counseling did you get from the professionals  P. Yes there was counseling, they told me not to be angry and depressed, not to drink to much not to  be drunk  P. They also told me not to do something like this and not to be seated near fire  I. Okay  P. They also told me not to be seated around sea water, ditch gorge and the like  I. really  P. Yes they told me all this  I. Do they told you all this  P. Yes  I. How much do you think the medicine as well as the counseling has helped you?  P. It helped me a lot, I am so Happy  ------  P. For example they told me not to stand along the car drive of main Asphalt road where cares are rushing….uhh.. they told me to take the tablet when every I have to, according to the instruction they gave me  I. okay  P. They it was very useful  I. Do they say like this?  P. yes  I. So You thinks the counseling is very useful  P. Yes it is very important …uhh… and I am very happy for it…uhh….  P. Yes you have asked very good questions, yes I have developed behaviors, I was aggressive, now I have improved it  I. How did you improve?  P. What?  I. How did you manage to improve and eliminate your aggressive behavior?  P. That was due to the counseling of the doctors  I. Okay How?  P. They said aggressively can be the cause of you frequent felling and that will aggravate your sickness  I. And then  P. But I was not considering their advice, but they also told me not to attend some feasts and crowded places  I. That means what?  P. Than after I agree with them  I. okay  P. Due to that I start to improve myself  I. okay  P. That happened due to the counseling |
| 4 | I. Did they give you advice?  P. Advice, they told the precautions  I. What are the precautions?  P. Mostly this is we are getting advice now after we have changed to Kela  I. Uha , those who are in Kela  P. Yes  I. What did they say?  P….They told me that I have to free from stress, I should take care when I am around fire…  I. Uhh, So Do you think that you are getting advantage when you are following the precautions?  P. I took care of everything after that  P. Instead of worrying……, I was thinking that this makes me better now I am very fine.  I. Uhh.Can you suggest something that needs improvement for the next time?  P. For the future, their advice is nice.  I. Uh  P. Everything is good. |
| 5 | I. What did the health workers say that your problem is?  P. They told me that “you will be fine, if you keep yourself from things like drinking alcohol… if there is anything which can make you feel stress  I. …”eee…  P. I am not drinking alcohol because I understand how it is disadvantage me and also how it clash with the medication I. How much did the health workers inform you about epilepsy?  P. for me  I. What did they advise you?  P. Just the existing things, like not use alcohols…eee… how it oppose with the medication  I. …uhh…  P. one the alcohol might hurt me directly and two the medication might not work properly  I. …uhh…  P. it is not good if I quit the medication…uhh…they told me all those things. |
| 6 | I. For you, what is the advantage of discussing with them on the table?  P. They have asked me about if I have improvement compared to the past,  I. Eh  P. They have advised me to take the medication properly and to follow it up.  I. Do you think that this is useful to you?  P. It makes me happy.  I. Are you happy about it?  P. Yeah . They told me not to discontinue the medication, not to drink alcohol, to stop using chatt and other drugs.  I. Did they tell you that?  P. Yeah they did tell me that. |
| 8 | I. What did the doctors told you your illness is?  P. They told me that it is stress. I think it is stress too. Because when I got stressed I will be…..it has a stress  I. Did they tell you that your illness is stress?  P. Yeah, epilepsy I. What did the doctors said about the cause?  P. The doctors told me to come and dispense a tablet every month. They said that you will be benefited from it and you will be cured. |
| 9 | I. How about the health professionals?...eee... how informative the health professionals were about the sickness?  P. About the sickness, it is treatable if the medication is taken properly...ee...avoiding fire and gorge...ee... self-knowing (erasen mawok)... other than these the medication has no side effects ...ehh...the medication doesn't harm stomach (result gastritis) like other medications....eee...they explained and I did understand that.  I. How did their advice (about not go near to fire, river, gorge) help you?  P. It is good for others  I. What about for you?  P. It is good for me because it might happen accidentally/I may get sick accidentally..eee...if I don't take the medication ...eee... I may get sick if I don't take the medication...ee... a nurse in Yirgalem Hospital..ee....I will never forget ...ee.. it is unforgettable...ee...she told me that I shouldn't have any addiction even tea, coffee..uhh... never to have alcohol..uhh...I might get sick whenever I couldn't get...uhh...I never forget this.  I. So  P. When I observe it on myself, I understood it is bad.  I. Do you think their advice were helpful for you?  P. Yes, for example not be addicted with alcohol..even tea...  I. Is the addiction the problem?  P. If it is an addiction ...eee... it will not work if I take the medication  I. So do you think it helped you to control your sickness?  P. Yes,...eee... yes, it helped me to work; even though we become old education is not going to aged ...ee...I continued my education even though I get old.  P. The service delivery here is …  I. Is it better?  P. Yes  I. How it is better?  P. I think it is related to capacity problem  I. How?  P. The service provision..eee… the way they are giving the advice and questioning..  I. What?  P. If they can ask the patients about his/her condition an opinion since everyone has its own opinion. |
| 10 | I. My question here is that when they reduce the amount from eight to two and then to one and then to half what was the reason to decrease?  P. I don’t know  I. Do they tell you their reason?  P. they don’t tell me  I. When the professionals reduce the amount do they discuss with you?  P. They don’t discuss with me!  I. Really, but your father was accompanying you to the health center, wasn’t it?  P. Yes he was going with me  I. Then what did your father say when they reduce the amount  P. Nothing, he just say it is reduced take it .. , yes uhh… he did not tell about it  I. he said it is reduced and take it, which was the only thing you were informed?  P. They did not tell me the result, and also about my status, the status of your sickness? |
| 11 | I: what did the health workers here in Dikuma told you about the illness?  P: they told me not to drink alcoholic drink and to take the medication properly and they aslo told me that I will be normal if I can take the medication properly and aslo to take the tablet every month  I: do you think their advice has helped you?  P: ofcourse it is…ehh… it has helped me a lot. I would have been died if I wouldn’t get their counseling…ehh…that’s all |
| 12 | I. What did they tell you about this disease? What things did they tell you to do for the disease?  P. They gave me the drug and told me to take the drug according to the instruction. That is it.  I. Didn’t they tell you other things?  P. Other things?  I. Didn’t they describe about this disease for you?  P.Do you mean the health workers? The disease has no any diagnosis. I told them in the same way as am telling you. They will give me the drug and as I told you they told me as the disease will vanish one day in the future |
| 13 | I.Has the health professionals explained you about the illness and its treatment or did they already do that?  P: Yes, they give us lots of explanations. They ask her how she is doing, if she felt any illness, whether she wants to change the medication or what she wants  I: So do you think that they told you about the illness properly?  P: Yes, very much  I: What additional things would you like to know about the illness?  P: well…  I: I mean things you would like to know but they haven’t explained it to you  P: No, there is nothing they don’t explained. There is nothing more.  I: So they explained to you about the illness using words that you can understand  P: Yes, yes  I: So, they will sit down and talk to you  P: Yes and they also told me and advise me that there is no problem and to let her enjoy. They gave me so much advice.  I: eh what did they advise you to do?  P: They told me to relax indulgence her since the illness don’t like stress  I: They told you that P: yes  I: But do you think that is useful?  P: Yes, I think it is useful. ……eh…If you do something different from the usual, in addition to giving your love, there will be surely a difference.  I: Is that how you think?  P: Yes, I also think like that. |
| 14 | I. What have you heard in your area and in the health facility about the causes of this disease?  P. They said nothing  I. Are you saying as the cause of the disease is unknown?  P. They didn’t tell me about this.  I. Ok, in what way the health professionals are telling you about this disease?  P. They know the disease  I. Yes, they know the disease but how did they tell you about this disease?  P. They told me as the disease has drug and to take the drug according to the instruction and in addition to this they also told me not to drink alcoholic drinks and local drinks such as ‘Areke and tella’. So, I am getting better thanks to God. |
| 15 | I. Eh eh what did they told you about the medication?  P. Do you mean how it should be used or  I. About how it should be used, its side effect,  P. About how it should be used, we have been taking it at the evening one tablet per day. They have given us for 30 days so I took one when I am going to sleep  I. Eh. Then you sleep  P. Yeah  I. Have they told you that the medication might have a side effect and it may cause some harm?  P. Harm is from taking alcohol.  I. What do you mean?  P. They have told us that drinking ‘Areke’ or other alcohol drinks and taking the medication is harmful.  I. But they haven’t told you that the medication itself has its own side effect. They haven’t told you to come here immediately if it brought you something different?  P. Yeah they only told us to eat food before taking the medication. They haven’t told us other things.  I. Is that it?  P. Yeah  I. But, they didn’t tell you about the side effect?  P. They just told me not to drink alcohol.  -----------  I፡- So what is your experience? What are your suggestions about the health service delivery here?  P፡- I think it is good.  I፡- But you have told me before that apart from giving medication, they wont review or examine you  nt about that?  P፡- When we come here every time, it is good if they would give us some advice and review our health status in addition to giving the medication. They haven’t told us anything. They haven’t asked us how we are doing and about our health status. Thy just gave us the medication and we will go to our home. There may be a problem on this.  I፡- Eh what advantages will you get from it?  P፡- We can protect ourselves. We can tell other patients who haven’t get the service here about the illness and other things.  I፡- Give me example  P፡- Not all of the patients get the service today  I፡- Yeah  P፡- When I go to my home, I could give information to the patients in my neighborhood (if there is any who didn’t get treatment) about the illness  I፡- You are going to tell them?  P፡- Yeah, So that he protects himself from becoming ill. But we could do that if they gave us the advice  I፡- Are you saying that you can give the awareness to others when you have the knowledge.  P፡- Yeah, it is difficult if they didn’t give us the advice.  I፡- You are right  P፡- If they gave us the advice it will be useful to me as well us to people in my neighbourhood.  I፡- So you are saying that they should discuss with you and give you advice?  P፡- Yeah, there will not be a problem if they give us that. Most people in my neighborhood do not have the information. Some patients have got the service here. But there are still some patients who did not get a service here. I don’t know whether those people have the information or not. They have invited me today while I was at work. I have stopped what I was working and came here. But if I have gone to other place by chance, they wouldn’t invite me.  I፡- That was for the interview. I am asking you about the service delivery here?  P፡- I have told you that they didn’t deliver us a service.  I፡- You are right, Okay  P፡- If they have delivered the service to us, it would be useful for me as well us for my neighbours. But they haven’t delivered it. |
| 16 | I. What did the health professionals say her problem is? What did they say her sickness is?  P. They said it is epilepsy (Yemitel beshita), it is stress... Ehhh...They said like that.  I. Did they say like that?  P. Yes, the medication should be taken daily and if she has no improvement she has to get another examination and the medication should be changed …ehh… they said the medication comes from abroad  --  I. Did the health professional give you an explanation about her current condition?  P. Yes…ehh…they give us advice, not to let her to drink alcohol, not to chew chat and drink coffee, because it affects the medication. ..ehh….they usually tell us to take the medication properly and come back the appointment day |
| 17 | I. My question is Do they explain to you in detail to let you understand it very well?  P. They will try to tell us again and again. If we don’t understand, there is someone who can understand well both amaharic and guragigna and then translate for us.  I. Ehh  P. This person will sit there and will translate for us  I.Ehh  P. He is helping us very well. Therefore we understand it very clear.  ---  I. Therefore, about her disease and her condition…  P. Yes  I. Do they tell you these very clearly?  P. Yes, they told us not to take her to any traditional medicine or to any other religious place.  I. For example?  P. They just advise us to follow up here in the hospital  I. Did they tell you not to use traditional medicine?  P. Yes, they told us not to use traditional medicine  I. The physicians?  P. Yes  I. You mean the physician who works here?  P. Yes, they told us that traditional medicine is harmful  I. Ehh, what did you response then?  P. If she keep on taking this medical treatment and she remain the same, other means of treatment might be used. They advise us just to use this tablet only.  I. Didn’t they tell you about her current situation clearly?  P. Yes Yes… Very well  I. What do they say?  P. They will ask us starting from the very first point when the symptom occurs for almost one hour. They will also ask us about the risk factor for the occurrence of the symptom.  I.Ehh  P. Depending on her condition |

Health workers attitude/skill

| 1 | 0 |
| --- | --- |
| 2 | I. How is the health workers sympathy for you?  P. They usually help me  I. Do they have sympathy?  P. Yes…uhh…they keep watching me  I. Do they respect you?  P. us….we do respect them.  I. When we say respect, how is their hospitality for you? Are they treating you like others (patients with another type of sickness or anybody?)…eee… do they disrespect you because you are a patient?  P. No,  I. What do you mean?  P. What I mean is they usually try to provide the service for us quickly.  I. Do they care for you?  P. Yes…uhh… something like that…uhh… there is nothing else.  I. So, How did you feel about not talking to the health worker about your problems?  P. I don’t feel good, but they are busy to do this, so I simply came here and take my medication.  I. Do you mean that you are not happy for not discussing about your difficulties with the health professionals?  P. No, I am not. |
| 3 | I. I am asking you as a patient? … uhhh… as I told you, I need to tell me the experience from attending the medical treatment, the waiting in the medical facility the reception from the professionals and the like  P. You know what it looks like; there is the same thing they do always, …uhhh… they have to stay in their office, that is their duty, someone who is seated in the office should do a lot of things, ..uhhh…, do you understand for example .. if I am working at the office …uhhh… if I am a social judge in the court …uhhh… when I had a lot of files … uhhhh… I will be tired of numerous files, and I may be upset by those files, …uhhh… and if someone asked me how my reception is I may not notice how I acted, because I am busy with my work; I cannot spoil the work of the other and others career  I. Yes I am not asking to spoil the work and career of others; but to ask you how was the service?  P. You know how the service was?  I. Yes  P. Yes, they are providing the service  I. Was it good?  P. Yes they provide good service; they receive me with good care, surprisingly they are very good and I am very happy  I. You don’t have such worries, so it is only when you come to kela or is it the same in all health facilities?  P. Thanks to him, wherever I go all the nurses and doctors have good reception for me …ehh.. I am observing it based on my experience..ehh.. they have good reception. |
| 4 | I. Why do you go “Kella”? Why do you not come here ?  P. At first I went there.  I. Are you going there because you adapted going there or are you thinking that there is service difference?  P. Yes there is difference.  I. How?  P. Their duties are different from them.  I. How?  P. From the workers ………………  I. Do you think that the service is better there?  P. It is better.  I. Is it their potential?  P. Yes, their potential too.  P. Their advice too and everything.  I. Those who are working there are better than these workers.  P. Yes  I. Why do you think that those are better than those workers?  P. ……not only this medicine……….  P. Another, when I bring my child for medical service they are not that much……..  P. They are not well skilled.  I. Do you have communication?  P. Yes  I. Do they respect you? Or do they treat you like other patients? Do they treat you differently than other patients since you are mentally ill?  P. Yes, It is like other sickness, they do respect us and teat us.  I. Do they respect you?  P. Yes  P. As I guessed  I. Uhh,not only this there is another thing  P. Another thing  I. Is it medical service?  P. Yes  I. Uhh, uhh how do you relate this? Is it the training problem?  P. I think they cannot.  I. Really?  P. it is like that I think so |
| 5 | I. How sympathetic the health worker was towards you?  P. Yes, they are doing this because they have sympathy for me  P. Are you asking me about the doctor?...eee…he told me something even though I forgot some…eee…I can say I get benefit. |
| 6 | "I. When you come here, how was the reception of the health professionals?  P. It is good, it is very good.  I. Really  P. I don’t have any bad experience up to now.  I. Did they feel sorry for you?  P. Yeah  I. Did they respect you?  P. They will tell me to get in and sit down on the chair.  I. Have they said like that?  P. Yeah  I. Okay, When they give you the health service, I mean when they gave you the medication, have they told you about your illness and have they asked you a comment or suggestions about what you need to do or shall they gave just the order?  P. No  I. Didn’t they?  P. No, they didn’t  I. Yeah  P. When I finished the drug exactly after a month, I will come and take the medication  I. Did they just give you the medication?  P. No, for someone who can understand some  I. But they don’t let you to participate.  P. Yeah |
| 8 | 0 |
| 9 | P. No, I told you the service delivery here is better than Dukuman.  I. Is it because they are doing it in rush? Rushed consultation  P. I was perceived it that they became like that because they were staying with mentally ill people and then I decided to come here.  I. Are yirgalem and Amanuel better than this?  P. Not Amanuel; here and in Yirgalem  I. How about in Amanuel?  P. There is rushed consultation in Amanuel too  I. Is there?  P. Yes, I suspect the health workers are acting like that because they are working on mentally ill people and adapt their (mentally ill people's) behavior.  I. so the service delivery here is better than there?  P. It is not inviting there. I gave them my clear comment for them …ehh… because if a mentally person came there the condition in that hospital may worsen that person’s sickness.  --  I. How is the service delivery here, I think this is the fourth health facility which you visit because of your sickness?  P. Yes  I. How is it?  P. It is fine  I. The service provision?  P. It is good specially their hospitality.  --  . How did you find the health professionals hospitality? How sympathetic or respectful the health professionals were?  P. Yes, when I come here I don't wait long. When I was working on the rural area I used to come here on Saturday. They were on duty; they were not leaving early even though it was their day off ..ehh... I usually start working on Monday and come to the city on Friday and spend the night and go to the health center on Saturday. However the health professionals use to tell me that it is good if I can come on Friday afternoon because I might not get medication sometimes and quit taking for three days (the weekends).  I. So they have sympathy and respect?  P. Yes; however, sometimes some health professionals who yemiyabshaketu  I. Are there such people?  P. It didn't happen to myself it happened to other people with other types of sickness.  I. Was it with patients with other sickness?  P. Yes  --------  . Does it have a difference?  P. Yes; …ehh… previously in Butajira …ehh…. I have observed disrespecting and rushed consultation.  I. Was it in Dukiman?  P. Yes …ehh… rushed consultation, I think it was because there were many service users  I. Was it difficult?  P. In my opinion and understanding if a person stays with mentally ill people that person will be like them. I observe this thing in Amanuel too. This is my assumption.  I. Okay, this is your assumption?  P. Yes  P. It was long time ago but still I feel things should be improved  I. It is true  P. Because a patient should not be treated this way/in a rush. We went there to get treatment for our sickness but being treated like dirt…. They should respect whatever that person is. Showing a good face is the beginning of everything.  I. Were they disregarding only mentally ill people or all of the service users?  P. There are some people ,,,ehh…. When they bring the card …ehh … or in the consultation room …ehh… they tell for the people to go here and there.  I. It wasn’t only for mental health patients?  P. As I told you it could be because of the condition  I. How do you think about the health professionals’ competency?  P. They are to doing what they are told regarding mental health not more than that  I. I am asking you how the health workers competency to deliver the service here?  P. In my opinion they are qualified to give the advice and medication.  I. Are they qualified?  P. Yes  I. Do you think?  P. Yes  I. Don't you have any doubt?  P. No, I don't. |
| 10 | I. Do they tell you their reason?  P. they don’t tell me  I. When the professionals reduce the amount do they discuss with you?  P. They don’t discuss with me!  I. Really, but your father was accompanying you to the health center, wasn’t it?  P. Yes he was going with me  I. Then what did your father say when they reduce the amount  P. Nothing, he just say it is reduced take it .. , yes uhh… he did not tell about it  I. he said it is reduced and take it, which was the only thing you were informed?  P. They did not tell me the result, and also about my status, the status of your sickness?  I. Do you think that it is from the proficiency of the professionals or from the efficiency of the medicine?  P. I don’t know about it really |
| 11 | I: Yes, when you come here for your appointment…ehh…don’t you converse with the doctors?  P: they talk to us very well…even last time when I came here, he asked me, “how are you? Are you feeling better”?  I: were you entered his examination room?  P: yes, he talks to me…he asked me whether I am feeling better or not he also said that, “you have improvement”, he talk to me a lot  I: …ehh… what do you say about the health workers who are providing the service here…ehh…how do you see their attitude? How is their empathy or approach to you? How do you see the respect they show for you?  P: that is very nice, I am so happy about that. I am very happy by their counseling, for me after I came here and started the medication I just become a normal person  I: how do you see the capacity of the health workers? How do you assess their capacity?  P: what?  I: their ability to provide treatment?  P: ok  I: what do you say about that?  P: what do you mean by treatment?  I: do you think they have the ability to treat peoples?  P: yes, it very very nice ...ehh...its very nice  I: ok  P: its because of them that I am still live |
| 12 | I. Do the health workers in the health facility respect you?  P. Yes, properly  I. Are they humble?  P. Yes. They asked me about my health condition, about the benefit of the drug, about the side effects of the drug and other related questions. |
| 13 | I: What is your opinion about the capacity the health professionals in treating patients?  P: What do you mean by opinion?  I: I mean do you think that the health professionals are competent to treat her illness?  P: Yes, they are very competent.  I: Do you think that a quality service is being delivered here?  P: Yes  I: We have to discuss the truth. What we discussed here will not be disclosed to anyone.  P: There is no problem. Really! If there is a problem, we will tell you the problem. There is no problem.  I: Isn’t there a problem?  P: There is no problem….eh…what we need apart from getting a service. They serve us well. |
| 14 | I. How do you see their attitude towards you and other patients?  P. Their attitude is good.  I. Are they treating you properly? Are they humble?  P. When I came to this health facility, they are treating me properly and when I need tablet, they will give me with out any problem.  I. So, you mean as they are good.  P. Yes  --  I. So, what is your experience regarding the service provision/treatmet in this health facility? Is it good?  P. They are receiving us in good way.  I. Is that true?  P. Yes, it is true. I swear.  I. How do you see the capacity of health professionals in providing the treatment service?  P. They are treating properly and I haven’t seen problems  I. Do you think as they are capable?  P. Yes  I. Do you think as they are providing quality service for patients?  P. Yes, they are working properly.  I. Do you have any comment in the health service provision which needs improvement for the future?  P. I don’t have any more comment for improvement.  I. Is it enough?  P. For me yes  I. Do you think as it is good?  P. Yes, they are working properly.  I. Do you think as the service provision which is given here as good?  P. Yes, they are providing the service properly  I. Do you have any additional thing that you want to add?  P. I don’t have  I.Eh eh so it is because of huge number of patients not due to the health center’s problem?  P. No, it isn’t  I. It isn’t. Eh eh  P. When we come, they ….. in good way.  I. Treat you  P. Yeah, there is no problem in their side. |
| 15 | I፡- In your opinion how is the capacity of the health center staffs in treating your illness?  P፡- They haven’t examined me here. I just took your medication.  I፡- Eh, So  P፡- They haven’t asked us other questions  I፡- So you are saying that you don’t know anything about their capacity  P፡- Yeah, I don’t know anything |
| 16 | I. How is their respect?...ehh..how is their respect?  P. The health workers?...ehh…The health workers are friendly/comfortable/for us, they give us advice…ehh I. Do you think the health professionals here are capable to deliver the service?  P. Yes, we rely on them till now. |
| 17 | I. How is the health professionals’ respect?  P. It is nice  I. I mean their respect towards you?  P. That is very nice  I. Really  P. Thanks to the holy trinity, we are praising since our children learnt and reached to this level. We don’t have to go to other places; we consider it as if we are at home.  I.Ehh  P. Yes, we assume as if we are at home  I.Ehh  P. They are welcoming for us with a great respect and smile, that makes us feel happy. We praise the lord.  I. Ehh  P. There is nothing that makes us feel unhappy  I. About the medical service provision here?  P. The medical service is better and efficient.  I. How?  P. Their efficiency is very good. They never undermine people; they say nothing but welcome us in nice manner.  P. The treatment?  I.Ehhh  P. They are welcoming for us  I. Is it nice?  P. Yes, it is nice  P. It is the same with the Amauel once. However..  I. Ehh  P. Amanuel’s treatment has helped us for certain years till now  I. Ehh  P. Thanks to God, those tablets are also available here; which is near to us. The tablets are the same.  I. Ehhh  P. The probable difference might be on the doctor’s status. In addition, she used to attend there with her sister. |

Continuity of Care

| 1 | 0 |
| --- | --- |
| 2 | I. Are you getting the same people or different people?  P. I get different people |
| 3 | I. When you come here on your appointment day…ee… are you going to be seen by the same/different health professionals?  P. The same person  I. The same person?  P. Yes, it is the same person.  I. So do you think seen by the same person is advantageous or do you think it is better if you can be seen by different people?  P. Do you think a person you are familiar with is the same with not familiar, for instance one of them could be touchy ...eee...and the other could be a person who will be on his desk on time.  I So do you think seeing by the same person is better?  P. It is good if they have similar behavior...ee... I would like if I get a person I am familiar ith...ee... a doctor I am familiar with...ee... I like her... I mean her reception...ehh... but if a different doctor comes I may not know her behavior....ee.. she is not laughing, she is not speaking... if she is silent... I gave an answer for the questions she asked ...ehh... she asked me to stay long, do I suppose to stay long on the sun...so I would be happy if we can see the same doctor.  I. So are you saying seeing the same health professional is better?  P. Yes...ehh... this my opinion regarding this |
| 4 | I. Uhh,uhh Are you meeting the same physician when you come on the appointment date. or is there any change ?  P. No, they maybe different or the same.  I. Uha, It is not known.  P. Yes  I. Uh, how? How did you get it? Is there any difference when you meet different physicians? How did you see it?  P. How because of the change?  I. Is it good if they are the same person or if they are different?  P. It is better if they are changed.  I. Why?  P. When he gives advice there may be different idea.  I. Ok  P. It is good when they are changed.  I. Is it good when they are changed?  P. It is good when they are changed.  I. Uhh. What if it is the same person?  P. If they are the same, their question will be the same ,After asking the first question ,he will go to the same question |
| 5 | I. When coming for follow-up appointments, did you see the same health worker each time?  P. Yes  I. When you come here  P. The doctors  I. Whenever you come here  P. I know one doctor, I know him, I do remember his look  I. Did you always see him?  P. I know them  I. Or did you see different persons?  P. I think I did see different people.  I. What do you mean? Did you see different health workers when you come here last month and the month before that?  P. No  I. Did you see the same person?  P. I came two times I think I saw one person.  I. In your opinion which one is better, seeing by the same person or seeing by different people?  P. If it is possible to get the same person…eee…seeing by person is easy.  I. Is it good?  P. Yes, it is  I. How it is helpful?  P. Because, if that person (the health worker) knew about me and also if I am familiar with that person…eee…we can discuss in detail.  I. Okay  P. Otherwise that person might get confused until become familiar with it. |
| 6 | P. I might not check about the doctors  I. You may not know them  P. I don’t usually look at a person.  I. Don’t you know if it is the same doctor or not  P. The persons, me  I. Eh  P. I just show them my card and take my medication |
| 8 | 0 |
| 9 | P. I see different health workers  I. Do you?  P. Yes  I. So do you think always seeing the same health worker is better or seeing different health worker?  P. In my opinion seeing the same health worker  I. Do you think it is better?  P. It is would better if it could be the same health worker until the end.  I. Why?  P. I believe that person will help the patient until the end.  I. Do you believe that?  P. Yes, I believe that…ehh…the information is written and documented...uhh… anyone can understand but if it is the same person he/she may take it as his/her own, that is why. |
| 10 | 0 |
| 11 | I: but when you come here for follow up, do you want to be seen by the same doctor or different doctor?  P: different doctor  I: how?  P: its good to ask how  I: ok  P: he is a doctor right? He is a doctor…but I was almost dying last time…ehh…because I was very terrified, I have seen one or two doctors…after that I have seen different doctors  I: thus, which one do you prefer? To be treated by the same doctor or different?  P: I don’t mind if any doctor treats me  I: that’s not a problem?  P: yes, that is not a problem. I just want them to take care of me |
| 12 | I. As you told me earlier, you are always getting the service from one health worker. What do you think about this? Which one is good? Is it to get the service by one health worker only or with different health workers?  P. The drug is the same.  I. The drug and the treatment is the same but which one is good for you? Is it getting the same health worker or different health worker?  P. As I told you before, he gave me the drug for the first time here and if I get another health worker may be the drug will be changed and I may be sick by other disease if another person gave me different drug.  I. So, you prefer him.  P. Yes, he is always giving me the drug. |
| 13 | P: They allocated an individual called somebody for her. He is the one who followed her up and treat her…..eh’…that is him  I: And also does she often get treatment by the same doctor or by different ones?  P: Here? I: Yes  P: If he is available, he treat her sometimes. If he is unavailable, others could treat her. We have a treatment card. When we give them that card, they will look at the card and at the paper and will give us the medication. |
| 14 | I. Is the health worker the same person who is asking you every month? Or, are they different?  P. They are different  I. Are they different?  P. Yes  I. Which one is your choice? Is it different health worker or the same?  P. I like different health worker  I. Why?  P. Because at one day one doctor will ask me and the next day the other will ask me. In this way, I may get better thing.  I. Better thing  P. Yes  I. What if, if he is the same?  P. No problem.  I. So, you have no problem in both ways.  P. Yes |
| 15 | I፡- When you come here often, do you get the health service from the same person or from different persons?  P፡- It could be different person.  I፡- Eh, the one who give you advice?  P፡- Yeah  I፡- It could be different person. But in your opinion is it better to get the service from the same person or from different person?  P፡- I will be happy if I get the service from the same person.  I፡- Why?  P፡- Since he is going to assess my problem and explain to me about it and my behavior, it is better if it is the same person. I think if the person is changed every time his behavior and everything will be changed too.  I፡- So you think it is better to get a service from one person who knows you better?  P፡- Yeah, I will be happy to tell him my problem too. |
| 16 | 0 |
| 17 | 0 |

Discrimination from Health workers

| 1 | 0 |
| --- | --- |
| 2 | I. Are they discriminating you?  P. There is no discrimination.  I. isn’t there?  P. No…uhh…discrimination?  I. Isn’t there such thing?  P. They care….okay… they usually care about us not discriminate us. |
| 3 | I. Okay, how is stigma and discrimination?  P. There is no  I. Really  P. There is no  I. Really  P. They can’t discriminate me for example, they might try to discriminate me however I am educated person |
| 4 | I. Do they respect you? Or do they treat you like other patients? Do they treat you differently than other patients since you are mentally ill?  P. Yes, It is like other sickness, they do respect us and teat us.  I. Do they respect you?  P. Yes  I. Uhh, Is there any discrimination?  P. There is no discrimination.  I. There is no?  P. There is no. |
| 5 | I. uhh… is there discrimination?  P. in other places  I. Is there discrimination in other places?  P. At my place  I. Discrimination means like this person has epilepsy…eee… discriminating that person because of his sickness?  P. Other people  I. The health workers  P. The health workers..eee… there is no such thing. There are good things here |
| 6 | I. Is there a stigma and discrimination?  P. I don’t know  I. You should tell me what you know. Don’t worry about others  P. Ehhhh  I. What you know means when you came here, did they for example handle roughly, look down, and discriminate?  P. Up to present, I don’t now but I didn’t see anything  I. Isn’t there?  P. There isn’t. |
| 8 | I. Did you have any bad experience?  P. Bad experience  I. I mean because of this. Because of your illness  P. Yeah  I. When you came here to got treatment?  P. No |
| 9 | (about Ammanuel) P. It was long time ago but still I feel things should be improved  I. It is true  P. Because a patient should not be treated this way/in a rush. We went there to get treatment for our sickness but being treated like dirt…. They should respect whatever that person is. Showing a good face is the beginning of everything.  I. Were they disregarding only mentally ill people or all of the service users?  P. There are some people ,,,ehh…. When they bring the card …ehh … or in the consultation room …ehh… they tell for the people to go here and there.  I. It wasn’t only for mental health patients?  P. As I told you it could be because of the condition |
| 10 | 0 |
| 11 | I: do you notice any stigma and discrimination?  P: never, I never see that…ehh… I have seen only good things not other than that  I: …they might inform on you like this, “those are mentally ill persons”, and the like?  P: no, I never heard like that…ehh…they only consel us good things |
| 12 | I. Is there any stigma in the health workers?  P. Nothing. Please elaborate it.  I. For example, they may not treat you equally with other patients that came for other health services because they may discriminate you as you are mentally ill people.  P. No. They are treating us properly. |
| 13 | I: Is there some kind of stigma?  P: There is nothing. I swear! AS soon as we come they give us soft drinks….eh….as soon as we come….eh…there is no problem. |
| 14 | I. So, you mean as they are good.  P. Yes  I. Is there stigma?  P. No, there is no stigma |
| 15 | I. Is their stigma?  P. What do you mean?  I. What I mean by stigma is to treat you differently because you have a mental illness  P. No, I didnt heard such thing  I. Isn’t there?  P. Yeah, there is nothing  I. So they did respect you like other patients  P. Yeah, a lot  I. Eh  P. We didn’t see anything. |
| 16 | 0 |
| 17 | 0 |

Confidentiality

| 1 | 0 |
| --- | --- |
| 2 | I. As you know the health workers usually write patients history …okay…do you ever been worried about the confidentiality of your personal information? Or have you ever been worried about the health professionals exposing your personal information for others?  P. For another person?  I. For example, a health professional examine you….  P. another person…  I. He/she is going to ask you many questions…okay…then he is going to write that…uhh…that will be between you and your physician…okay…then did you ever suspect that personal information of yours could be exposed for another health workers or other people?  P. No, I don’t  I. So, you think your physician will keep your personal information confidential?  P. Yes |
| 3 | I. There is an issue that the information should be kept confidentially  P. Yes yes  I. So have you been worried about its confidentiality? Do you ever think there might be storage problem?  P. No  I. Really?  P. Truly I don’t think like that …ehh… never  I. Doesn’t it concern you?  P. What can I say to you more than this  I. Okay Okay…so you are not worried , is that because you think they are not going to expose the information to others or is it because you don’t care whether they expose it or not?  P. No No it is right  I. What do you mean?  P. They are keeping it properly  I. Do you think they will not expose it to the third person?  P. No, they are not …ehh.. they are very good people |
| 4 | I. Uhh,uhh as you know your personal information will be recorded on the card it is necessary for the medical service process.  I. What do you think about this process? Do you think that they will show for other person? Or Do you think they will reveal your secrete for other person?  P. Emmm, I am….  I. Why do you not say?  P. Whether they revealed or not, I. I do not give attention for this kind of things.  I. Ok  P. I am thinking about my illness, I am thinking how I am going to cure but I do not give attention for that. or ……I do not think that if they reveal, I will feel a shame.  I. You do not have.  P. Yes  I. So, you never think?  P. Yes  I. but do you think that they will reveal your personal information?  P. They won’t give.  I. Uh  P. They will not give. |
| 5 | P. Here in the health center?  I. Do you think they can expose your personal information for others?  P. No, I don’t  I. Don’t you think so?  P. No, I don’t. It is mandatory for me to tell them frankly.  I. You have to tell them frankly, but if they expose for …  P. If they tell…  I. Do you think they can do that?  P. It is okay if they do.  I. Don’t you worry?  P. No, I don’t.  I. Don’t you have any problem if they disclose your personal information?  P. I don’t if it is in here but I do if it disclosed for the people in my community…eee… I don’t want them to expose my personal information.  I. So, you don’t want your personal information to be disclosed?  P. No, I don’t want to.  I. But, have you been concerned?  P. No  I. Haven’t you been doubt them?  P. The health professionals, no never doubt them and it is not appropriate….uhh… because I discussed with them about my problem…  I. Yes, you did but a health worker took your personal information  P. Yes  I. Did you have any concern about exposing that information for others due to mishandling of that health professional?  P. I don’t have any concern that the health center exposes my personal information. |
| 6 | P. They did write on it.  I. Did you ever think that they might show that record to others?  P. No I didn’t  I. Eh  P. I don’t think so. I don’t think it will happen. They did write infront of us at the card. Then they wrote on the cover of the medication and gave us.  I. So you don’t think that the will show it to others?  P. I don’t think that  I. You never think like that?  P. Never  I. Really  P. I never think like that.  I. Ehhh  P. Well, only a man knows about himself.  I. What do you say if they gave it to other person?  P. Eh  I. How do you feel if others saw it? Are you going to be happy or not?  P. He may read the confidential information written there. He won’t get other things. Even if he gets something, it won’t be useful for him |
| 8 | I. They will write a note about your personal informations and did you ever think that they are going to show it to other people?  P. No, I don’t  I. What if they show it?  P. I am happy. I am not going to say any thing. Nothing will make me to back off. I’m not ashamed of it. |
| 9 | I. As you know when a person come to the health facility for treatment the health professionals will take that person's personal information  P. Yes  I. How that person is sick and also other related information, so do you ever think your personal information may not be keep confidentially?  P. No, I don't  I. Don't you think it could be exposed to others?  P. I use to tell people with this condition about its treatability …ehh… I am taking my medication about things other than this….  I. Are you saying it is okay if it is exposed to others?  P. Yes, it has no problem. |
| 10 | 0 |
| 11 | I: well, as you know when an individual came to a health facility, that individual’s private information will be recorded, right?  P: that’s right  I: for instance, he/she will be inquired and the information will be recorded on the card  P: yes  I: have you ever thought that the health workers might disclose your confidential information to other persons or have you ever frightened that, your private information might be disclosed to other persons?  P: I never thoght like that  I: why not?  P: nothing like that… they just counsel me not to drink any alcoholic drink, that’s all …ehh…they don’t have any fault |
| 12 | I. When you came to this health facility, was there an experience of asking the patient personal information and recording it on the card? For example, they may ask you many things. Have you asked them about the privacy of your information? You may think as somebody will see your information. Do you think like this?  P. I don’t think about that whether they showed the information for others or not. The card that they gave me is with me and I will bring this card when I came to the health center in monthly basis. |
| 13 | 0 |
| 14 | I. When you came to health facility, the health professionals will write your personal information on a card.  P. Yes  I. Do you have any fear about this information? Probably, you may think as they may show your information for another person.  P. I don’t fear.  I. Why?  P. Why should I give it for others?  I. I am not saying like that but as you know the health professionals took your personal information and write it on a card.  P. Yes  I. Do you think as they may give your information to other persons due to information keeping problems?  P. I don’t think like that.  I. How?  P. They took my information and it will be useful only for me. It will not be used for other persons.  I. Do you think as they didn’t show your information for others?  P. Yes, they didn’t show  I. Did they keep it in a secured way?  P. Yes, they will keep it in good condition.  I. What if another person sees your information? Do you want?  P. No, I don’t want.  I. Don’t you want?  P. I don’t want  I. Why?  P. For example, you gave me this paper.  I. Ok  P. What do you think if I give this paper for other person? I think it is not right  I.So, you don’t want to expose your information about your disease for another person.  P. Yes, I don’t want |
| 15 | I፡- So the doctor did recorded all your personal informations, right?  P፡- Yeah, about my problems like this  I፡- Yeah, do you think that the health center or health center staffs or the doctors will keep your personal informations properly?  P ፡- Do you mean keeping my personal information? All personal information is personal.  I: Yeah, yeah  P፡- Yeah, I think they have taken the information like you to keep it  I፡- Do you think that they will keep it properly? I am not asking you about us, I am asking about the health center? When you go to the health facility and get a service, the doctor will write on the card what your illness is, your age and other details about you  P፡- Well, they wrote on the card only one time that is when we came here first. After that we just pay and take our medication. That is it. There is nothing.  I፡- Isn’t there other thing?  P፡- No, I haven’t heard anything. |
| 16 | 0 |
| 17 | 0 |

Follow up

| 1 | I. You didn’t come here after your first visit?  P. No, I didn’t  I. What was the reason?  P. Nobody asked me to come, they told me to finish the medication and it has been a month or 15 days since I finished the medication…uhh…I am not going to be absent every month if they ask me to come.  I. So, you didn’t come here because nobody asked you to come?  P. Yes, I never heard.  I. Was there another reason?  P. No, there wasn’t. |
| --- | --- |
| 2 | I. After your first appointment, were you given another appointment? Did you attend?  P. I had been here yesterday even.  I. By what time interval are you coming here to take your medication?  P. Every month  I. Are you coming here every month?  P. Yes  I. With whom are you coming here? And since when you have been coming here?  P. I came here and take my medication last Sunday.  I. Not like that, the health professionals use to give you an appointment to come here every month…  P. Yes |
| 3 | I. After you came to the health center you have been given an appointment?  P. Yes  I. Have you been given an appointment?  P. Yes …ehh… on my appointment day  I. Are you following your appointment?  P. Yes  I. You do miss some appointments  P. Don’t say like that to me  I. If a patient has an appointment that person should come based on the given appointment.  P. That person might have a problem for example transport problem…eee… everything might not be perfect every time… yes sometimes…  I. Appointments will be given…ee… do the health professionals encourage you to come based on your appointment?  P. A father or a mother can’t be like them |
| 4 | I. So are you coming based on your appointment?  P. Yes  I. You come on the appointed date.  P. Yes  I. How is it? Do the workers encourage you to come on the appointment date?Do they remind you?  P. Sometimes based on the appointment……sometimes based on the number of tablets, sometimes they will call us if they need us to give advice. Monthly, we are taking for a month.  I. Uh  P. After that I am coming and taking every month.  I. Uhh, how are they? Do they encourage you?  P. It is like this, yesterday there is no appointment, now  I. Uh, not like this, do they tell you to come when you come to take medicine?  P. For me it is not told, I have a card, based on the card  I. Are you coming based on that?  P. Yes |
| 5 | I. After your first appointment, were you given another appointment?  P. Yes  I. Did you attend?  P. I didn’t bring my wife when I came here  I. That happened recently, but when you come here after your first visit…  P. While I am taking the medication…ee… I am taking without any problem  I. Have you been coming here every time?  P. Yes  I. Did the health workers encourage you to come back?  P. Yes |
| 6 | I. Eh eh. After your first visit here, are you coming for follow up on your appointement?  P. Yeah  I. Did the health professionals advice you to come here for follow up and not to discontinue it?  P. Yeah, they did  I. Eh  P. They did tell me to come every month and take my medication |
| 8 | 0 |
| 9 | After your first appointment, were you given another appointment?  P. Yes  I. Did you attend your follow up?  P. Yes  I. Do the health professionals encourage you to come?  P. Yes  I. What do they say to you for example?  P. For example, they use to tell me to come per month before I finish the medication  I. Do they tell you?  P. Yes |
| 10 | 0 |
| 11 | I. well, did they gave you appointment during your first visit?  P: ok  I: they gave you an appointment, right?  P: yes  I: so, what did they tell you? Did they encourage you to come on your appointement date?  P: ofcourse!  I: the doctors?  P: yes, very much  I: what did they tell you  P: the doctors?  I: yes  P: they told me to come back before I finished all the tablets and to bring the appointement card while I come for follow up…ehh… they also told me that they will provide me the tablet when I come...ehh… |
| 12 | I. Do you remember the first day that you came to this facility? They gave you an appointmet for treatment.  P. Yes, they appointed me.  I. Did they tell you to return back?  P. How?  I. What did they told you to return back at your appointment day? Did they motivate you to come in your appointment day?  P. Yes, they told me to return back on my appointed day.  I. Did they tell you that?  P. Yes  I. Are you coming every month?  P. Yes, I am coming every month. |
| 13 | 0 |
| 14 | I. As you know, they will give you the next appointment after you came for the first time. Is it right?  P. Yes,  I. By how many times are you coming to this health facility?  P. Me?  I. Yes  P. I will return back when I finish my tablets.  I. By how many times does it mean?  P. the drug that I took will be enough for about a month  I. Are you coming every month?  P. Yes,  I. Ok  P. I will come every month and they will give me the tablets.  I. Are the health workers encouraging you to come in your appointment day?  P. They came to my house and call me.  I. Is that?  P. Yes, |
| 15 | P. We came here today because they have told us to come  I. This is for this interview. What I am asking you is when you come here to get a service?  P. We haven’t been referred to other place.  I. Do you always get the service here?  P. Yeah, once we have referred form butajira and started treatment here  I. Eh, do you come here by referral from butajira?  P. Yeah, I came from there. I have started treatment at butajira “Dikumane”. After some time they invited me to come here.  I. Okay  P. They invited us here and interviewed us like this. Then they gave us a service. After that we are taking the medication from here. We haven’t gone to other places.  I. You haven’t gone?  P. Yeah  I፡- Eh eh, when you came here for the first time, they did give you an appointment, right?  P ፡- Yeah  I፡- Did you attend on your appointment?  P፡- Yeah, we come here every month  I፡- Do you come every month?  P፡- Yeah  I፡- Do they encourage you to come every month?  P ፡- Yeah |
| 16 | 0 |
| 17 | P. Even, in times when we were unable to come on the appointed day and lost for one or two days due to certain constraints, they will ask us the reason why we didn’t come on the exact date.  I. Ehh  P. They will ask us like that  I. Will they send message to you or will they asks when you come over here?  P. They will send us message. For instance, there is this girl who lives around the farm…  I. you mean is she health worker?  P. Health what?  I. The health extension worker one?  P. They will contact her over the mobile phone and send us message. she will then come and tell us the message.  I. Does she tell you?  P. She will walk around and tell us the message |

Clinical impact

| 1 | I. What is your view of the idea of providing care for people with mental disorders in the health centre?  P. It is good…uhh…it is good; it helped me, previously I had sleeping difficulty…okay…but now I am sleeping well, I had to sit in one place and not move around as I wanted to, but now I go to coffee shops to drink coffee. I got better after I came here.  I. Do you believe giving a service here is helpful?  P. Yes…okay… yes, I got better because of this service…uhh….I got better due to this service.  I had been taking that medication in the evening after dinner, I was told to take it like that.  I. Okay  P. I never missed to take my medication; I got cured after I took the medication, thanks to Him [God]. People were asking me how I got better.. |
| --- | --- |
| 2 | I. Yes, you are not going to measure their capacity regarding the basic science they are practicing, but do you think that they are treating you well?  P. I will see this by dividing in to two  I. Okay  P. I couldn’t see their capacity on me…eee… I didn’t see any change/improvement on my condition…eee… so I am not satisfied at all.  I. Did the medication improve your condition, for example you used to faint frequently, and does the medication bring any change and help you to do your job? Or difference on your condition before and after the treatment?  P. Yes, the frequencies get reduced…it is per a week now.  I. Is the frequency reduced because of the medication you are using?  P. Yes, it gets reduced after I started using the medication.  I. As you told us you were receiving the service in Gerarebet before you came here, so, do you get the service provision here as you expected?  P. About the medication change…. I observe some changes on my condition after they changed the medication…eee… the duration becomes less now, previously I could be unconscious for an hour but now it reduced to 15 minutes. |
| 3 | I. So we have to learn from you, to make the service a better one for you and for others  P. Okay  I. That is the aim of the investigation  P. Okay I understand that, therefore, while I am taking the medication …uhhh…. I fell very strong and fell that I can do whatever I want  I. So you think you are improved  P. Yes I am, the sickness is improved |
| 4 | I. Uh  P. ….Up to now I did not see any change on the medicine that I have started now.  I. What about the previous one?  P. The previous one also was late………..  I. Uh  P. First, when I was feeling sickness, I was taking the medicine but when I stopped , I was feeling sickness before. But after that I was taking the medicine every day and I was fine for more than six month.  I. Uh  P. When I take this medicine, I did not face any problem up to now.  I. Uhh,uhh before you start the medical service and now after you start the service, do you think the treatment changed your behavior and your work habit ?Do you keep yourself and have good relationship with others.  P. Yes  I. Is it after you got treatment and medicine?  P. Uhh,yes  I. For example, how?  P. How is it? Before I was feeling bad, I was feeling tired………  I. Ok  P. But I got the advice and I am following the advice so I am in good condition.  I. Are you in good condition?  P. Yes  I. Uhh, for example can you tell me the improvement?  P. ………Before, I was much stressed.  I. How?  P ……Previously I was working farming and I had good life.  I. Ok  P. But after I caught this sickness , I became weak and I could not do anything.  I. Ok  P. I have responsibility on the family but I could not lead my family.  I. Uh  P. I could not do like others.  I. Uh  P. I could not ,this makes me feel…………  I. Is it after you started the medicine?  P. Yes  I. Ok  P. but later on I have started the medicine and I have seen improvement, I stopped worrying about things, I have started doing my job.  I. Do you think that the advice helped you?  P. Much |
| 5 | P. I came here and start the follow up and the medication. They are telling me it has been reducing…  I. Really  P. My families…  I. The frequency has been reducing?  P. Yes, it has been reducing  I. ….uhh…  }P. previously I used to fail down three or four times per month, however now my wife told me that I am failing down only if there is rain…eee…when I ask her |
| 6 | P. My current medication didn’t help me. As you know I couldn’t able to work and support my self. As he said I do some things and take a rest. I had a seizure every day. I am suffering a lot and so I couldn’t work  I. Eh, do you have a seizure every day now?  P. Yeah, I have a seizure every day. Because of this I couldn’t work and pay my rent for my home. |
| 8 | I. Ah Ah Then you have said that you have a plan to come back here  P. Ah  I. Why do you want to come back?  P. Since the medication have an effect.  I. Ah, did it heal you?  P. Yeah  I. Did the medication heal you?  P. Ya it has an effect  I. Ah  P. I am not totally cured. Some times I got ill. But I think this is my fault since I stopped the medication  I. Ah  P. I want the medication since I have seen when people benefited from it  I. Ah  P. I am going to take it since I got benefit from it. I am going to take it |
| 9 | I. uhh..uhh... how useful is the treatment?  P. It is very useful...eee... because it is a problem which makes you to lose your mind …ehh…so controlling the condition by taking the medication and the advice will be helpful. For example me, I use to feel ashamed when I wake up because I don’t know what I have done when I was sick. |
| 10 | What about the provision of the medicine, the health facility in the health center, the service provision concerning this sickness called headache (Azurit), how do you see it and evaluate it in your opinion?  P. Yes! I saw it tow three times, there is not effective result  I. Do you believe the health facility is useless? How and why?  P. I took the medicine for about eighteen years, I have very long years of experience in this medicine but no result  I. Do you really take the medicine this long?  P. Yes I took the medicine for about eighteen years, it is not new for me, I took it for about eighteen years and not improvement, no change  I. was there any change in your health status  P. Yes there is no change, after I took it is the same I will fell again and again, and then I will take it will come again in the next day  I. How was the procedure you took the medicine? Did you stop in middle and then continue  P I was taking the medicine, four tables every day, okay .. then I did this frequently but I was still fell down after I took the medicine  P/ Yes.  I. Then tell us about your situation and about you sickness?  P. concerning my current situation, I am taking the medication, and the main problem is I fell frequently  I. Are you taking the medication and still the problem continue  P. Yes, I am taking the medication but I am still suffering the problem, I fell, I took the medication |
| 11 | I: do you think this illness can be cured with treatment?  P: ofcourse. I really recognize it…thanks to you all …ehh… I would have been died if I didn’t take the medication. Last time a guy in Mekele died while trying to wash his cloth in the river, he was having the episode and he drowned in to the river while trying to wash his cloth there. I would never get closer to a river or to an abyss…ehh… now I am normal, I will not go to a river or an abyss with anybody.  I: so, do you think the illness can be cured with medical treatment?  P: yes, I think it can be cured with this tablet |
| 12 | P. Yes, I see something in my heart.I have started drug in Dikuman but I didn’t see any change but the new drug is very good and I am getting better. Before I start this new drug and at the time when I took the previous drug, they will come at the place where I slept, on my way or in my house. They will do what they like to do.  I. As you told me before, you saw monsters. What do you mean?  P. I became tensioned. It is only tension and it is on my head. Something made me to be tensioned on my heart. Currently, I am good due to the current drug. Thanks to God, I am getting better. Currently, I am taking the drug but sometimes I became tensioned. Sometimes I saw them and at that time I prayed to my lord and called the name of the lord and at that time they disappeared. I know if this thing is understood by other person, I may not fall after starting this drug.  I. didn’t you fall before due to the disease?  P. Yes, something made me to be tensioned. It is like tension on my heart and my mind. I saw something at that time and called the name of Lord and they went out. My problem is tension.  I. Your disease, the epilepsy disease. Do you think as it can be curable by treatment?  P. Yes, it can be cured by treatment. The new tablets are very effective and I am getting better. I am taking now two (three) types of drug. The one seems similar with the drug which came from Dikuman. The two drugs are new  I. Have you received the drugs here?  P.Yes, I got the drugs here and I am getting better as a result of these drugs.  I. You don’t know after you fall but do you see savage before you fell down?  P. Seeing savage is the result of the tension  I. Did you see them at those times?  P. Yes, I see it every where. I will see it now and then it will disappear. But it is decreasing  I. Is it decreasing?  P. Yes, the current drug is very effective and I am getting better. Previously when I see them, they didn’t go from me. I saw them as they sat on me.But the current is somewhat different from the previous. When I see them, I will call the name of the Lord and at that time they will go from me.  I will go to them as soon as I finish my drug. As I told you earlier, I will take one tablet in the morning and four tables at night. As they told me, the drugs are very important also for depression and hallucination. This drug is very effective. I saw the change. Thanks to God, I am getting better and better. |
| 13 | I: What did the health professionals say the illness is?  P: They also knew what her illness is. They identified her illness by assessing and examining her. They always examine her. Then they said her illness is this and prescribed her a medication. Initially she got treatment at Butajira, dikuman. …eh…The medication prescribed her at dikuman helped her a lot though it didn’t heal her much. She brought much improvement now when they changed it to this [looks like the respondent is showing interview the medication].  I: Does she get well?  P: Yes, she got better.  I: What do you think her problem is?  P: Well, her problem is her illness. Regard to her illness, she is fine now since she is taking a medication; since they prescribe her the medication  I: What is her illness?  P: Epilepsy  I: epilepsy? P: Yes I: Eh  P: She had a mental stress. Since she is taking the medication, she is very well now… Since the medication they are giving her is helping her a lot, she is very well.  : As you told me earlier you received a service at dikuman as well as here. How do you compare it with the service given here?  P: This one is good. That one is also good. Both are good.  I: Is there any difference between them?  P: Yes, This one is very good. The difference is this one is very good.  I: In what way is this better?  P: The medication. The medication she obtained is not only one. They gave her two types of medication. They gave us three different types of medications. It is very effective. In the past she don’t used to sleep properly. But currently she sleeps properly. She don’t disrupt my sleep as well.  I: She sleeps the whole night?  P: Yes, she sleeps tightly. |
| 14 | I. How do you see your ability to wok when you were sick and after you start the drug? Is it improving?  P. I am getting better  I. How?  P. I am not falling down  I. When? Is it currently or in the previous times?  P. It is after I started taking the tablet  I. So……  P. I am fine after I started taking the drug  I. How due you see the difference before and now?  P. I am currently fine  I. How?  P. I am not falling  I. What about before?  P. Previously I was very angyy and at that times the disease will start but currently after I started this tablet, I am fine and leading my house properly.  I. What was your feel when you hear the first time as you have epileptic disease?  P. I heard my case as epilepsy the first time when I fell down and after that I came to health facility for treatment. They diagnosed me and gave me tablets. When I finish my tablets, I will come and take tablets.  I. How do you see the treatment? Is it good?  P. Yes, it is good  I. Ok,  P. I am getting better  I. What things are you benefited from their advice? Tell me examples  P. I became healthy after I came to this health facility because they gave me tablet.  I. Are you getting better?  P. Yes, I am getting better |
| 15 | I. How much is the treatment useful?  P. I got here because of the treatment  I. Eh, so you are saying that it is good?  P Yeah, I said it is good. |
| 16 | I. Did she get better after that?  P. After she visited the health professional…ehh…yes.  I. As you know this medication has to be taken for long time so what do you think about the usefulness?  P. Its usefulness, relative to the difficulties I had been through…ehh…it is better. |
| 17 | I. Previously, you have said that it would be better if the drug let her be health by taking it once, why you said that?  P. Yes, in my opinion the convulsion and the shouting are not gone completely. It is just because she is following up the tablets that she is fine now.  I. Do you think the symptom will start again if she stops taking the drug?  P. Yes, I think so. I think if she stops taking the drug the symptom will come again. I truly worried about.  P. There is rumor that they are send from Addis Ababa. After that time, we even have transport compensation. We come here and take the tablet .she is very well now. She no longer falls, except that she couldn’t eat Enjera.  I. Is she healthy now, since she is taking the drug?  P. Yes, she is now free from the convulsion and the shouting |

Functioning

| 1 | I. What is your view of the idea of providing care for people with mental disorders in the health centre?  P. It is good…uhh…it is good; it helped me, previously I had sleeping difficulty…okay…but now I am sleeping well, I had to sit in one place and not move around as I wanted to, but now I go to coffee shops to drink coffee. I got better after I came here.  I. Do you believe giving a service here is helpful?  P. Yes…okay… yes, I got better because of this service…uhh….I got better due to this service |
| --- | --- |
| 2 | I. So, do you think the information which the health professionals provide you was helpful?  P. It was not helpful for me till now, I use feel a pain on different parts of my body…my nerve… it is holding me from work.  I. Is it?  P. Yes…uhh…I have been observing signs but not change.  I. It didn’t bring a change on your sickness.  P. I am doing nothing…when I was studying it was difficult to do math and I did complete the exam by guesswork.  I. Okay  P. When I feel sick  I. Was it because of the subject (math)?  P. Yes, I was not able to do math and physics  I. Was it difficult for you?  P. It was difficult for me, then I did complete it by guessing and tried to calm myself…uhh…so as not to get sick.  --  I. How about your economic status…. Is there any change regarding this.. are you productive?  P. No I am not doing that  I. Aren’t you selling plants?  P. I am selling  I. how is the market?  P. It is not this much  I. Are you performing it now in a better way than before?  P. Yes, I am starting  I. Are you okay now?  P. Yes  I. What type of sapling are you producing?  P. Sapling which can be a big trees  I. As you told me you are selling plants, did you ever cover your medication expense by yourself? Or are you working better after you start taking medication?  P. Yes, I am better now comparing to the previous |
| 3 | I. So you think you are improved  P. Yes I am, the sickness is improved  I. How is your relation with other people, as you are a farmer?  P. Yes I am  I. You are working every time in your farm?  P. Yes I am a farmer  I. Then as a farmer  P. Yes I am working  I. Does the medication; bring some impact on your income  P. Yes very very much, look what happened …uhhh…. I will take the medication……uhh… and then I take care of my children …uhhh…there are also things to take care of the fertilizer, I have to pay my debt, I have the skill of painting walls, I work that , Thank God I do my job while I am taking the medication, if not …  I. If not you will not go to work?  P. Everything is dark |
| 4 | P. But after I caught this sickness , I became weak and I could not do anything.  I. Ok  P. I have responsibility on the family but I could not lead my family.  I. Uh  P. I could not do like others.  I. Uh  P. I could not ,this makes me feel…………  I. Is it after you started the medicine?  P. Yes  I. Ok  P. but later on I have started the medicine and I have seen improvement, I stopped worrying about things, I have started doing my job. |
| 5 | I. How is your ability to do your job?  P. I am not this much; I think it is related to my other health problem  I. Doesn’t the medication improve your condition or aren’t you better than before?  P. No, it doesn’t improve my condition recently…eee….It doesn’t, I have other problems too.  I. Your  P. I am not normal  I. Do you think it is not the sickness (epilepsy)?  P. I think I am the one who is letting my sickness aggravate |
| 6 | I. Eh Eh, after you have started the treatment, how is your work capacity?  P. I told you before. I work some small things like carrying small things  I. As compared to the past, do you have some improvement interms of work capacity?  P. Slightly  I. If you don’t used to work small things in the past, are you working now?  P. For instant, there are some days where I sleep the wholeday.  I. Till now?  P. Yeah, sometimes I feel that if I go outside I will fell down on a stone so I ill slept the wholeday. When I feel relaxed, I will go out and work some small things |
| 8 | But with my work, farm work, as you saw it has tiredness  I. Ah  P. Then it made me tired and for this two month farm work and  I. Ah  P. Until it is finshed, I discontinued it by my self. But the medicine is going to be very curative. That is what I realize P. Now, I am going to come back in my own time, to the medication  I. Ah  P. I need it because now I got some gap in the farm work  I. Ah  P. I told you before. Since I got some gap, I will come back. I am not going to do that work if I don’t stay alive.  I. Ah  P. My mind is telling me that First I have to give priority for my life so I have to use the medication  I. Ah  P. I said I am going to take it. |
| 9 | I. uhh...uhh...How is your working capacity after you start the medication, is it improving?  P. Yes  I. Is it improving?  P. Yes, I am upgrading my certificates.  I. Was it since you start the medication?  P. Yes...uhh...uhh... in the previous times I was a driver...ehh... I got sick when I get my second level diving license...ehhh... I stopped that and join education sector...ehh.... and then I start working as a teacher after a one year training. |
| 10 | 0 |
| 11 | I: as you told me, you were sick previously  P: yes  I: you also told me that, you have improvement now  P: yes  I: what about work? Do you work now?  P: its very nice  I: didn’t you work before?  P: I was working but I was afraid the illness might begin  I: but, have you ever get sick while working?  P: yes, it was happening …I feel dizzy, I sit when I feel dizzy because I was afraid I might fall down  I: at the work place?  P: yes, I was scared that I might have frothing of saliva just sitting there …ehh…I feel like the illness might do something bad to me  I: thus, are you saying that the medication has helped you to do your job properly?  P: yes, very much  I: thus, you are saying that its nice they are asking about it?  P: yes, that’s very nice and I am happy about that  I: what does that help you…can you give me an example?  P: previously since I was drinking “Areke”, it was disturbing my sleep but now I am sleeping well…ehh…plus, previously I was very disturbed, not feeling good but now I sleep all the night and I wake up in the morning then, I have one cow and I collect grass for the cow, I go to work and I get my money for the day…ehh…I go back to home and if they tell me that there is no coffee, I will give them money…I get thirty birr for the day work here but it might be forty or fifty birr…ehh… then I after I get home I wash my legs and go to sleep |
| 12 | I. Ok, how do you see your working ability in recent times? Is it improving?  P. How?  I. For example, how do you see your working ability at home? Is it better when compared to the previous times?  P. I was very sick in previous times and now I became exhausted. When I think about work, I became depressed.  I. How do you see your working ability? Is it improved or not?  P. I became tired.  I. Do you become tired?  P. Yes, I become exhausted. That is it.  I. Yes, as you told me you became tired but how do you compare it with the previous times? Are you currently working or not?  P. Previously, I was working evening I was sick. Before I went to Buee, I lived in Kella. I was working at that time even though I was taking the drug. I worked at those times but now I am tired.  I. Are you tired?  P. Yes, I am tired.  I. So, you are not able to work.  P. Yes, I am unable to work. I am tired.  I. Are you buying it from others?  P. Yes, My income source is only my husband and I don’t have any other source other than him.  I. Are you contributing for your house life improvement?  P. how do you mean?  I. Do you have any thing to mention which is your contribution and can be mentioned as the effect for the improvement of your life?  P. I do nothing  I. Didn’t you do anything?  P. He is doing for our home. I didn’t do anything. I don’t have any better things in my life. I don’t worry about my life. My worry is only for my health. |
| 13 | I: How is her current working status? Does she work?  P: She is fine at the house hold tasks. In the past she don’t used to work at all. But now she bake injera and also carry out other house hold chores.  I: Does she carry out those tasks? P: Yes  --  P. She went to a market, she don’t used to know anything. She used to throw away the things she had and the money I gave her for the market and she came back. …..Okay… She is fine now.  I: Can she properly exchange things in the market now?  P: Yes, now she can go to the market, exchange properly and return. So that means she has higher improvement.  I: Significantly….. P: Yes |
| 14 | I. If you are sick, your life may be disturbed. How is you life now?  P. My life is good.  I. Is it improving or what?  P. It is good.  I. Is it improving?  P. Yes  I. What do you think the reasons for your life improvement?  P. The reason for my life improvement is my health condition improvement.  I. Are you working now?  P. Yes, I am working.  I. Did you work in the previous times?  P. I didn’t.  I. Why?  P. I fear to work because I think as I will fall down on wood or other things  I. What about now?  P. I am good now.  I. Are you working now with out fear?  P. Yes  I. So, are you saying as this brought change in your life?  P. Yes  I. Do you think as it has change in your expenses?  P. Yes, I am working and getting money and it is useful for my house. |
| 15 | ፡- Eh Did you get any special improvement because you have taken the medication?  P፡- Yeah  I፡- For instant, in terms of work  P፡- Yeah, I am working well now  I፡- How about in the past?  P፡- I used to scare in the past.  I፡- Why?  P፡- Because I got scared that I may have a seizure and fell down. I even don’t used to go to some places alone.  I፡- Okay  P፡- I used to feel scared when I go to some places. After I have started taking the medication and the holy water, Thanks to God, I have started going to anyplace I want to go and work. I have started working what I want and come back home.  I፡- How about now?  P፡- I can go alone now.  I፡- Really!  P፡- Yeah I don’t feel scared anymore  --  - Eh so do you start coming to health center by yourself?  P፡- Yeah I can go to any place.  I፡- By yourself  P፡- Yeah I can go to any place I want to go.  I፡- So you also come to the health facility by yourself  P፡- Yeah  I፡- You don’t need help from anyone  P፡- Yeah I don’t. I am working my work alone. I can work anything I want. I can go to the market and do anything.  I፡- So you are saying that your work capacity is increasing now?  P፡- Yeah, a lot  I፡- How is you income?  P፡- Well, overall our income is low.  I፡- But what I mean now is in related to your illness.  P፡- Yeah, in the past I don’t used to work much due to my illness. But now I am working to improve my income. I want to have high income.  I፡- So are you saying that you are working now properly compared to the past?  P፡- Yeah Yeah I can do any farm now.  I፡- Really! Eh so you are saying that it is improved?  P፡- Yeah |
| 16 | I. How is your wife’s working ability? Is it improving?  P. If she is sick ..ehh… she feels tired and dysfunctional..ehh…not able to move….okay…so, she feel tired.  I. is it not improving?  P. No, sometimes it is the same.  I. There is no change at all?  P. No, but if she takes the medication…ehh…she has to take the medication whether it has side effects or not…ehh…taking medication is the main thing…ehh... if she doesn’t take the medication she will fall down and it will create another problem….ehh…it is like that. |
| 17 | P. we can have our daily food expense. And we can buy cloth, may be, once in a year.  I. Actually, that is what happens in every house  P. Yes, It is true  I. So, regarding her sickness, do you think your life has progress after she start taking the medication?  P. Yes, of course, thanks to God. I don’t actually have that much farm land. I got monthly profit of less than 1500 after paying tax to church and to the government. It is not that much that I get. So she covers our expense because she is working at market. We live by wisdom.  I. So is she the one who covers much of the house’s expense?  P. Yes  I. Eh  P. Yes. She covers the expense for coffee and the like. |

Community Participation

| 1 | I. What is your view of the idea of providing care for people with mental disorders in the health centre?  P. It is good…uhh…it is good; it helped me, previously I had sleeping difficulty…okay…but now I am sleeping well, I had to sit in one place and not move around as I wanted to, but now I go to coffee shops to drink coffee. I got better after I came here. |
| --- | --- |
| 2 | 0 |
| 3 | I. But you are related with people, there is association, for health, for agriculture and the like, you will be gathered with people  P. Yes we gather  I. Did you participate?  P. Yes I participate  I. Therefore can we say you capacity for social participation is also increased?  P. Yes, it is even I can see I have more capacity to participate, It improved me a lot more  I. How  P. Yes It has given me a lot of confidence, before I was taking the medication I was aggressive and I was not able to communicate properly, but now I have changed a lot …uhh… I was angry with anything but now I am cool  I. You think you are alright in this regard?  P. Yes I am  I. You have good interpersonal relation with the farmers and other people  P. Yes I am |
| 4 | Ok, ok ……As you told me you are farmer, so I think there is meeting and discussion in your village.  P. Yes  I. Is there any difference on your participation before you take, while you are taking and after you took the medicine?  P. On me?  I. Yes, are you going to participate .  P. Yes  I. For example, how?  P. When I was taking the medicine, I was not arguing or giving idea I just simply attend the meeting.  I. Is it always?  P. Yes  I. Is it previously and now?  P. Yes,, before but now I am trying to improve my mental health. |
| 5 | I. As you know there Idir, farmer association…and those things demand community members’ participation, so, how is your participation in those activities?  P. I do have participation ...eee…but there some activities which couldn’t go together with my health condition…for example, meetings…ee...if I participate there…eee….I might raise some questions or others might raise at that time I usually don’t feel good.  I. What do you mean?  P. I don’t want to go too  I. Why?  P. On a mourning… if there are many people it disturbs me … it is not only because of my sickness.  I. You don’t want to?...how is your participation? Is it improving and how the others accept your participation?  P. The people are good, they family but I might have worries?  I. You do have worries?  P. Yes, if I fall there…ee… people might say, he is sick or if something happens to him and being with him…. By thinking those sayings might make me angry….  I. Do you think they see you down?  P. Yes, those things..  I. So, aren’t you going because those things?  P. I usually don’t go, I don’t want them to see me like this.  I. You are not going because you don’t want to or?  P. Yes  I. Is it because you are not able to go?  P. If they see when it happens it is difficult with the society, it might be difficult to live in peace. |
| 6 | I. How is your ability to meet with peoples and chat and play with them?  P. Eh  I. Compared to the past, is your ability to meet with peoples and chat and play with them improving?  P. Yeah I don’t usually actively chat with people.  I. You don’t?  P. Yeah  I. Why  P. There are a lot of things to ….with people.  I. Like what?  P. Some persons are good. Some are bad. I will go to my relative, the one I told you before, and will stay there and watch TV. |
| 8 | 0 |
| 9 | 0 |
| 10 | 0 |
| 11 | I: what about your interaction with peoples? Participation in farmers’ association and so on?  P: that is vey nice  I: you have improvement?  P: very much  I: do you participate in meetimgs now?  P: ofcourse  I: didn’t you participate before?  P: no, because presviousely I was afraid the illness might occur there  I: ok  P: I was even afraid of peoples while talking because I feel like they make fun of me…ehh…I was very terrified because I felt like I might fall over there  I: what about now?  P: now, everything is nice after I started the follow up |
| 12 | How is your communication with other persons?  P. How do you mean?  I. It means your relationship with the women that are living in your village. Do you discuss with them and other persons about different issues? How do you see your relationship with them? Is it improving or not?  P. I like to be with others in the previous times.  I. How is it? Is it improving or not? As you know it is due to the disease.  P. Yes,  I. You may like to be with others but you may fear as you may fall due to the disease. For example, previously you felldown due to this disease and at that times your relationship with others may decrease. What about now? Is it improving? Are you communicating with others or not?  P. I am communicating with the community.  I. Do you have good communication with the community?  P. Yes,  I. I am asking you about your relationship with others. Is it improving or not?  P. I am getting better. I am communicating with others and with our neighbor. So, my communication is improving from time to time. |
| 13 | 0 |
| 14 | 0 |
| 15 | 0 |
| 16 | I. Do you think her sickness affects your social activities like wedding, mourning, edir…and the like?  P. Yes, wherever we go whether we go to a wedding or a mourning ...uhh... I have to help her to relax and not worry even though it is in the neighborhood.  I. Have you ever been in able to attained because of her?  P. Me...ehh.. No, I will take her....ehh... My attention will be with her |
| 17 | I. What about her working condition or the communication that she will have with other people at work place?  P. Yes  I. Does she participate in Edir or Mourning in your locality? Does her participation in these social gathering improves or in progress?  P. Considering her current status?  I. yes  P. yes it is true. She attends every social gathering in our locality. But, of course, sometimes she might say she is tired.  I. So are you telling me that she is in better condition because of the medication?  P. Yes  I. Eh |
